# Negative interactions determine *Clostridioides difficile* growth in synthetic human gut communities

**DOI:** 10.15252/msb.202110355

**Published:** 2021-10-25

**Authors:** Susan Hromada, Yili Qian, Tyler B Jacobson, Ryan L Clark, Lauren Watson, Nasia Safdar, Daniel Amador‐Noguez, Ophelia S Venturelli

**Affiliations:** ^1^ Department of Biochemistry University of Wisconsin‐Madison Madison WI USA; ^2^ Microbiology Doctoral Training Program University of Wisconsin‐Madison Madison WI USA; ^3^ Department of Bacteriology University of Wisconsin‐Madison Madison WI USA; ^4^ Division of Infectious Disease Department of Medicine School of Medicine and Public Health University of Wisconsin‐Madison Madison WI USA; ^5^ Department of Medicine William S. Middleton Veterans Hospital Madison Madison WI USA; ^6^ Department of Chemical and Biological Engineering University of Wisconsin‐Madison Madison WI USA

**Keywords:** *Clostridioides difficile*, computational modeling, ecological interactions, pathogen invasion, systems biology, Biotechnology & Synthetic Biology, Microbiology, Virology & Host Pathogen Interaction

## Abstract

Understanding the principles of colonization resistance of the gut microbiome to the pathogen *Clostridioides difficile* will enable the design of defined bacterial therapeutics. We investigate the ecological principles of community resistance to *C. difficile* using a synthetic human gut microbiome. Using a dynamic computational model, we demonstrate that *C. difficile* receives the largest number and magnitude of incoming negative interactions. Our results show that *C. difficile* is in a unique class of species that display a strong negative dependence between growth and species richness. We identify molecular mechanisms of inhibition including acidification of the environment and competition over resources. We demonstrate that *Clostridium hiranonis* strongly inhibits *C. difficile* partially via resource competition. Increasing the initial density of *C. difficile* can increase its abundance in the assembled community, but community context determines the maximum achievable *C. difficile* abundance. Our work suggests that the *C. difficile* inhibitory potential of defined bacterial therapeutics can be optimized by designing communities featuring a combination of mechanisms including species richness, environment acidification, and resource competition.

## Graphical abstract

A combination of bottom‐up community assembly and computational modeling reveals determinants of *Clostridioides difficile* growth in synthetic human gut communities.

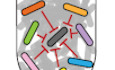

## Introduction

Interaction with native members of human gut microbiota inhibits the ability of gastrointestinal pathogenic strains of *Clostridioides difficile*, *Salmonella enterica*, and *Escherichia coli* to secure an ecological niche and cause infection (Buffie & Pamer, 2013). The importance of colonization resistance by gut microbiota has been particularly highlighted in *C. difficile* infections, where treatment with fecal microbiota transplants (FMT) from healthy donors has proven astonishingly effective in eliminating the symptoms of *C. difficile* (Dowle, 2016). Because FMT has notable risks including the transfer of antibiotic‐resistant organisms, potential associations with flares of inflammatory bowel disease, and in rare cases death (Wang *et␣al*, 2016; Chen *et␣al*, 2018; DeFilipp *et␣al*, 2019), defined bacterial therapeutics that have been well‐characterized and standardized are needed to improve the safety and reproducibility of living bacterial therapeutic treatments. However, a key challenge to the design of effective and safe bacterial therapeutics is the vast design space of presence and absence of hundreds to thousands of potential organisms. Improving our understanding of the ecological principles of community resistance to *C. difficile* invasion could guide the design of maximally effective and safe therapeutics.

Multiple synthetic communities that inhibit *C. difficile* either *in␣vitro* or *in␣vivo* using murine models have been identified (Tvede & Rask‐Madsen, 1989; Lawley *et␣al*, 2012; Petrof *et␣al*, 2013; Buffie *et␣al*, 2014; Ghimire *et␣al*, 2020; Pereira *et␣al*, 2020). The majority of the defined communities are found by screening reduced complexity communities composed of isolates from a stool sample. The isolates are combined either randomly or selected based on phylogenetic diversity (Lawley *et␣al*, 2012; Petrof *et␣al*, 2013; Ghimire *et␣al*, 2020). Other *C. difficile* inhibiting communities have been more rationally designed based on predicted mechanisms of resource competition (Pereira *et␣al*, 2020) or statistical analyses of human and murine gut microbiome data that identify taxa that correlate with infection resistance (Buffie *et␣al*, 2014). However, the design process for therapeutic synthetic microbial communities frequently does not exploit information about interspecies interactions or molecular mechanisms. A deeper understanding of the ecological and molecular principles of communities that inhibit *C. difficile* could inform the rational design of therapeutic consortia.

In macroecology, there is a long history investigating principles of invasion that has been more recently applied to microbial systems (Mallon, van Elsas, *et␣al*, 2015). Invasion theory has identified four fundamental processes that determine the outcome of an invasion: dispersal, selection, drift, and diversification (Kinnunen *et␣al*, 2016). Biotic selection has been shown to be a key determinant of the outcome of an invasion, wherein higher diversity communities can competitively exclude an invader by reducing the availability of ecological niches and efficiently utilizing resources (Dillon *et␣al*, 2005; van Elsas *et␣al*, 2012; Ketola *et␣al*, 2017). However, community biodiversity does not always correlate with invasion outcome, as other biotic interactions (*e.g*., production of antimicrobial molecules), abiotic selection factors (*e.g*., environmental pH, resource availability) and dispersal, drift, and diversification processes each contribute to the outcome of invasion. For instance, in the case of a plant pathogen, the structure of the resource competition network was a better predictor of invasion outcome than biodiversity (Wei *et␣al*, 2015). In multiple invasions of microbial communities, the dispersal factor of the initial invader abundance (i.e., propagule pressure) was found to be the key determinant of the outcome of invasion (Acosta *et␣al*, 2015; Ketola *et␣al*, 2017; Kinnunen *et␣al*, 2018).

Synthetic communities composed of known organisms can be used to investigate the driving factors of invasion outcome (Wei *et␣al*, 2015; Ketola *et␣al*, 2017). Synthetic communities enable control of initial inoculum (i.e., organism presence/absence and initial abundance), which can be manipulated to understand the ecological and molecular mechanisms influencing invader growth. Dynamic computational models informed by the experimental measurements such as the generalized Lotka–Volterra (gLV) model can be used to decipher microbial interactions and predict community assembly (Mounier *et␣al*, 2008; Marino *et␣al*, 2014; Gonze *et␣al*, 2018). Previous modeling efforts with synthetic communities have revealed that pairwise interactions are informative of community assembly, making the characterization of lower‐order assemblages a powerful way to predict the behaviors of multispecies communities (Venturelli *et␣al*, 2018).

In this work, we use a defined synthetic gut community that represents the phylogenetic diversity of natural gut microbiota to study the determinants of *C. difficile* invasion success. To decipher microbial interactions and make predictions of community assembly and invasion, we use our data to construct a gLV model of our system and demonstrate that our model can accurately predict community assembly. Based on the inferred gLV interaction network, we demonstrate that negative interactions dominate the growth of *C. difficile*, which is a unique feature compared with all other species in our system. We identify multiple mechanisms that contribute to the inhibition of *C. difficile* growth including resource competition and external pH modification, highlighting that the mechanisms of inhibition of *C. difficile* vary across community contexts. Guided by our model, we identify a key closely related species, *Clostridium hiranonis*, that inhibits *C. difficile* growth in different synthetic communities. To investigate the ecological factors influencing invasion, we study the effect of propagule pressure and species richness on *C. difficile* growth. Our results show that *C. difficile* abundance exhibits a strong inverse relationship with species richness across a wide range of community contexts, and that this relationship is not universal to all species in our community. While increasing the propagule pressure of *C. difficile* can increase its abundance in the community, the sensitivity of each community to propagule pressure and the maximum saturating abundance of *C. difficile* are dictated by the microbial interaction network. We show that microbial communities feature a wide range of resistances to *C. difficile* and multiple mechanisms of *C. difficile* inhibition. Therefore, information about ecological and molecular mechanisms could be exploited to design bacterial therapeutics to inhibit *C. difficile*.

## Results

### *C. difficile* coexists in coculture with a subset of gut microbes

**Figure 1:**
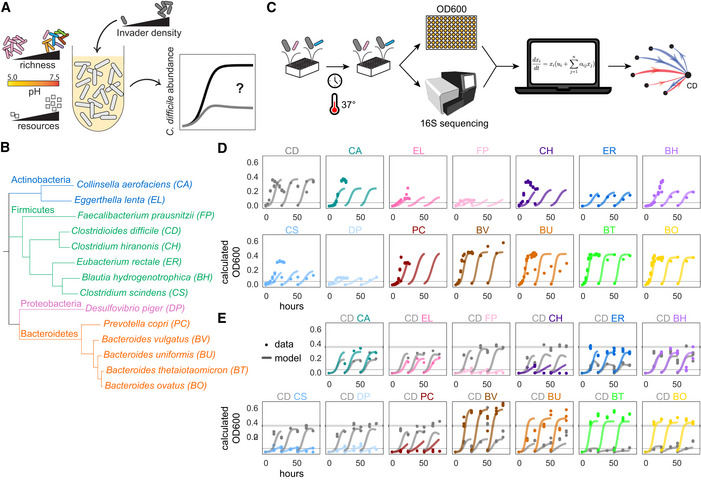
Investigating the ecological principles of *C. difficile* invasion using a diverse synthetic human gut community *C. difficile* (CD) invasibility is hypothesized to depend on initial invader density, species richness, environmental pH, and resource availability.Phylogenetic tree of 13‐member resident synthetic gut community and *C. difficile* based on concatenated alignment of 37 marker genes.Schematic of experimental and modeling workflow. Synthetic communities are cultured in microtiter plates in anaerobic conditions and incubated at 37°C. The absolute abundance of each species is determined by measuring cell density at 600 nm (OD600) and community composition using multiplexed 16S rRNA sequencing. Absolute abundance data are used to infer the parameters of a generalized Lotka–Volterra (gLV) model.Absolute abundance (OD600) of monospecies over time for three growth cycles. Datapoints indicate experimental biological replicates. Lines indicate simulations using the generalized Lotka–Volterra Full Model (trained on monospecies, pairs, and multispecies data, see Materials and Methods). Thin horizontal gray line indicates abundance threshold of 0.05 OD600.Absolute abundance (calculated OD600) of pairwise communities containing *C. difficile* over time for three growth cycles. Species were inoculated at an equal abundance ratio of *C. difficile* to resident species based on OD600 measurements. Datapoints indicate experimental data replicates. Lines indicate simulations using the generalized Lotka–Volterra Full Model (trained on monospecies, pairs, and multispecies data, see Materials and Methods). Thin horizontal gray line indicates abundance threshold of 0.05 OD600. Thick horizontal gray line indicates *C. difficile* monospecies maximal OD600 of 0.36. Calculated OD600 is the product of 16S relative abundance and community OD600. Data information: In D and E, *n* = 1–3 biological replicates (See Appendix␣Table S4 for replicate information of each condition).

We sought to understand the ecological principles of *C. difficile* invasion using synthetic gut communities (Fig 1A). As a representative community, we chose a consortium of 13 prevalent gut microbes spanning the major human gut phyla *Bacteroidetes*, *Firmicutes*, *Actinobacteria*, *and Proteobacteria* (Forster *et␣al*, 2019). The community features *Clostridium scindens*, a species previously shown to inhibit growth of *C. difficile* in gnotobiotic mice (Buffie *et␣al*, 2014), and a well‐characterized set of 12 diverse species whose interactions on community assembly have been previously studied and computationally modeled (Venturelli *et␣al*, 2018) (Fig 1B).

We used this synthetic gut community to investigate interspecies interactions influencing *C. difficile* growth. To decipher interspecies interactions driving *C. difficile* growth, we assembled combinations of species in microtiter plates in an anaerobic chamber and measured cell density by absorbance at 600 nm (OD600) and community composition by 16S rRNA gene sequencing at time points of interest (Materials and Methods). The absolute abundance of each species was calculated by multiplying its relative abundance from 16S rRNA gene sequencing by OD600 (“calculated OD600”). Time‐series measurements of species absolute abundance were used to infer the parameters of the gLV model (Fig 1C). The gLV model is a system of coupled ordinary differential equations that captures the growth rate and intra‐species interactions of single‐species and interspecies interactions that modify the growth dynamics of each species. The gLV model can be used to decipher interspecies interactions and predict the dynamics of all possible subcommunities within a larger system (Venturelli *et␣al*, 2018; Clark *et␣al*, 2021) and thus can be used to study the interspecies interactions between *C. difficile* and the resident gut community (i.e., all species excluding *C. difficile*).

We first characterized the temporal behavior of pairwise communities of *C. difficile* with each resident gut bacteria since we hypothesized that these direct interactions would have the largest impact on *C. difficile* growth compared with the interactions between resident gut bacteria. To this end, each resident species was grown alone and in coculture with *C. difficile*, specifically the R20291 reference strain of the epidemic ribotype 027 (Exp1, Fig 1D and E). A summary of all experiments throughout this work can be found in Appendix␣Table S1. Since variation in initial species proportions have been shown to influence community assembly (Wright & Vetsigian, 2016; Venturelli *et␣al*, 2018), we inoculated the pairs at 1:1 and 1:9 ratios of *C. difficile* to resident species based on OD600 values (Fig 1E, Appendix␣Fig S1). The communities were passaged using a 1:20 dilution at 26 and 52 h to observe community assembly over three batch culture growth cycles to understand the longer‐term behavior of the consortia.

Over this period of time, *C. difficile* and the resident species coexisted (both species present at greater than 0.05 OD600 after 78 h) in 19 of 33 (56%) conditions of 1:1 initial ratio, and 15 of 31 (48%) conditions of 1:9 initial ratio (Fig 1E, Appendix␣Fig S1). Although *C. difficile* and *Bacteroides* species coexisted in coculture over this period, the abundance of *C. difficile* was reduced compared with its abundance in monospecies. *Bacteroides thetaiotaomicron* and *Bacteroides ovatus* strongly inhibited *C. difficile*, reducing *C. difficile*’s carrying capacity in the final growth passage to 17 and 42% of its monospecies carrying capacity, while *Bacteroides uniformis* and *Bacteroides vulgatus* moderately inhibited *C. difficile*’s carrying capacity to 72 and 73% of its monospecies carrying capacity (Fig 1D and E). *Bacteroides* species have been shown to inhibit *C. difficile* growth (Mullish *et␣al*, 2019; Ghimire *et␣al*, 2020; Pereira *et␣al*, 2020) via suggested mechanisms of competition for mucosal carbohydrates or toxicity due to secondary bile acids (Mullish *et␣al*, 2019; Pereira *et␣al*, 2020). Because our media does not contain mucins or bile acids, the observed inhibition indicates a separate inhibition mechanism of *C. difficile* by *Bacteroides* species. We also identified closely related species that inhibit *C. difficile* including *Clostridium hiranonis*, the closest relative to *C. difficile* in the system (Fig 1B), which reduced *C. difficile* carrying capacity in the first growth passage to 59% of its monospecies carrying capacity (in the second and third passages, *C. hiranonis* became extinct and the inhibition was relieved). The next closest relative, *Eubacterium rectale*, reduced *C. difficile’s* carrying capacity in the third growth passage to 38% of its monospecies carrying capacity (Fig 1D and E). In sum, these data show that *C. difficile* can coexist over multiple batch culture cycles with a subset of species in our community.

### Abundance of *C. difficile* in multispecies communities is inversely related to species richness

We next sought to understand whether the growth inhibition of *C. difficile* observed in a subset of pairwise communities persisted in multispecies communities and to identify the ecological principles governing *C. difficile*’s growth in multispecies communities. In order to design multispecies communities to experimentally characterize, we created a gLV model of our system trained on our monospecies data (Fig 1D), pairs data (Fig 1E), and previously published data of resident species pairs (Venturelli *et␣al*, 2018). We inferred an initial set of parameters of the gLV model (“Preliminary Model”, Appendix␣Fig S2A, Dataset EV1) based on these data (Table 1) and used the model to predict the abundance of *C. difficile* at 48 h in all possible 2–13 member resident communities (8,178 total communities, Appendix␣Fig S2B). Using the predictions from the Preliminary Model, we selected a set of 94 2–13 member communities whose *C*. *difficile* abundance spanned the full range of predicted *C. difficile* abundances and featured approximately equal representation of species at various initial species richness (number of species in the resident community).

**Table 1:** Data used for gLV models.

Model	Data
Preliminary Model	Exp1 Exp2 Pairwise communities from Venturelli *et al* (2018)
Full Model	Exp1 Exp2 Exp3 Exp5 Exp6 (except for MS002, MS010, MS011 data) Exp8

We experimentally assembled these communities with an equal initial abundance of all species (including *C. difficile*) and measured the composition of communities after 48 h (Exp2). We added *C. difficile* to communities at 0 h to investigate interspecies interactions in a perturbed, low‐density environment that could mimic a disturbance such as antibiotic treatment. We measured the community composition at 48 h as the Preliminary Model predicted that the majority of communities had reached stationary phase by this time. It is possible that the measured community compositions after this single batch culture cycle (short‐term dynamics) could differ from the composition of the communities after multiple dilution cycles (long‐term dynamics). However, measuring the community composition at the end of a single batch culture cycle allowed us to investigate the ecological and molecular factors influencing *C. difficile* growth in a wide range of community contexts that varied in the presence/absence of species and species richness levels.

**Figure 2:**
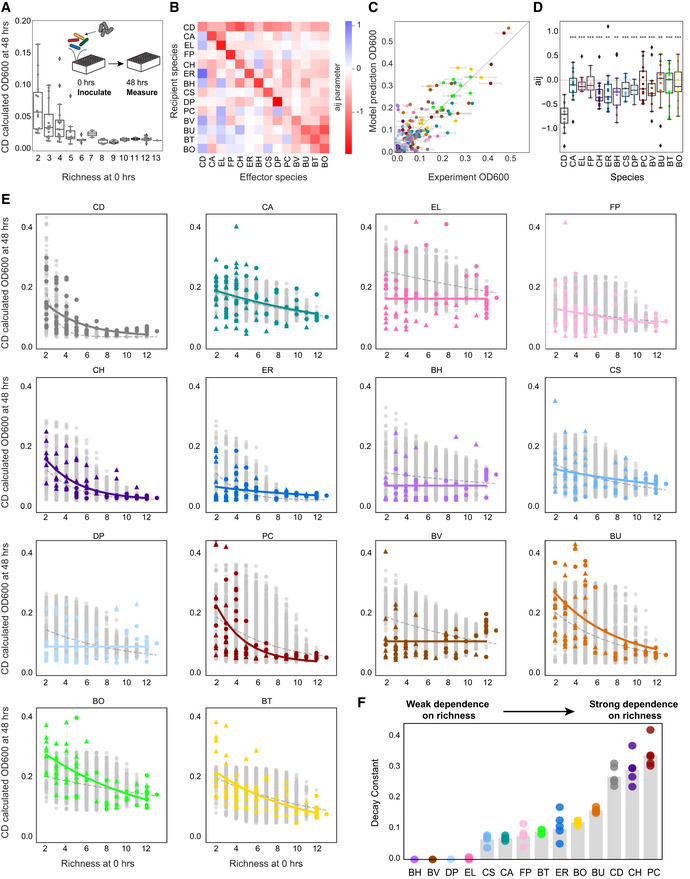
Growth of *C. difficile* decreases with community richness Swarmplot of *C. difficile* (CD) absolute abundance (calculated OD600) at 48 h in 94 subcommunities as a function of initial species richness. Datapoints indicate mean of biological replicates. Line represents median, box edges represent first and third quartiles, and whiskers indicate the minimum and maximum. Outliers are denoted by diamonds. Calculated OD600 is the product of 16S relative abundance and community OD600.Heatmap of interspecies interaction coefficients of the generalized Lotka–Volterra model (gLV) Full Model.Scatterplot of absolute abundance (calculated OD600) versus predicted absolute abundance by the gLV Full Model in 24 held‐out communities (Pearson *r* = 0.84, *P* = 6*10^−52^). Error bars represent one SD from the mean of biological replicates. Datapoint color indicates species identity. Gray line indicates y = x, or 100% prediction accuracy. Calculated OD600 is the product of 16S relative abundance and community OD600.Box␣plot of incoming interspecies interactions for each species in gLV Full Model. Stars represent statistical significance between *C. difficile* and each resident species: *P < 0.05, **P < 0.01, ***P < 0.001 according to an unpaired *t*‐test. Line represents median, box edges represent first and third quartiles, and whiskers indicate the minimum and maximum. Outliers are denoted by diamonds.Subplot of the absolute abundance of each species at 48 h as a function of initial species richness in all 16,370 possible subcommunities of 2–13 species simulated by the gLV Full Model (gray data points) and in 204 experimentally measured subcommunities (mean value of biological replicates, colored data points). Circles represent subcommunities included in Full Model training dataset. Triangles represent subcommunities not included in Full Model training dataset. Lines display exponential decay model (y = a*e* ^−bx^) fit to simulated data (gray dashed line) and experimental data (solid colored line). Calculated OD600 is the product of 16S relative abundance and community OD600.Barplot of decay constants b from exponential decay fit to experimental data in E. Colored datapoints are best fit parameters from five models, where each model was trained on a randomly sampled subset of the data consisting of 4/5 of the experimental datapoints. Gray bar indicates best fit parameter value from model trained on all data. Data information: In A, C, and E, *n* = 1–3 biological replicates.

We first looked at the relationship between initial species richness and *C. difficile* abundance in this dataset. The biodiversity–invasibility hypothesis holds that species‐rich communities have a higher fraction of ecological niches occupied, which reduces the availability of niches for invader species and thus enhances resistance to invasion relative to low‐richness communities (Elton, 1958). In agreement with the ecological theory, the mean *C. difficile* abundance across different communities decreased with species richness (Fig 2A). The negative relationship between species richness and *C. difficile* abundance remained the same whether richness was evaluated at the initial or final time point (Fig 2A, Appendix␣Fig S3A). Notably, *C. difficile* did not establish in any communities with richness greater than eight. The full community (13 resident members) excluded *C. difficile* from the community by 48 h. This resistance of the full community was observed not only with the ribotype 027 strain, but also for three individual clinical isolates of *C. difficile* that originated from patients within 72 h of their *Clostridioides difficile* Infection (CDI) diagnosis (Watson *et␣al*, 2019) (Exp6, Appendix␣Fig S3B, Materials and Methods).

**Figure EV1:**
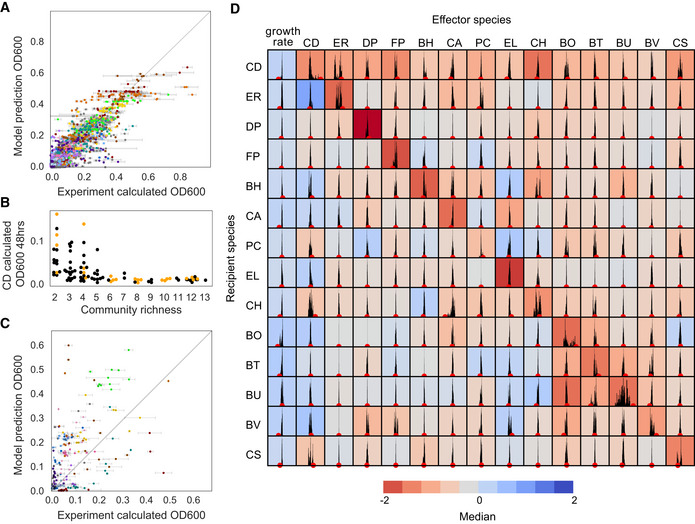
Analysis of parameter uncertainty and predictive capability of generalized Lotka–Volterra models Scatterplot of goodness of fit of experimental absolute abundance (calculated OD600) versus simulated species absolute abundance using the Full Model for the communities in the training data set (Pearson *r* = 0.89, *P* = 0.0). Error bars represent one SD from the mean of biological replicates. Gray line indicates y = x, or 100% prediction accuracy. Calculated OD600 is the product of 16S relative abundance and community OD600.Swarmplot highlighting 24 communities chosen as the held‐out set (data also shown in Fig 2A). Orange datapoints represent held‐out communities from training set. Black datapoints indicate communities from Fig 2A in training data set. Calculated OD600 is the product of 16S relative abundance and community OD600.Scatterplot of experimental absolute abundance (calculated OD600) versus predicted species absolute abundance (OD600) using the Preliminary Model for the 24 held‐out communities (Pearson *r* = 0.52, *P* = 1*10^−14^). Error bars represent one SD from the mean of biological replicates. Gray line indicates y = x, or 100% prediction accuracy. Calculated OD600 is the product of 16S relative abundance and community OD600.Histograms of parameter values determined using Metropolis–Hastings Monte Carlo (MCMC) analysis. Red dots indicate the parameter value in the Full Model (Materials and Methods). The black histograms indicate the MCMC distribution. The x‐axis is scaled to median of MCMC distribution +/− 0.25. The color of each subplot denotes the median value of the MCMC distribution. Data information: In A‐C, *n* = 1–3 biological replicates.

We wanted to understand whether the strong inverse relationship between species richness and *C. difficile* abundance could be explained by its interactions with the community. To investigate this question, we inferred a new set of gLV model parameters (“Full Model”, Fig 2B, Dataset EV2) using measurements of monospecies, pairwise and multispecies consortia (Table 1) and found that the Full Model had a high goodness of fit to the training data (Fig EV1A, Pearson *r* = 0.89, *P* = 0.0). To validate the predictive capability of the Full Model, we held out 24 randomly sampled communities from the training data set that spanned a broad range of species richness and *C. difficile* abundance (Fig EV1B) and found that the model predicted the community composition of the held‐out dataset with high accuracy (Fig 2C, Pearson *r* = 0.84, *P* = 6*10^−52^). In contrast, the Preliminary Model trained on monospecies and pairs was substantially less predictive of these 24 multispecies communities, indicating that the model required information from the multispecies experiments (Fig EV1C, Pearson *r* = 0.52, *P* = 1*10^−14^). We performed parameter uncertainty analysis to determine whether the parameters were sufficiently constrained by the data using Metropolis–Hastings Markov chain Monte Carlo (MCMC) (Materials and Methods). The coefficient of variation (CV) of 82% of the parameters was < 0.05 (CV ranged from 0.006 to 0.06), indicating that the parameters were sufficiently constrained by the data (Fig EV1D).

Strikingly, in the interspecies interaction network (Fig 2B), all species inhibited *C. difficile. C. difficile* positively impacted most species in the community, which combines with the negative incoming interactions to generate multiple negative feedback loops on the growth of *C. difficile*. Increasing species richness increases the number of negative feedback loops on *C. difficile*’s growth, providing insight into the negative relationship between *C. difficile* abundance and species richness. *C*. *difficile* is unique in its large number and magnitude of incoming negative interactions in the system (Fig 2D). Because of this, we hypothesized that other species may not display the same strong inverse relationship between abundance in communities and richness. For example, a species with many positive incoming interactions may have a growth benefit in high richness communities containing growth‐promoting species.

**Figure EV2:**
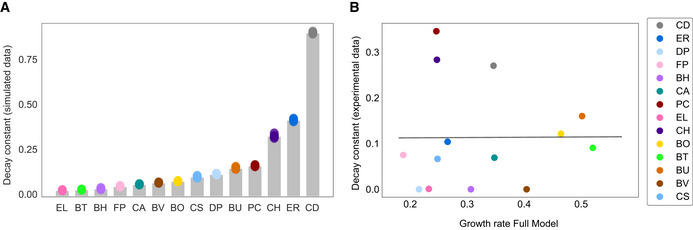
Analysis of simulated and measured decay constants quantifying the relationship between species richness and abundance Barplot of decay constants b from exponential decay fit to simulated data in 2e. Colored datapoints are best fit parameters from five models, where each model was trained on a subsample of the data consisting of 4/5 of the experimental datapoints. Gray bar indicates best fit parameter from model trained on all data.Scatterplot of relationship between species growth rate in the Full Model and decay constant fit to experimental data shown in Fig 2F. Gray line indicates linear regression (y = 0.006x+0.111, Pearson *r* = 0.01, *P* = 0.98).

We analyzed the relationship between abundance and richness for each species in multispecies communities with and without *C. difficile* (Exp2 and Exp3, Fig 2E). Additionally, we used the Full Model to simulate the abundance of each species in all possible communities (16,383 total communities) to supplement our experimental data (Fig 2E, gray points). To quantify the relationship between abundance and richness for each species, we fit an exponential decay model (y = a*e* ^−bx^) to the data (solid colored lines) and simulations (dashed gray lines). The decay constant of the experimental data fits reveals that *C. difficile*, *C. hiranonis*, and *Prevotella copri* have strong negative relationships between abundance and species richness (*b* > 0.2, Fig 2F). However, *Blautia hydrogenotrophica*, *B. vulgatus*, *Desulfovibrio piger*, and *Eggerthella lenta* displayed no relationship between abundance and richness (b < 0.05). The decay constants fit to the simulated data (Fig EV2A) showed agreement with those fit to the experimental data (Fig 2F). For example, in the model, *C. difficile* and *C. hiranonis* were strongly dependent on richness (b > 0.2) and *E. lenta* and *B. hydrogenotrophica* displayed no relationship (b < 0.05). The decay constants did not correlate with species growth rate, so the relationship between abundance and richness cannot be explained by whether a species was fast‐ or slow‐growing (Fig EV2B). *C. hiranonis* also features a large number of negative incoming interactions, suggesting that the mechanism of *C. hiranonis*’s dependence on richness could be similar to that of *C. difficile* (Fig 2D). Overall, our data and model analysis shows that the abundance of *C. difficile* has a strong inverse relationship with species richness and this relationship is not universal to all species.

### Increasing propagule pressure increases *C. difficile* abundance in synthetic communities

**Figure 3:**
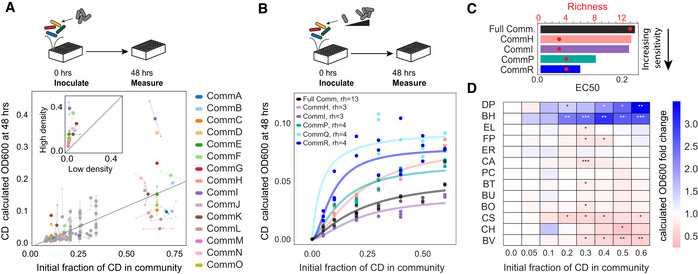
Impact of initial density on the growth of *C. difficile* Scatterplot of *C. difficile* (CD) absolute abundance (calculated OD600) at 48 h in communities as a function of the initial fraction of *C. difficile*. *C. difficile* was introduced into the communities at 0 h. Gray data points are 2–13 member resident communities measured in Fig 2A. Colored data points are 3–4 member communities measured at two initial conditions: low density (approximately 10% of total community OD600) or high density (approximately 65% total community OD600). Gray line indicates a linear regression (y = 0.25x‐0.01, Pearson *r* = 0.75, *P* = 1*10^−23^). Transparent data points indicate biological replicates and are connected to the corresponding mean values by transparent lines. Inset: Abundance of *C. difficile* at 48 h in communities invaded with low density or high density. Gray y = x line indicates no change in abundance. Calculated OD600 is the product of 16S relative abundance and community OD600.Absolute abundance (ODO600) of *C. difficile* at 48 h as a function of the initial fraction of *C. difficile* in different synthetic communities. *C. difficile* was added to communities at 0 h. Datapoints indicate biological replicates. Lines indicate Hill function fits (Materials and Methods). Resident species richness (rh) at 0 h is indicated in legend. Calculated OD600 is the product of 16S relative abundance and community OD600.Initial fraction of *C. difficile* corresponding to the half‐maximum abundance (EC50) inferred based on the fitted Hill functions in B for a subset of communities with sufficient measurements to constrain the function parameters. Red circles indicate the resident species richness at 0 h.Heatmap of the fold change of species absolute abundance (mean value of biological replicates, *n* = 3) in the full community with 5–60% initial *C. difficile* compared to 0% initial *C. difficile* condition. Calculated OD600 is the product of 16S relative abundance and community OD600. Stars represent statistical significance: *P < 0.05, **P < 0.01, ***P < 0.001 according to an unpaired *t*‐test. Data information: In A, *n* = 1–3 biological replicates. In B, *n* = 1–3 biological replicates (see Appendix␣Table S4 for replication information of each condition). In D, *n* = 3 biological replicates.

**Figure EV3:**
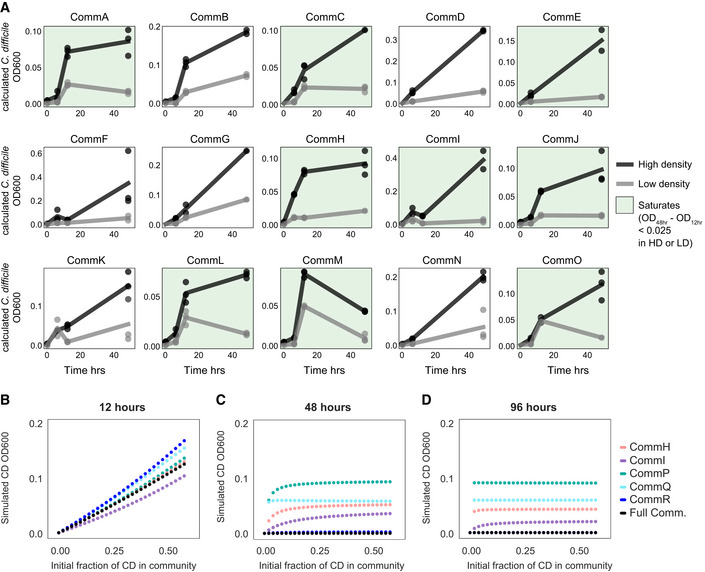
Dependence of *C. difficile* abundance on propagule pressure over time ALineplots of *C. difficile* (CD) abundance over time in CommA‐CommO communities. Final timepoint is same as shown in Fig 3A. In low‐density conditions, *C. difficile* inoculated at 10% of total community OD600 at 0 h. In high‐density conditions, *C. difficile* inoculated at 65% of total community OD600 at 0 h. Data points indicate biological replicates and lines indicate mean value of biological replicates. Communities where *C. difficile* abundance saturates by 48 h in either high‐density or low‐density conditions are highlighted in green (saturation defined as difference in *C. difficile* OD600 between 48 and 12 h is less than 0.025).B–DLineplots of predicted absolute abundance (OD600) of *C. difficile* at 12, 24, and 96 h as a function of the initial fraction of *C. difficile* as simulated by the Full Model. Data information: In A, *n* = 1–3 biological replicates (See Appendix␣Table S4 for replicate information of each condition).

The propagule–pressure hypothesis dictates that increasing propagule pressure, or the amount of invader (a product of its dispersal frequency and abundance), increases the chance of a successful invasion (Lockwood *et␣al*, 2005). To characterize the effect of propagule pressure in our system, we next looked at the relationship between the propagule pressure of *C. difficile* and its abundance at 48 h. In our system, we add *C. difficile* to the system a single timepoint, so the propagule pressure of *C. difficile* is only affected by initial abundance of *C. difficile*. In our experiments, we define propagule pressure as the initial fraction of *C. difficile*. We analyzed the relationship between initial fraction of *C. difficile* and final abundance of *C. difficile* in the 2–13 member multispecies communities (Exp2, gray data points in Fig 3A) in addition to measurements of 15 3–4 member resident communities (Exp4, Appendix␣Table S2). We focused on 3–4 member communities because communities in this narrow richness range featured a wide range of *C. difficile* abundances at 48 h (Fig 2A). We chose 15 communities with a wide range of predicted *C. difficile* abundances. We inoculated these communities at multiple species ratios and measured the composition over time (colored data points in Figs 3A and EV3A). In agreement with the theory, the final abundance of *C. difficile* correlated with the initial fraction of *C. difficile* in the community (Fig 3A, Pearson *r* = 0.75, *P* = 1*10^−23^). In all 15 3–4 member communities, the abundance of *C. difficile* at 48 h was higher in communities inoculated with a high initial fraction of *C. difficile* (approximately 65% of total community biomass) compared with a low initial fraction of *C. difficile* (approximately 10% of total community biomass) (Fig 3A, inset). This indicates that increasing propagule pressure of *C. difficile* can increase its abundance in the community at 48 h.

For a set of 3–4 member communities, we performed a similar experiment but increased the number of tested initial fractions to better resolve the relationship between propagule pressure and abundance at 48 h (Exp5, Fig 3B). Increasing the propagule pressure of *C. difficile* yielded higher *C. difficile* abundance in the assembled community within a given range. However, the abundance of *C. difficile* approached a similar maximum abundance for high initial fractions beyond a threshold. The abundance of *C. difficile* at saturation in the community varied in different subcommunities, suggesting that the microbial interaction network determined the maximum abundance of *C. difficile*. We defined the sensitivity to propagule pressure as the initial invader fraction that resulted in the half‐maximal abundance of the invader at 48 h, analogous to the EC50 of a dose–response curve (Fig 3C). The communities displayed different sensitivities to the initial fraction of *C. difficile*, with the EC50 ranging between 0.1–0.2. Community R (Appendix␣Table S2) was the most sensitive to invasion by *C. difficile*. Therefore, while increasing propagule pressure of *C. difficile* can increase its abundance to a maximum threshold, the sensitivity to propagule pressure and maximum saturating abundance of *C. difficile* are dictated by the microbial interaction network.

In the experiments and simulations, the total initial OD600 was held constant, resulting in lower initial OD600 of each species with increasing richness (Materials and Methods). Therefore, we considered the possibility that *C. difficile*’s low abundance in high richness communities (Fig 2A) could be a result of lower initial abundance. To test this possibility, we introduced *C. difficile* into the full community (richness of 13) at a range of initial fractions. We observed that *C. difficile* grew to a higher abundance in the full community when propagule pressure was increased, although the maximum abundance was lower than in the majority of 3–4 member communities (Fig 3B). Therefore, while increasing propagule pressure can partially overcome the inhibiting effect of species richness on *C. difficile* growth in the linear regime of the dose response, high richness still reduces the maximum saturating *C. difficile* abundance.

The variation in *C. difficile* abundance at 48 h with propagule pressures in our experiments could be a transient effect as a consequence of being further away from the long‐term composition of the assembled community or history‐dependent behavior that persists long term. Our time‐series data of 3–4 member communities indicated that the abundance of *C. difficile* approached saturation by 48 h in most of the communities (Figs 3A and EV3A). This suggests that inoculating communities with high or low density of *C. difficile* yielded distinct long‐term abundances in these communities. In contrast, stability analysis of our model (Materials and Methods) found that all subcommunities are monostable and therefore have no long‐term history dependence. For example, our model simulations for the six communities in Fig 3B predict that the strong dependence of *C. difficile* abundance on propagule pressure is transient, as the dependence at early times (12 h) is reduced at longer timescales and converges to a final abundance independent of propagule pressure since these communities are monostable (Fig EV3B–D). Therefore, the gLV model may be missing information about the long‐term history‐dependent behaviors of these communities.

When increasing the propagule pressure of *C. difficile* in the six resident communities in Fig 3B, the composition of the resident communities at 48 h varied with initial *C. difficile* abundance. To quantify this variation, we computed the normalized Euclidean distance between the resident community composition in the presence and absence of *C. difficile* (Materials and Methods). The Euclidean distance correlated with the abundance of *C. difficile* in the community in our experimental data (Appendix␣Fig S4A, Pearson’s *r* = 0.61, *P* = 6*10^−13^) as well as in simulations of all 1–13 member resident communities invaded with *C. difficile* 6 h after inoculation (Appendix␣Fig S4B, Pearson’s *r* = 0.58, *P* = 0.0). These data indicate that in general, higher abundance of *C. difficile* results in a larger impact on the composition of the resident community. However, the unexplained variation in Euclidean distance for a fixed *C. difficile* initial abundance suggests that interspecies interactions also impact the extent to which the resident community is altered by the presence of *C. difficile*.

**Figure EV4:**
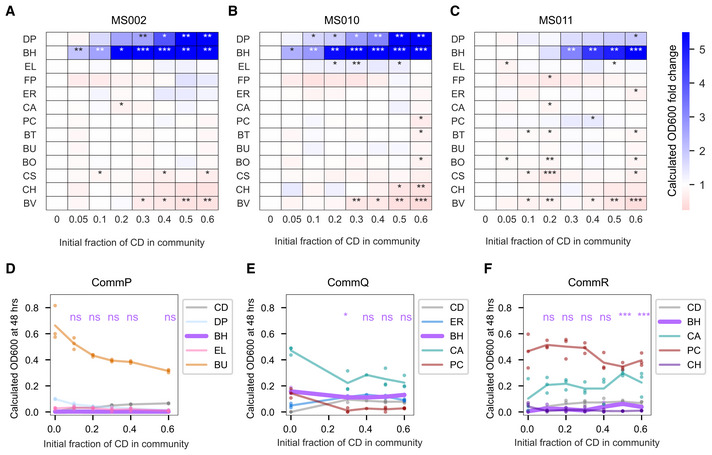
Impact of *C. difficile* on resident species abundances A–CHeatmap of the fold change of species absolute abundance (mean value of biological replicates) in full community with 5–60% initial *C. difficile* (CD) compared to the 0% initial *C. difficile* condition. Stars represent statistical significance: *P < 0.05, **P < 0.01, ***P < 0.001 according to an unpaired *t*‐test. A: *C*. *difficile* strain MS002, B: *C*. *difficile* strain MS010, C: *C*. *difficile* strain MS011.D–FLineplots of species absolute abundance (calculated OD600) at 48 h as a function of initial *C. difficile* fraction. Datapoints indicate biological replicates and lines indicate the mean. Calculated OD600 is the product of 16S relative abundance and community OD600. Stars indicate a statistically significant difference in the absolute abundance of *B. hydrogenotrophica* compared to the absolute abundance of *B. hydrogenotrophica* in 0% initial *C. difficile* condition: *P < 0.05, **P < 0.01, ***P < 0.001, ns = no significant difference according to an unpaired *t*‐test. D: CommP, E: CommQ, F: CommR. Data information: In A‐C, D, F *n* = 3 biological replicates. In E, *n* = 1 or *n* = 3 biological replicates (see Appendix␣Table S4 for replicate information of each condition).

In the full community, we observed that the abundance of *D. piger* and *B. hydrogenotrophica* significantly increased in communities with higher *C. difficile*, while the abundance of *B. vulgatus* significantly decreased (Fig 3D). These trends were observed in the full community with the ribotype 027 strain of *C. difficile* as well as the full community with individual clinical *C. difficile* isolates (Fig EV4A). The interaction network from our model (Fig 2B) features a positive interaction between *C. difficile* and *B. hydrogenotrophica*, suggesting that increasing initial *C. difficile* abundance directly promotes the growth of *B. hydrogenotrophica*. However, the interspecies interaction coefficients impacting *D. piger* and *B. vulgatus* were not consistent with the observed trends with these two species. These data suggest that the gLV model may not capture the effects of high initial *C. difficile* density on the growth of all resident gut species. While at high initial densities *C. difficile* significantly increased the abundance of␣*B. hydrogenotrophica* in the full community (Fig 3D), *B. hydrogenotrophica* abundance was not affected in the 3‐member communities F, G, and N (Fig EV4B), highlighting that *C. difficile*’s impact on a given species depends on the community context in addition to its initial abundance. We note that *B. hydrogenotrophica* and *D. piger* share a similar metabolic niche as hydrogen consumers (Bernalier *et␣al*, 1996; Loubinoux *et␣al*, 2002), suggesting *C. difficile* could enhance their growth through a shared mechanism.

### A subset of synthetic communities inhibits *C. difficile* via acidification of the environment

**Figure 4:**
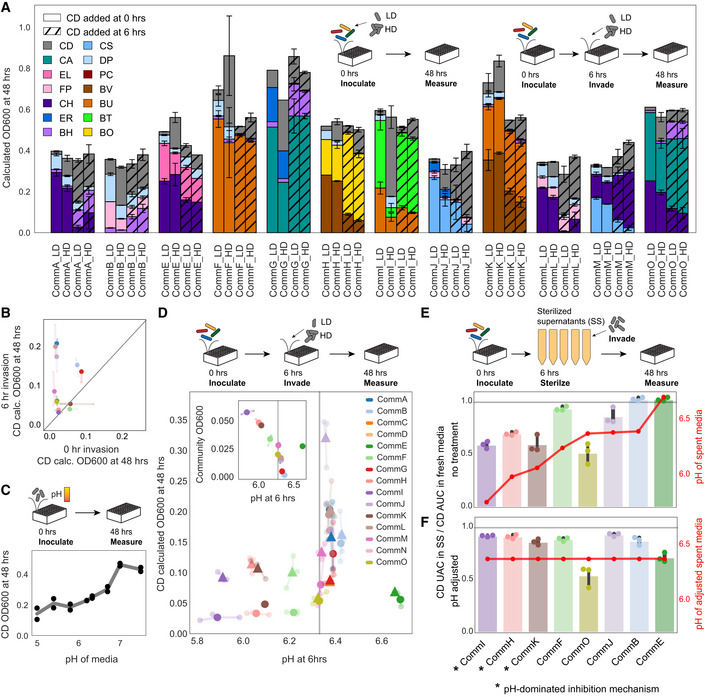
Impact of environmental factors on *C. difficile* invasion ABarplot of composition of communities invaded with *C. difficile* (CD) at low density (“LD”) or high density (“HD”). Color indicates species identity. Hash indicates invasion time. Error bars represent one SD from the mean of biological replicates. Calculated OD600 is the product of 16S relative abundance and community OD600.BScatterplot of the absolute abundance (calculated OD600) of *C. difficile* at 48 h in communities when introduced at 0 h versus 6 h at low density (approximately 10% community OD600). Transparent data points indicate biological replicates and are connected to the corresponding mean values by transparent lines. Line denotes the x = y line corresponding to no change in growth. Color indicates community, see legend in D. Calculated OD600 is the product of 16S relative abundance and community OD600.CLineplot of *C. difficile* OD600 at 48 h as a function of the initial environmental pH. Datapoints indicate biological replicates and line indicates mean value.DScatterplot of the absolute abundance (calculated OD600) of *C. difficile* at 48 h in invaded communities as a function of the environmental pH at time of invasion. Fifteen 3–4 member communities were invaded with (▲) high‐density *C*. *difficile* (approximately 33% community OD600) or (●) low‐density *C. difficile* (approximately 10% community OD600) at 6 h. Color indicates community. Vertical gray line indicates pH of fresh media. Inset: Scatterplot of environmental pH and total community OD600 at 6 h. Transparent data points indicate biological replicates and are connected to the corresponding mean values by transparent lines. Vertical gray line indicates environmental pH of fresh media. Calculated OD600 is the product of 16S relative abundance and community OD600.E,FBar plot of fold change of *C. difficile* growth in sterilized supernatants (E) or supernatants where the pH was adjusted to the pH of fresh media (F) compared to the growth of *C*. *difficile* in fresh media. Growth was quantified as area under the curve (AUC) of OD600 from 0 to 20 h. Datapoints indicate biological replicates, bars indicate mean value, and error bars represent one SD from the mean of biological replicates. Red line shows pH of community supernatants collected at 6 h (top) and pH adjusted supernatants (bottom). Horizontal gray line indicates no change in growth compared to fresh media. Data information: In A, B, and D, *n* = 1–3 biological replicates (see Appendix␣Table S4 for replication information of each condition). In C, *n* = 2 biological replicates. In E and F, *n* = 3 biological replicates.

While the community experiments revealed the importance of species richness and propagule pressure on the establishment of *C. difficile* in multispecies communities, there remains unexplained variation in the data. For example, communities with the same richness invaded with equal abundances of *C. difficile* showed a wide range of *C. difficile* abundances at 48 h (Fig 2A). Since environmental pH has been shown to influence *C. difficile*’s growth in previous studies (Wetzel & McBride, 2020; Yuille *et␣al*, 2020), we turned next to investigate how biotic modification of the environment alters the growth of *C. difficile*. To this end, we grew the set of 15 3–4 member communities for 6 h before invading with low or high initial densities of *C. difficile* to give the communities time to modify the environment. At the time of invasion, we measured the composition of the resident community and the pH of the media (Exp6, Fig 4A). To understand the role of invasion timing on the growth of *C. difficile*, we compared the *C. difficile* abundance in these communities invaded at 6 h with communities invaded at 0 h (Exp5) (Fig 4B). *C.␣difficile*’s ability to establish in multiple communities significantly depended on the timing of introduction (Fig 4B), indicating that biotic modification of the environment during those 6 h altered *C. difficile*’s ability to grow.

Communities that lowered the pH of the media during the first 6 h featured lower *C. difficile* abundance (Fig 4D). However, communities with lower pH at the time of invasion also had higher total biomass (Fig 4D, inset). Since these variables are related due to growth‐coupled production of acidic fermentation end products, either pH or resource competition could be responsible for inhibition of *C. difficile*. Because *C. difficile* abundance increased with environmental pH (Fig 4C), we hypothesized that growth inhibition was due to changes in media pH. To test our hypothesis, we grew a set of the communities and harvested and sterilized the community supernatants after 6 h. We grew *C. difficile* in either the sterile supernatant (Fig 4E) or a modified sterile supernatant wherein the pH was adjusted to the pH of the fresh media to eliminate the impact of pH on growth (Fig 4F). We quantified the growth of *C. difficile* as the area under the curve (AUC) of *C. difficile* OD600 over 20 h, which is influenced by both growth rate and carrying capacity. In Communities H, I, and K, which strongly inhibit *C. difficile* in both coculture and supernatant, increasing the supernatant pH to the pH value of fresh media eliminated the inhibition of *C. difficile* (Fig 4F), indicating that pH was the driving factor of *C. difficile* inhibition in these community supernatants. Each of these communities contained an abundant *Bacteroides* species (Appendix␣Table S2) whose fermentation end products acidify the media, suggesting *Bacteroides* species are responsible for the inhibition via pH modification. We observed the pH‐dependent inhibition of *C. difficile* by CommI in several other medias with varying buffering capacity and available substrates, indicating this result is not specific to our media (Appendix␣Fig S5).

In contrast to this pH‐dependent inhibition, the sterile supernatant of Community O (CommO) composed of *C. hiranonis*, *Collinsella aerofaciens*, and *B*. *hydrogenotrophica*, whose pH did not significantly differ from the pH of fresh media, inhibited *C. difficile* regardless of pH adjustment (Fig 4E and F). This implies that this community inhibits *C. difficile* via a pH‐independent mechanism. *C. difficile* was not inhibited by the sterile supernatant of Community E (CommE) composed of *C. hiranonis*, *D*. *piger*, and *E*. *lenta*, which uniquely had a higher pH than fresh media (Fig 4E). However, *C. difficile*’s growth was inhibited when the pH of the CommE sterile supernatant was reduced to the pH of fresh media (Fig 4F). This suggests that the sterile supernatant promotes *C. difficile*’s growth by enhancing environmental pH and the community inhibits *C. difficile*’s growth by a separate pH‐independent mechanism. The growth inhibition by CommE was only revealed when the pH increase of the media was eliminated, demonstrating an interplay of different mechanisms influencing *C. difficile* growth within the same community.

**Figure EV5:**
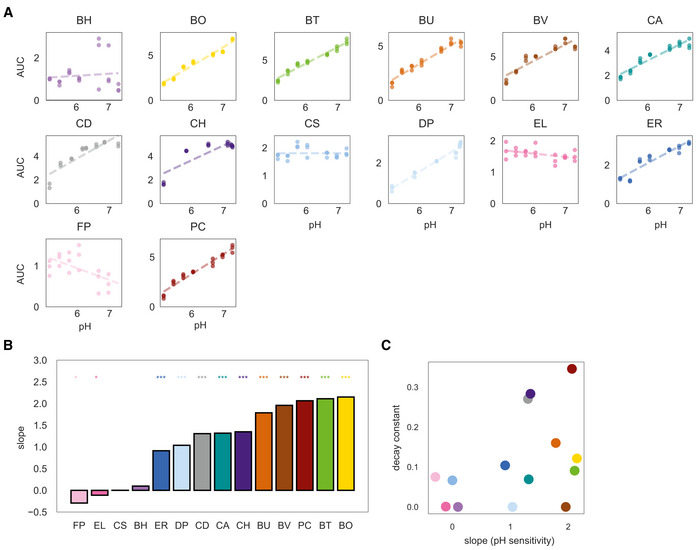
Resident gut species pH sensitivity in monoculture Lineplots of monospecies growth as a function of the initial environmental pH of adjusted fresh media. Growth is quantified as area under the curve (AUC) from 0 to 20 h. Datapoints indicate biological replicates and dashed lines indicate linear regression fits.Barplot of slopes of linear regression fit to data in A. Stars denote statistical significance: *P < 0.05, **P < 0.01, ***P < 0.001 according to an unpaired *t*‐test.Scatterplot of decay constants from Fig 2F as a function of pH sensitivity slopes in B. Linear regression y = 0.06x+0.05, Pearson *r* = 0.44, *P* = 0.11). Data information: In A, *n* = 2–3 biological replicates (See Appendix␣Table S4 for replicate information of each condition).

We explored if the sensitivity of a species to environmental pH correlated with its dependence on species richness as determined in Fig 2E. We measured the AUC of each species as a function of initial environmental pH in monospecies and determined the slope of the line fit to these data (Fig EV5A), representing the sensitivity of species growth to external pH. Our results demonstrated that there is no significant correlation between sensitivity to species richness and sensitivity to pH (Fig EV5C). Therefore, while acidification of the media is one mechanism by which communities can inhibit *C. difficile* in our system, our results suggest that there are also pH‐independent mechanisms that underlie *C. difficile*'s strong inverse relationship between abundance and species richness.

### *C*. *hiranonis* inhibits *C*. *difficile* through a pH‐independent mechanism

**Figure 5:**
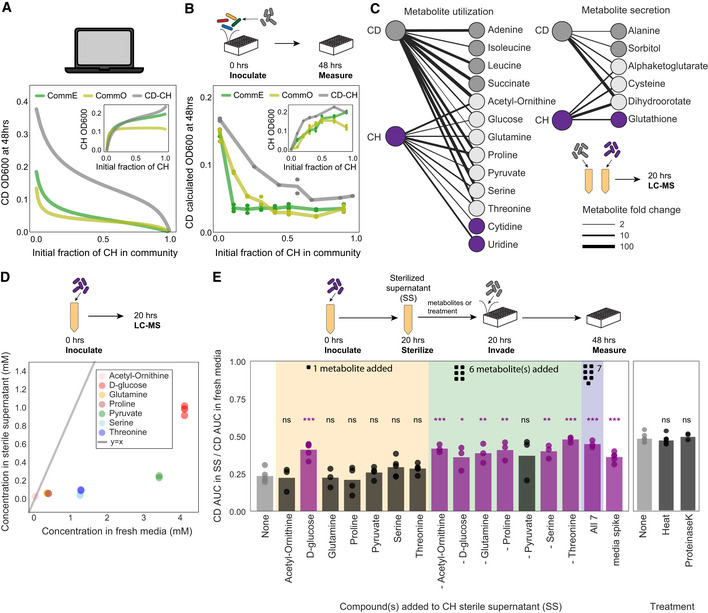
*C. hiranonis* inhibits the growth of *C. difficile* Lineplot of simulated *C. difficile* (CD) absolute abundance (OD600) at 48 h using the generalized Lotka–Volterra (gLV) Full Model as a function of the initial fraction of *C. hiranonis* (CH) in different communities. Inset: Lineplot of simulated *C. hiranonis* absolute abundance (OD600) at 48 h in the gLV Full Model as a function of initial fraction of *C. hiranonis* in the community.Lineplot of *C. difficile* absolute abundance (calculated OD600) at 48 h as a function of the initial fraction of *C. hiranonis* in the community. Inset: Lineplot of *C. hiranonis* absolute abundance (calculated OD600) at 48 h in community as a function of initial fraction of *C. hiranonis* in the community. Datapoints indicate biological replicates, and lines indicate mean values. Calculated OD600 is the product of 16S relative abundance and community OD600.Bipartite network of metabolite utilization and secretion by *C. difficile* and *C. hiranonis* monospecies after 20 h of growth. Metabolites that changed by at least twofold compared to media control are shown. Edge width is proportional to the metabolite fold change.Scatterplot of concentration of seven metabolites in fresh media compared to *C. hiranonis* sterile supernatant.Bar plot of fold change of *C. difficile* growth in sterilized supernatants compared to the growth of *C. difficile* in fresh media. Growth was quantified as the area under the curve (AUC) of OD600 from 0 to 20 h. Datapoints indicate biological replicates, and bars indicate mean value. Media spike condition is the addition of 1X fresh media concentrated in a small volume. Stars represent statistical significance between condition and unmodified *C. hiranonis* supernatant: *P < 0.05, **P < 0.01, ***P < 0.001, and ns= not significant, according to an unpaired *t*‐test. Bars of all significant conditions are shaded purple. Data information: In C and D, *n* = 3 biological replicates. In B, *n* = 1–3 and in E, *n* = 3–5 biological replicates (see Appendix␣Table S4 for replication information of each condition).

We noted that the two communities that displayed pH‐independent growth inhibition (CommE and CommO) contained *C. hiranonis*, which has a strong bidirectional negative interaction with *C. difficile* in our Full Model (Fig 2B). Our model predicts that the abundance of *C. difficile* at 48 h decreases with increasing initial abundance of *C. hiranonis* in CommE, CommO, and the *C. difficile‐C. hiranonis* pair (Fig 5A). We confirmed this prediction experimentally (Exp7, Fig 5B). *C. difficile* was inhibited even by low initial amounts of *C. hiranonis*, featuring a significant decrease in growth between 0 and 10% initial *C. hiranonis* in CommE (> 4‐fold decrease) and CommO (> 1.5‐fold decrease) (Fig 5B). The strength of inhibition of *C. difficile* as a function of the *C. hiranonis* abundance was substantially higher in CommE and CommO than in the *C. hiranonis*‐*C. difficile* pair (Fig 5B). This result indicates that the other species in the communities enhanced the inhibitory effect of *C. hiranonis* on *C. difficile* growth.

We next considered the mechanism of *C. difficile* inhibition by *C. hiranonis*. *C. hiranonis* is known to convert primary bile acids into secondary bile acids which are inhibitory to *C. difficile* (Kitahara *et␣al*, 2001). However, with no primary bile acids in our media we turned to other possible inhibition mechanisms. *C. difficile* was inhibited by the sterile supernatants of CommE and CommO (Fig 4E, Appendix␣Fig S6), indicating the inhibition effect does not require direct cell contact. These mechanisms could include production of antibiotics or toxic metabolic byproducts, modification of pH, or competition over resources. *C. hiranonis* has been shown to consume a broad range of metabolites in our media conditions (more than any of the other species in our system) (Venturelli *et␣al*, 2018) and is known to contain genes for Stickland amino acid fermentations, a major process for energy generation in *C. difficile* (Ridlon *et␣al*, 2020). Therefore, *C. hiranonis* could inhibit *C. difficile* by utilizing multiple resources that *C. difficile* consumes. We performed exo‐metabolomics using liquid chromatography–mass spectrometry (LC‐MS) of *C. hiranonis* and *C. difficile* supernatants harvested at 20 h and found that the two species utilize an overlapping set of metabolites in our media (Fig 5C). We identified seven metabolites that significantly decreased (> 2‐fold) in both supernatants*:* acetyl‐ornithine, glucose, glutamine, proline, pyruvate, serine, and threonine.

We designed an experiment to provide insight into potential resource competition between *C. difficile* and *C. hiranonis* over these metabolites in coculture. We first determined the concentration of these metabolites in fresh media and in the supernatant of *C. hiranonis* at 20 h using LC‐MS with a standard curve of known concentrations of each of the seven metabolites (Fig 5D). We then adjusted the concentration of each of the seven metabolites in *C. hiranonis* sterile supernatant to match the concentration in fresh media. We grew *C. difficile* in these modified supernatants, as well as supernatants modified to adjust all combinations of six or seven of the metabolites. We quantified the growth of *C. difficile* as the AUC of *C. difficile* OD600 over 48 h (Fig 5E). The fold change between *C. difficile* growth in *C. hiranonis* supernatant compared to fresh media was 23%, displaying strong inhibition consistent with the inhibition observed in coculture (Fig 5B) and our interaction network (Fig 2B). *C. difficile* grew significantly better in glucose‐adjusted supernatant (41% of fresh media growth) compared to unmodified supernatant (23% of fresh media growth), but there was no significant difference for any of the other six individual metabolite adjustments. Replenishing all seven metabolites in the supernatant significantly increased *C. difficile*’s growth to a similar level as the glucose addition (45% of fresh media growth). Interestingly, there was no significant difference between *C. difficile* growth in the seven‐compound supernatant and the six‐compound supernatant excluding glucose. Therefore, the metabolites excluding glucose did not significantly impact *C. difficile* growth when added individually but they did as a set. The fact that *C. difficile* grew equally in all of the six‐compound supernatants suggests metabolic redundancy among this set of compounds. In sum, these data show that replenishing the seven metabolites can partially relieve the observed *C. hiranonis‐C. difficile* inhibition. This suggests that *C. difficile* is inhibited in *C. hiranonis* supernatant due to low concentration of some subset of these metabolites which were consumed by *C. hiranonis*. This is supported by our data that showed these metabolites are also consumed by *C. difficile* in monospecies. However, we cannot rule out that the addition of these metabolites could compensate for the effects of different compound(s) that inhibits *C. difficile*.

Because restoration of these compounds only partially relieved the inhibition by *C. hiranonis*, we were curious about the mechanism of the remaining inhibition. We considered the possibility that *C. hiranonis* and *C. difficile* compete over other resources in addition to the seven identified above. To test whether the restoration of other compounds could further relieve the inhibition, we supplemented concentrated media to the *C. hiranonis* supernatant (10X concentrated ABB spiked in a 1:10 dilution into *C. hiranonis* supernatant). After concentrated media supplementation, compounds that were completely consumed by *C. hiranonis* were at 1× of their original media concentration, and compounds not utilized by *C. hiranonis* were at 2× of their original media concentration. Therefore, if resource competition alone was responsible for the inhibition, the growth of *C. difficile* would be fully rescued in the supplemented condition to the level of growth in fresh media. However, in the supplemented supernatant, *C. difficile* exhibited 36% of its growth in fresh media (Fig 5E). Therefore, these data suggest that the inhibitory effect of *C. hiranonis* conditioned media is due to a combination of resource competition and a separate unknown inhibitory mechanism. Further, the pH of *C. hiranonis* supernatant was not significantly different from fresh media, eliminating pH as a potential mechanism. To test whether a protein was responsible for *C. difficile* inhibition, we inactivated proteins in the *C. hiranonis* supernatant by either denaturing with heat (95°C for 1 h) or digesting with the broad‐spectrum protease Proteinase K. Our results showed no significant difference in *C. difficile* growth in these two treatments compared to no‐treatment control, suggesting the inhibition by *C. hiranonis* is not due to proteinaceous antibiotics or toxins. In sum, our results suggest that *C. hiranonis* partially inhibited *C. difficile* via resource competition, but also inhibits *C. difficile* via a protein‐independent mechanism.

## Discussion

We combined bottom‐up construction of microbial communities with dynamic computational modeling to investigate microbial interactions impacting the growth of *C. difficile*. Our work demonstrates that microbial communities feature a wide range of resistances to *C. difficile* invasion. This variability in invasion outcome as a function of community context indicates that the choice of organisms is a major design factor that can be optimized to treat *C. difficile* infections and motivates exploiting information about ecological and molecular interactions in the design process. Previous efforts to design defined consortia for *C. difficile* inhibition used top‐down selections by reducing the complexity of cultured fecal samples alone or combined with screening of antibiotic resistance phenotypes (Lawley *et␣al*, 2012; Petrof *et␣al*, 2013). These methods rely on a trial‐and‐error approach to discover inhibitory consortia instead of testing consortia rationally designed to be inhibitory. Some consortia have been rationally designed by combining selected species in a bottom‐up approach, but we note that these selections use a single design criterion (Buffie *et␣al*, 2014; Pereira *et␣al*, 2020). We identified principles of *C. difficile* invasion that could be used as multiple criteria for designing inhibitory consortia in future studies. Previous work has demonstrated mechanisms of *C. difficile* inhibition by bile acid transformations (Buffie *et␣al*, 2014) and mucosal sugar competition (Pereira *et␣al*, 2020). Our results demonstrate that communities with high richness, communities that acidify the environment, and communities that compete over limiting resources utilized by *C. difficile* are promising candidates for inhibiting *C. difficile*. In sum, these results suggest multiple target phenotypes that could be combined to design an optimal defined bacterial therapeutic to inhibit *C. difficile*.

To collect data on many multispecies communities to train our model, we cultured communities for a single batch culture cycle. This experimental design is informative for deciphering microbial interactions since the system is farthest away from steady state in the first batch culture cycle and thus has rich dynamic behaviors. Although we did not characterize the long‐term dynamics of multispecies communities using multiple dilution cycles, we are able to draw many insights into *C. difficile*’s interactions with synthetic gut communities on the shorter timescale. Notably, *C. difficile* was the only species that was inhibited by all other community members. Infection by *C. difficile* disrupts the environment of gut bacteria by causing diarrhea (i.e., reduces residence time for gut bacteria), inducing intestinal inflammation, and altering the resource landscape (Fletcher *et␣al*, 2021), suggesting the possibility that gut bacteria have evolved to negatively impact the growth of *C. difficile* in order to promote their fitness in the gut.

Studies have shown that gut microbiomes of patients with CDI have significantly lower richness than healthy controls (Chang *et␣al*, 2008; Antharam *et␣al*, 2013), but this association does not distinguish whether CDI reduces the richness of gut microbiomes or low‐richness microbiomes are more susceptible to CDI. The striking trend between richness and *C. difficile* abundance in our data (Fig 2A) suggests that low‐richness microbiomes are more susceptible to CDI. Supporting this hypothesis, the susceptibility of low‐richness communities to invasion has been demonstrated in other microbial systems (van Elsas *et␣al*, 2012; Mallon, Poly, *et␣al*, 2015). This suggests that the reduction in gut microbiota richness by antibiotic treatment (Dethlefsen & Relman, 2011) could underlie the increased CDI risk that occurs after antibiotic use (Eze *et␣al*, 2017). Additionally, the efficacy of FMTs may be due in part to the high richness of stool samples which are estimated to have greater than one hundred species (Qin *et␣al*, 2010).

Based on our work, high richness communities would be the most effective bacterial therapeutics to inhibit *C. difficile* colonization. The scalable manufacturing of high richness bacterial therapeutics is challenging, indicating the need for new manufacturing techniques to reliably culture communities that maintain all species as opposed to standard culturing of single species. Nevertheless, if manufacturing of high richness communities remains a challenge, our work suggests it is possible to design low‐richness inhibitory communities. While all high richness communities (eight species or more) excluded *C. difficile* in our system, we found low‐richness communities that excluded *C. difficile*. For example, the 3–member Community I excluded *C. difficile* as effectively as the full community (Fig 3B). Corroborating these results, low‐richness communities as small as 5–7 members have been shown to inhibit *C. difficile in␣vitro* and in murine models (Lawley *et␣al*, 2012; Buffie *et␣al*, 2014; Pereira *et␣al*, 2020).

We demonstrated significant variation in the relationship between species abundance and richness across species in our community (Fig 2E and F). These trends are not explained by species growth rate (Fig EV2B) or sensitivity to pH (Fig EV5C). In addition, this variation in sensitivity to richness is not fully explained by patterns in the inferred interspecies interaction network. In our system, the abundance of *B. hydrogenotrophica* and *E. lenta* did not vary with richness. Notably, these species are unique in their ability as an acetogen to utilize hydrogen and carbon dioxide (Bernalier *et␣al*, 1996) and utilize arginine (Sperry & Wilkins, 1976), respectively. Therefore, orthogonal ecological niches may represent one mechanism of low sensitivity in abundance to richness. Further, the plasticity of a species’ ecological niche in response to competition may influence its relationship between abundance and richness. Future work will investigate these potential mechanisms determining the relationship between abundance and richness in larger communities and *in␣vivo*.

*Bacteroides* have been found to both inhibit and promote *C. difficile* growth in different environments (Ferreyra *et␣al*, 2014; Ghimire *et␣al*, 2020; Pereira *et␣al*, 2020; Hassall *et␣al*, 2021), but in our system all *Bacteroides* species inhibited *C. difficile*. We did not observe a strong inhibition of *C. difficile* by *C. scindens* which has been previously shown to occur via production of secondary bile acids that inhibit *C. difficile* germination (Buffie *et␣al*, 2014) presumably due to the absence of bile acids in our media. Instead, in our system the closest relative of *C. difficile*, *C. hiranonis*, was the strongest inhibitor of *C. difficile* abundance. Addition of one of the metabolites utilized by both *C. difficile* and *C. hiranonis*, glucose, partially rescued *C. difficile* growth in *C. hiranonis* spent media (Fig 5E), indicating glucose competition could be one mechanism of *C. difficile* inhibition. Inhibitory consortia could be designed to maximize resource competition over carbohydrates such as glucose between resident members and *C. difficile*. Because an estimated 20% of carbohydrates such as glucose escape absorption by the host and are present in the colon (Stephen *et␣al*, 1983; Anderwald *et␣al*, 2011), carbohydrate competition is likely relevant in the colon environment.

We note that three of the co‐utilized metabolites (proline, serine, and threonine, Fig 5C) are amino acids used in Stickland fermentation, a major energy generation process found in *C. difficile* and many other *Clostridia* (Mead, 1971). Recent work shows that the Stickland fermenting *Clostridium bifermentans* suppressed *C. difficile* abundance and prevented mortality in gnotobiotic mice while the non‐Stickland fermenter *Clostridium sardiniense* did not suppress abundance or prevent mortality (preprint: Girinathan *et␣al*, 2020). The study indicates that introducing competition over Stickland metabolites is a relevant mechanism for *C. difficile* inhibition *in␣vivo*. Our data suggest introduction of the gut microbe *C. hiranonis* into a community could potentially intensify competition over the utilized amino acids. Our data also indicate that *C. hiranonis* has an additional unknown mechanism of *C. difficile* inhibition beyond resource competition. The additional mechanism is not due to pH change and does not involve an extracellular protein (Fig 5E). This leads us to speculate that the inhibition could be due to a non‐protein antibiotic produced by *C. hiranonis* that is similar to the tryptophan‐derived antibiotics produced by *C. scindens* and *Clostridium sordellii* (Kang *et␣al*, 2019).

We showed that communities that reduce the external pH below 6.2 inhibit *C. difficile* in a pH‐dependent manner, consistent with studies showing that *C. difficile* has lower viability and rates of sporulation in acidic environments (Wetzel & McBride, 2020; Yuille *et␣al*, 2020). We note that our *in␣vitro* system differs from the human gut, lacking the pH‐buffering secretion of bicarbonate by host intestinal epithelial cells. However, the amount of bicarbonate buffer in our media (4.8 mM) is within the estimated range in the gastrointestinal tract (2–20 mM) (Litou *et␣al*, 2020). Additionally, the changes in pH that we observe in our *in␣vitro* measurements are within the variation seen in the colon, which has been shown to fluctuate between pH 5 and pH 8 (Koziolek *et␣al*, 2015). This suggests the observed pH changes in our experiments could be physiologically relevant. We also note an intriguing human cohort study that found a strong association between alkaline fecal pH and CDI (Gupta *et␣al*, 2016). Although it is not known whether alkaline pH is a cause of consequence of CDI, this study together with our data suggests that strategies for pH‐based inhibition of *C. difficile* in the colon are worth further investigation. If reduced pH can inhibit *C. difficile* in the colon, manipulation of the pH of the gut environment is a potential microbiome intervention strategy to inhibit *C. difficile*. The pH could be manipulated by bacterial therapeutics containing strong fermenters or dietary substrates that increase fermentation (Chung *et␣al*, 2007).

While propagule pressure has been shown to determine invasion success in microbial invasions (Acosta *et␣al*, 2015; Ketola *et␣al*, 2017; Kinnunen *et␣al*, 2018), we demonstrate that this applies to *C. difficile* in synthetic gut communities. Propagule pressure is known to be important in murine *C. difficile* infections, where mice cohoused with supershedders containing 10^8^ CFU/g *C. difficile* in their feces became colonized with *C. difficile*, whereas mice cohoused with low shedders containing 10^2^ CFU/g *C. difficile* did not become colonized (Lawley *et␣al*, 2009). However, the relationship between *C. difficile* dosage and incidence of CDI in humans is unknown. Our results suggest that the density of *C. difficile* could be an important variable in the outcome of *C. difficile* invasions in a clinical setting. While our data suggest the variation in *C. difficile* abundance with propagule pressure can result in long‐term history dependence (Fig EV3A), the effects of propagule pressure are transient in the model due to the absence of multistability (Fig EV3B). Our model is not informed by long‐term multispecies community measurements and therefore may not accurately capture the long‐term dynamics of the system. Future work will determine the long‐term effects of propagule pressure on *C. difficile* abundance using passaging or continuous culture and develop computational models that can accurately predict these history‐dependent behaviors.

Our absolute abundance method combines OD600 measurements and 16S rRNA gene sequencing to determine the absolute abundance of each species in multispecies communities. Biases in genome extraction efficiency, 16S rRNA gene copy number, and PCR amplification can impact measurements based on 16S rRNA gene sequencing (Crosby & Criddle, 2003; Laursen *et␣al*, 2017; Lim *et␣al*, 2018). We tested for potential bias in our workflow by measuring the relative abundance of mixed cultures containing 10% *C. difficile* based on OD600 measurements (Appendix␣Fig S7). These results indicate there was no significant bias in our method for these communities. Previous work using this absolute abundance method found that 75% of interactions were in qualitative agreement with sterile supernatant experiments (Venturelli *et␣al*, 2018). Further, 85% of our inferred interspecies interactions in the Full Model were in qualitative agreement with this previous study that studied a 12‐member subset of this community (Appendix␣Fig S8). Taken together, these results indicate that our absolute abundance method is reproducible across multiple studies and can decipher biologically meaningful interspecies interactions despite any potential biases.

In sum, we identified ecological and molecular mechanisms of resistance to invasion by *C. difficile* using a synthetic gut microbiome. While our system lacks the full diversity of the human gut microbiome and a host–interaction component, many of our results support principles of invasion theory based on a broad range of systems, suggesting that some of these principles could be generalized to the mammalian gut environment. Future work could create panels of gut microbial communities that feature different weightings of the inhibitory mechanisms identified in this work. These panels could be tested *in␣vitro* for inhibition of *C. difficile* growth and promising candidates could be introduced into germ‐free mouse models to evaluate their *C. difficile* inhibitory potential as bacterial therapeutics.

## Materials and Methods

### Strain and media information

The strains used in this work were obtained from the sources listed in Appendix␣Table S3. The three clinical *C. difficile* isolates (MS002, MS010, and MS011) were *C. difficile* NAAT (GeneXpert) positive via admission stool sample and toxin A (tcdA) and toxin B (tcdB) positive via in‐house research PCR. Each patient was diagnosed with and treated for CDI. Single‐use glycerol stocks were prepared as described previously (Clark *et␣al*, 2021). The media used in this work are anaerobic basal broth (ABB, Oxoid), clostridial reinforced medium (CRM, Difco), YP broth (Geva‐Zatorsky *et␣al*, 2015), and YBHI. YBHI broth recipe: BHI broth (Accumedia), 5 g/l yeast extract (BD Bacto), 1 g/l d‐cellobiose (Chem‐Impex), 1 g/l d‐maltose monohydrate (Sigma‐Aldrich), and 0.5 g/l l‐cysteine (Sigma‐Aldrich).

### Starter cultures

Cells were cultured in an anaerobic chamber (Coy Lab products) with an atmosphere of 2.5 ± 0.5% H_2_, 15 ± 1% CO_2,_ and balance N_2_. Single‐species starter cultures were inoculated by adding 100 µl of a single‐use 25% glycerol stock to 5 ml of anaerobic basal broth media (ABB). *E. rectale* starter cultures were supplemented with 33 mM sodium acetate (Sigma‐Aldrich) and *D. piger* starter cultures were supplemented with 28 mM sodium lactate (Sigma‐Aldrich) and 2.7 mM magnesium sulfate (Sigma‐Aldrich). To begin experiments with organisms in similar growth phases, starter cultures were inoculated either 16 or 41 h prior to experimental setup, depending on the growth rate of the organism (Appendix␣Table S3).

### Inoculation of monospecies and pairs experiments

Starter cultures were diluted to 0.0022 OD600 in ABB (Tecan Infinite Pro F200). For monospecies in Exp1, diluted cultures were added directly to 96 deep well plates for final OD600 of 0.0022. For pairs in Exp2, diluted cultures were combined into pairs in 96 deep well plates at 1:1 or 1:10 volume ratios for final OD600 of 0.0011 or 0.00022 and 0.00198. Cultures were combined using a liquid handling robot (Tecan Evo 100). Plates were covered with gas‐permeable seal (BreatheEasy) and incubated at 37°C with no shaking.

### Inoculation of multispecies community experiments

Starter cultures were diluted to 0.0066 OD600. Diluted cultures were combined into communities in 96 deep well plates using a liquid handling robot (Tecan Evo). The communities in Exp3 and Exp4 were created by combining equal volumes of each diluted starter culture, so the initial OD600 of each species in the community was 0.0066 divided by the number of species. The communities in Exp5 and Exp7 were combined so that all non‐*C. difficile* species had an initial OD600 of 0.00165, and *C. difficile* had an initial OD600 of 0.00055 (10% of community) in the low‐density condition of Exp5 and 0.009 (65% of community) in the high‐density condition of Exp5. In Exp7, the community OD600 was measured after 6 h of incubation and *C. difficile* was added so that its OD600 was 10% (low‐density condition) or 33% (high‐density condition) of the community. The 3–4 member communities in Exp6 were combined such that all non‐*C. difficile* species had an initial OD600 of 0.00165, and *C. difficile* had an initial OD600 of 0, 0.00026, 0.00055, 0.0012, 0.0021, 0.0033, 0.00495, and 0.0074 in the 3 member communities and 0, 0.00035, 0.00073, 0.00165, 0.0028, 0.0044, 0.0066, and 0.0099 in the 4 member communities for initial fractions 0, 0.1, 0.2, 0.3, 0.4, 0.5, and 0.6, respectively. The full community in Exp6 was combined so that all non‐*C. difficile* species had an initial OD600 of 0.00047, and *C. difficile* had an initial OD600 of 0, 0.00032, 0.0015, 0.0026, 0.0041, 0.0061, 0.0092 for initial fractions 0, 0.1, 0.2, 0.3, 0.4, 0.5, and 0.6, respectively. The *C. hiranonis* titration communities in Exp8 were combined so that all non‐*C. hiranonis* species had an initial OD600 of 0.00165, and *C. hiranonis* had an initial OD600 of 0, 0.00055, 0.00012, 0.0021, 0.0033, 0.0050, 0.012, and 0.045 for initial fractions 0, 0.1, 0.2, 0.3, 0.4, 0.5, 0.7, and 0.9, respectively. Plates were covered with gas‐permeable seals (BreatheEasy) and incubated at 37°C with no shaking.

### Culture sample collection

At each timepoint, samples were mixed and aliquots were removed for sequencing and for measuring OD600. We measured OD600 of two dilutions of each sample and selected the value that was within the linear range of the instrument (Tecan Infinite Pro F200). Sequencing aliquots were spun down aerobically at 1,739 *g* for 15 min and stored at −80°C. For timepoints with dilutions, samples were mixed and aliquots were collected for sequencing and OD600 measurements before the samples were diluted 1:20 into fresh media. Abundance of the diluted sample was calculated by dividing the undiluted measurements by the dilution factor of 20.

### pH measurements and adjustments

The pH of each community in Fig 4D was measured using a phenol red assay as described previously (Clark *et␣al*, 2021). The pH of each supernatant in Fig 4E was measured using a pH probe (Mettler Toledo). The pH of each supernatant was adjusted to the pH of fresh media by adding small volumes of sterile 5 M NaOH and 5 M HCl.

### Supernatant experiments

Starter cultures were diluted to 0.0066 OD600. Diluted cultures were combined into communities in 96 deep well plates using a liquid handling robot (Tecan Evo). Communities were created by combining equal volumes of each species, so the final OD600 of each species in the community was 0.0066 divided by the number of species. Plates were covered with gas‐permeable seal (BreatheEasy) and incubated at 37°C with no shaking. After incubation time of 6 h (Fig 4E and F) or 20 h (Fig 5C), cultures were spun down aerobically at 3,500 rpm for 15 min and sterile filtered using Steriflip 0.2‐µM filters (Millipore‐Sigma) before returning to anaerobic chamber. Media controls were spun down and filtered aerobically in parallel with samples. *C. difficile* was inoculated in the sterilized supernatants to a final OD600 of 0.0022 in 96‐well microplates that were covered with gas‐permeable seals (BreatheEasy), incubated at 37°C with shaking, and OD600 was measured every 2 h (Tecan Infinite Pro F200).

### Quantification of metabolites in *C. difficile* and *C. hiranonis* supernatants

*Clostridioides difficile* and *C. hiranonis* starter cultures were diluted to 0.0022 OD600 in ABB in triplicate. After 20 h of incubation, an aliquot of each culture and an aliquot of a media control were spun down aerobically at 3,500 rpm for 15 min and sterile filtered using Steriflip 0.2‐µM filters. The samples were stored at 4C overnight. The supernatants were then diluted 1:50 into water and analyzed by LC‐MS. For Fig 5D and E, a five‐point standard curve of mixed metabolite standard containing acetyl‐ornithine, glucose, glutamine, proline serine, sodium pyruvate, and threonine was prepared in water day‐of and analyzed alongside samples.

LC‐MS analysis was conducted as described previously (27, 80) using a Vanquish UPLC system (Thermo Scientific) coupled to a Q Exactive Orbitrap high‐resolution mass spectrometer (Thermo Scientific) using an electrospray ionization source operating in negative mode. Separation was achieved using a 2.1‐ by 100‐mm Acquity UHPLC BEH C18 column with 1.7‐µm particle size (Waters) at 25°C. Solvent A was 97:3 H2O‐methanol with 10 mM tributylamine adjusted to pH 8.2 by the addition of acetic acid to ˜10 mM final concentration. Solvent B was 100% methanol. The following gradient was used for separation: 0 to 2.5 min, 5% B; 2.5–17 min, linear gradient from 5% B to 95% B; 17–19.5 min, 95% B; 19.5–20 min, linear gradient from 95% B to 5% B; 20–25 min, 5% B. Mass spectrometry parameters were full MS‐SIM (single‐ion monitoring) scanning between 70 and 1,000 *m/z*, automatic control gain (ACG) target of 1e6, maximum injection time (IT) of 40 ms, and resolution of 70,000 full width at half‐maximum (FWHM). MAVEN software suite was used to analyze the data (81, 82). Compounds were identified by retention time matching to pure standards and monoisotopic mass.

For Fig 5C, secreted metabolites were defined as metabolites whose average signal in the three supernatant replicates was at least twofold greater than the signal of the media control. Utilized metabolites were defined as metabolites whose average signal in the three supernatant replicates was at least twofold less than the signal of the media control.

For Fig 5D, metabolite concentrations were calculated using the linear region of the standard curve. The difference in concentration between the supernatant and media control was used to calculate the amount of metabolite needed to add to the supernatant to adjust to media level. The supernatants in Fig 5E were adjusted by adding 20 µl of additive solution to 180 µl supernatant. The additive solutions contained between 1 and 7 metabolites diluted to the necessary concentrations in water. For the no‐treatment control, 20 µl of water was added to 180 µl supernatant. For the "1× media” condition, 20 µl of 10× media was added to 180 µl supernatant.

### Genome extractions

Genomic DNA was extracted using a method adapted from previous work (Clark *et␣al*, 2021). Briefly, cell pellets were resuspended in 180‐µl enzymatic lysis buffer containing 20 mg/ml lysozyme (Sigma‐Aldrich), 20 mM Tris–HCl pH 8 (Invitrogen), 2 mM EDTA (Sigma‐Aldrich), and 1.2% Triton X‐100 (Sigma‐Aldrich). Samples were incubated at 37°C at 600 RPM for 30 min. Samples were treated with 25 µl 20 mg/ml Proteinase K (VWR) and 200 µl buffer AL (Qiagen), mixed by pipette and incubated at 56°C at 600 RPM for 30 min. Samples were treated with 200 µl 200 proof ethanol (Koptec), mixed by pipette, and transferred to 96‐well nucleic acid binding plates (Pall). After washing with 500 µl buffer AW1 and AW2 (Qiagen), a vacuum was applied for 10 min to dry excess ethanol. Genomic DNA was eluted with 110 µl buffer AE (Qiagen) preheated to 56°C and then stored at −20°C.

Genomic DNA was quantified using Sybr Green fluorescence assay with a 6‐point DNA standard curve (0, 0.5, 1, 2, 4, 6 ng/µl biotium). 1 µl of samples and 5 µl of standards were diluted into 95 µl of 1× SYBR green (Invitrogen) in TE buffer and mixed by pipette. Fluorescence was measured with an excitation/emission of 485/535 nm (Tecan Spark). Genomic DNA was normalized to 1 ng/µl in molecular grade water using a liquid handling robot (Tecan Evo 100). Samples < 1 ng/µl were not diluted. Diluted genomic DNA was stored at −20°C.

### Primer design, library preparation, and sequencing

Dual‐indexed primers for multiplexed amplicon sequencing of the 16S v3‐v4 region were designed as described previously (Venturelli *et␣al*, 2018; Clark *et␣al*, 2021). Briefly, oligonucleotides (Integrated DNA Technology) were arrayed into 96‐well plates using an acoustic liquid handling robot (Echo LabCyte) and stored at −20°C. Genomic DNA was PCR‐amplified using Phusion High‐Fidelity DNA Polymerase (Thermo Fisher) for 25 cycles with 0.05 µM of each primer. Samples were pooled by plate, purified (Zymo Research), quantified by NanoDrop, and combined in equal proportions into a library. The library was quantified using Qubit 1× HS Assay (Invitrogen), diluted to 4.2 nM, and loaded at 21 pM onto Illumina MiSeq platform for 300‐bp paired end sequencing.

### Data analysis

Sequencing data were analyzed using a method adapted from previous work (Venturelli *et␣al*, 2018). Basespace Sequencing Hub’s FastQ Generation demultiplexed the indices and generated FastQ files. FastQ files were analyzed using custom python scripts. Paired reads were merged using PEAR (Paired‐End reAd mergeR) v0.9.0 (Zhang *et␣al*, 2014). A reference database containing 16S v3‐v4 region of each species in the study was created by assembling consensus sequence based on sequencing results of each monospecies. Reads were mapped to the reference database using the mothur v1.40.5 command classify.seqs using the Wang method with bootstrap cutoff value of 60% (Wang *et␣al*, 2007; Schloss *et␣al*, 2009). Relative abundance was calculated by dividing the read counts mapped to each organism by the total reads in the sample. Absolute abundance was calculated by multiplying the relative abundance of an organism by the OD600 of the sample. Samples were excluded from further analysis if > 1% of the reads were assigned to a species not expected to be in the community (indicating contamination).

### Generalized Lotka–Volterra Model

The gLV model is a set of *N* coupled first‐order ordinary differential equations:

$$\frac{1}{X_{i}}\frac{dX_{i}}{\mathrm{dt}}=r_{i}+\sum_{j=1}^{N}a_{\mathrm{ij}}X_{j}$$

where *N* is the number of species, the parameter *X_i_* is the abundance of species *i*, the parameter *r_i_* is the basal growth rate of species *i*, the parameter *α_ij_*, called the interaction parameter, is the growth modification of species *i* by species *j* and the parameter *X_j_* is the abundance of species *j*. The parameter *α_ij_* is constrained to be negative when *i* = *j*, representing intra‐species competition.

### Parameter estimation

The gLV model parameters were estimated from time‐series measurements of single‐species and multispecies cultures using the nonlinear programming solver FMINCON in MATLAB, which finds the optimal set of parameters that minimizes a given cost function. The estimation was implemented using previously developed custom MATLAB scripts (Clark *et␣al*, 2021). The cost (C) of the optimization algorithm was computed by (i) simulating each species *m* in each community *k* with an ODE solver and summing the mean‐squared error between the abundance of each species in the simulation *X_model_* and data *X_exp_* at each timepoint *n* (ii) adding the sum each parameter *θ* squared multiplied by a regularization coefficient λ:

$$C=\sum_{k}\sum_{m}\sum_{n}{\left({\hat{X}}_{\exp,m,n}‐X_{\;\mathrm{mod}el,m,n}\right)}^{2}+\lambda \sum_{j}\theta_{j}^{2}$$

The second step is a L2 regularization, which penalizes the magnitude of the parameter vector to prevent overfitting the data. The optimization was repeated with a range of regularization coefficients. The regularization coefficient that resulted in a parameter set with a mean‐squared error of 110% of the non‐regularized parameter set was selected, which was λ = 0.5 for the Preliminary model and λ = 0.1 for the Full Model. The data used for parameter estimation for the Preliminary model and Full Model are given in Table 1. To validate the predictive ability of the model, 24 2–13 member resident communities (Appendix␣Fig S4A) were left out from the training data set and a set of parameters was inferred from this reduced data set using λ = 0.1 for the regularization coefficient. The community compositions of the 24 held‐out communities were simulated with this parameter set to evaluate the predictive capability of the model on held‐out data (Fig 2C).

### Analysis of gLV model

To quantify the uncertainties in gLV parameters, an adaptive Markov chain Monte Carlo (MCMC) method was used to sample from the posterior gLV parameter (θ) distribution *P*(θ|**y**) given a sequence of *m* abundance measurements **y** = (**y**_1_,…,**y**_m_). In particular, for the *k*‐th measurement, **y** *_k_* is a vector that concatenates all abundance measurements collected from all sub‐community experiments. Uncertainty for the *k‐*th measurement was modeled by an additive and independent noise, which is distributed according to *N*(0, $\sigma_{k}^{2}$), where $\sigma_{k}^{2}$ is the diagonal covariance matrix for experimental data collected in the *k‐*th measurement. Given a fixed parameter θ, the gLV model was simulated to obtain the model predicted abundance ${\bar{y}}_{k}\left(\theta \right)$ at every instant *k*. The likelihood to observe a sequence of abundance measurements **y** was then computed as:

$$P\left(y|\theta \right)=\prod_{k=1}^{m}f(y_{k}‐{\bar{y}}_{k}\left(\theta \right);\sigma_{k}),$$

where $f(\cdot \;;\sigma_{k})$ is the probability density function for the normal distribution $N(0,\sigma_{k}^{2})$. The posterior distribution was then described according to Bayes rule as P(θ|y)∝P(y|θ)P(θ), where *P*(θ) is the prior parameter distribution. Normal priors were used for the parameters. The means of the normal distributions were set to the parameters estimated by the FMINCON method and the coefficients of variation were set to 5%.

An adaptive, symmetric, random‐walk Metropolis MCMC algorithm (Haario *et␣al*, 2001) was then used to draw samples from this posterior distribution. Specifically, given the current sample θ^(^ *^n^* ^)^ at step *n* of the Markov chain, the proposed sample for step (*n* + 1) is θ^(^ *^n^* ^+1)^ = θ^(^ *^n^* ^)^+δ^(^ *^n^* ^)^, where δ^(^ *^n^* ^)^ is drawn from a normal distribution. The algorithm is adaptive in the sense that the covariance of this normal distribution is given by $\alpha \cdot \gamma_{n}^{2}$, where $\gamma_{n}^{2}$ is the covariance of θ^(1)^,…, θ^(^ *^n^* ^)^ and *α* is a positive parameter. The proposed sample is accepted with probability 1 if *P*(θ^(^ *^n^* ^+1)^|**y**)/*P*(θ^(^ *^n^* ^)^|**y**) > 1, and it is accepted with probability *β* if *P*(θ^(^ *^n^* ^+1)^|**y**)/*P*(θ^(^ *^n^* ^)^|**y**) = *β* ≤ 1.

The algorithm described above was implemented using MATLAB R2020a, where the gLV models were solved using variable step solver ode23s. 120,000 MCMC samples were collected after a burn‐in period of 10,000 samples. The Gelman–Rubin potential scale reduction factor (PSRF) was used to evaluate convergence of the posterior distribution estimates, where a PSRF closer to 1 indicates better convergence. The average PSRF is 1.31 and 80% of the parameters have a PSRF < 1.5. The medians of the marginal distributions of all parameters correlated strongly with parameters estimated by the FMINCON method (Pearson *r* = 0.99).

To evaluate multistability in our gLV model, we enumerated all non‐negative equilibria of the subcommunities and then evaluated their local stability by checking the eigenvalues of the Jacobian at the respective equilibria.

### Hill equation␣and exponential decay model fits

The community sensitivity to *C. difficile* initial abundance was quantified by fitting the data to the Hill equation:

$$\frac{E}{E_{\max}}=\frac{A^{n}}{EC_{50}^{n}+A^{n}}$$

where *E* is 48‐h abundance of *C. difficile*, *E_max_* is the maximum 48‐h abundance of *C. difficile* across all initial fractions, *A* is the initial fraction of *C. difficile*, *EC_50_* is the initial fraction that produces 50% of *E_max_* value, and *n* is a measure of ultrasensitivity. The data were fit using custom python scripts implementing the curve_fit function of the scipy package optimization module.

The relationship between OD600 and species richness was quantified by fitting the experimental data or simulated data to an exponential decay function:

$$y=ae^{‐bx}$$

where *y* is calculated OD600, *a* is initial calculated OD600, *b* is the exponential decay constant, and *x* is species richness. The OD600 data were normalized to be between 0 and 1 for all species. The data were fit using custom python scripts implementing the curve_fit function of the scipy package optimization module.

### Normalized Euclidean distances

The normalized Euclidean distance (D) between uninvaded resident community *R* and *C*. *difficile*‐invaded community *V* is calculated using.

$$D\left(R,\;V\right)=\sum_{i}\sqrt{{\left(R_{i}‐V_{i}\right)}^{2}}$$

here *R* is the 48‐h timepoint of the uninvaded resident community and *V* is the 48‐h timepoint of the resident community invaded with *C. difficile*. *R_i_* is the relative abundance of species *i* in the uninvaded resident community, equal to reads of species *i* divided by the total community reads. *V_i_* is the normalized relative abundance of species *i* in the invaded community, equal to reads of species *i* divided by the resident community reads (total community reads minus *C. difficile* reads).

## Author contributions

OSV and SH conceived the study. SH carried out the experiments. SH, OSV, TBJ, and DA‐N designed and SH and TBJ implemented the metabolomic measurements. SEH and YQ performed computational modeling and analysis. RLC and YQ wrote customized scripts to perform modeling analyses. SH and OSV analyzed and interpreted the data. OSV secured funding. NS and LW isolated clinical *C.difficile* strains and provided information on these strains. SEH and OSV wrote the paper and all authors provided feedback on the manuscript.

## Conflict of interest

The authors declare that they have no conflict of interest.

## Supporting information



Appendix

Expanded View Figures PDF

Dataset EV1

Dataset EV2

Dataset EV3

Dataset EV4

## Data Availability

The datasets and computer code produced in this study are available in the following databases:
Community composition data: Dataset EV4 in this manuscript. Metabolomics data: Dataset EV3 in this manuscript. Modeling computer scripts: https://github.com/SusanHromada/NegativeInteractionsDetermineCdifficileGrowth.
