# Supplementary material for: Negative interactions determine Clostridioides difficile growth in synthetic human gut communities
Source: Mol Syst Biol. 2021 Oct 25;17(10):e10355. doi: 10.15252/msb.202110355 (PMC8543057; doi:10.15252/msb.202110355)
Supplement: Supplementary file 1 — Appendix [file MSB-17-e10355-s007.pdf]

Appendix for

Negative interactions determine *C. difficile* growth in synthetic human gut communities

Susan Hromada[1,2], Yili Qian[1], Tyler Jacobson[2], Ryan L. Clark[1], Lauren Watson[3,4], Nasia Safdar[3,4], Daniel Amador-Noguez[2], and Ophelia S. Venturelli[1,2,5*]

[1]Department of Biochemistry, University of Wisconsin-Madison, Madison, WI, USA
[2]Department of Bacteriology, University of Wisconsin-Madison, Madison, WI, USA
[3]Division of Infectious Disease, Department of Medicine, School of Medicine and Public Health, University of Wisconsin-Madison, Madison, WI, USA
[4]Department of Medicine, William S. Middleton Veterans Hospital Madison, Madison, WI, USA
[5]Department of Chemical and Biological Engineering, University of Wisconsin-Madison, Madison, WI, USA

Table of Contents

Appendix Figure S1 2
Appendix Figure S2 2
Appendix Figure S3 3
Appendix Figure S4 4
Appendix Figure S5 5
Appendix Figure S6 6
Appendix Figure S7 6
Appendix Figure S8 7
Appendix Table S1 8
Appendix Table S2 9
Appendix Table S3 10
Appendix Table S4 11

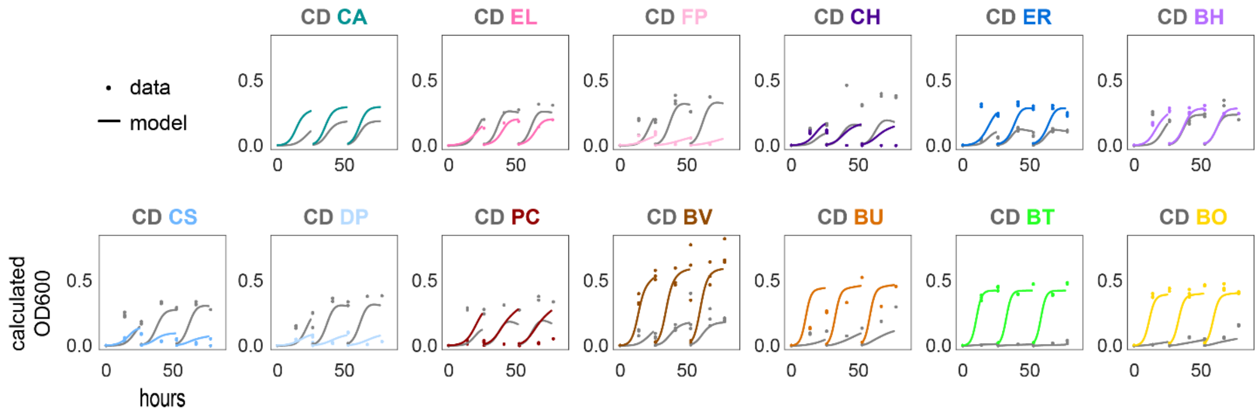

Appendix Figure S1: *C. difficile* introduced into pairwise communities with low initial density coexists with majority of resident gut species.

Absolute abundance (calculated OD600) of species over time for three growth cycles. First growth cycle inoculated at a 1:9 ratio of *C. difficile* to resident species based on OD600 measurements. Datapoints indicate experimental biological replicates. Lines indicate simulations using the generalized Lotka-Volterra Full Model. Calculated OD600 is the product of 16S relative abundance and community OD600.

Data information: *n*=2-3 biological replicates (See Appendix Table S4 for replicate information of each condition).

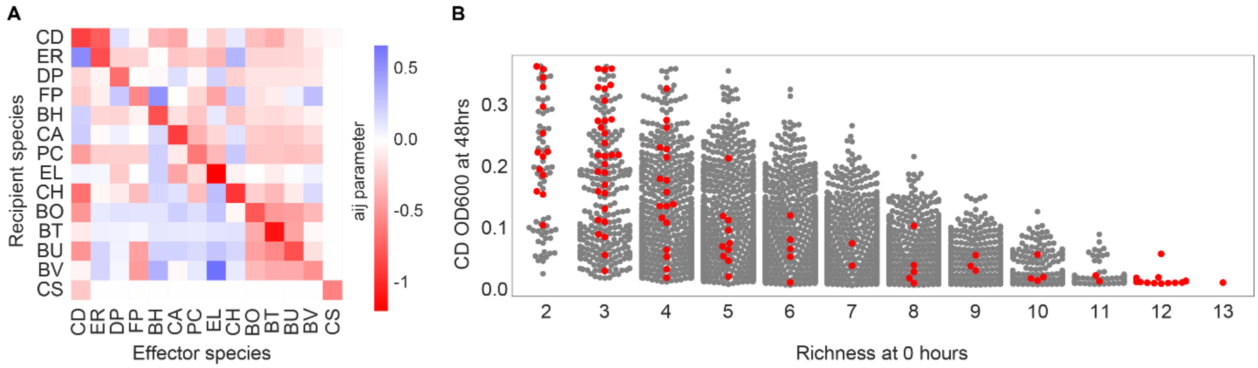

Appendix Figure S2: Generalized Lotka-Volterra model parameters for Preliminary Model and predictions of species abundance.

A: Heatmap of inferred interspecies interaction parameters of Preliminary Model.

B: Swarmplot of simulated *C. difficile* absolute abundance (OD600) at 48 hours using the Preliminary model as a function of initial resident species richness for all 8,178 possible *C. difficile*-containing subcommunities composed of 2-13 species. Red points indicate communities chosen for experimental characterization.

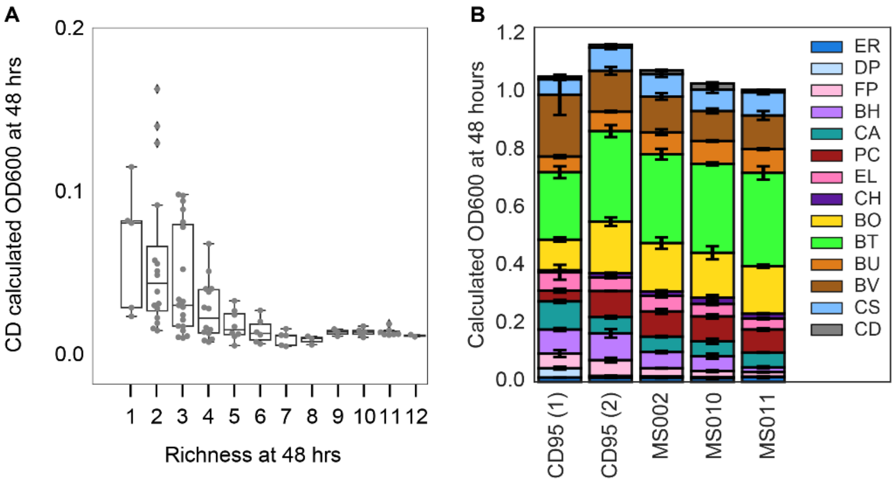

Appendix Figure S3: Analysis of 48-hour community composition of communities assembled with *C. difficile*.

A: Swarmplot of *C. difficile* (CD) absolute abundance (calculated OD600) at 48 hours in 94 sub-communities as a function of species richness at 48 hours. Datapoints indicate mean of biological replicates. Line represents median, box edges represent first and third quartiles, and whiskers indicate the minimum and maximum. Outliers are denoted by diamonds.Calculated OD600 is the product of 16S relative abundance and community OD600.

B: Barplot of community composition of full community containing one of four different strains of *C. difficile* (**Table S1**). CD95 (1) condition had an initial total OD600 of 0.0066 and initial evenness of 1. CD95 (2) and MS conditions had an initial *C. difficile* OD600 of 0.00032 and initial non-*C. difficile* species OD600 of 0.00047. Bars indicate the mean and error bars indicate one s.d. from the mean of biological replicates. Calculated OD600 is the product of 16S relative abundance and community OD600.

Data information: In A, *n*=1-3 biological replicates. In B, *n*=3 biological replicates.

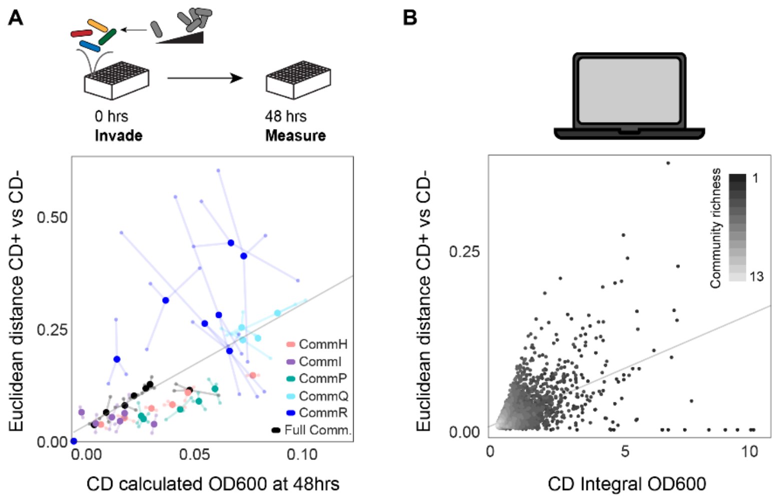

Appendix Figure S4: Change in resident community composition in the presence and absence of *C. difficile*.

A: Scatterplot of normalized Euclidean distance (**Methods**) between communities initialized with various *C. difficile* abundances and the uninvaded communities as a function of *C. difficile* absolute abundance (calculated OD600) in the community at 48 hours. Gray line indicates a linear regression (y=2.7x+0.02, Pearson r=0.61, $p=6*10^{-13}$). Transparent data points indicate biological replicates and are connected to the corresponding mean values by transparent lines. Calculated OD600 is the product of 16S relative abundance and community OD600.

B: Scatterplot of normalized Euclidean distance between the 48 hour abundance of simulated communities invaded with *C. difficile* at six hours and uninvaded communities as a function of simulated *C. difficile* integral OD600 from 0 to 48 hours in the community (y=0.16x+0.004, Pearson r=0.58, p=0.0). Shading indicates species richness.

Data information: A, *n*=1-3 biological replicates (See Appendix Table S4 for replicate information of each condition).

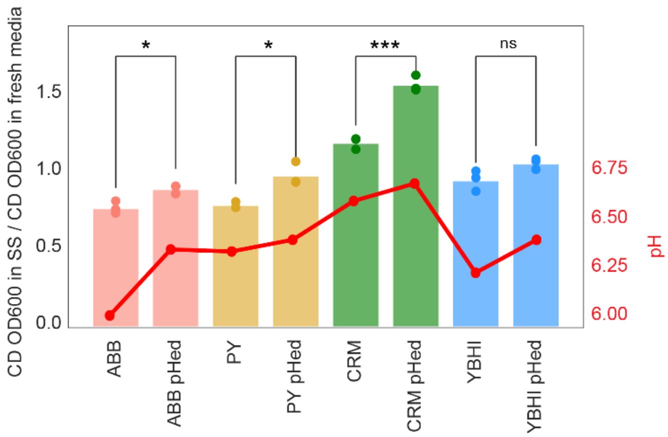

Appendix Figure S5: *C. difficile* growth in CommI supernatants in multiple medias.

Bar plot of fold change of *C. difficile* growth in CommI sterilized supernatants (SS) compared to the growth of *C. difficile* in the corresponding fresh media. Growth was quantified as integral of OD600 from 0 to 20 hours. ABB = Anaerobic basal broth, PY = peptone yeast, CRM = Clostridial reinforced media, YBHI = Yeast Brain Heart Infusion. Datapoints indicate biological replicates and bars indicate mean value. Red line shows pH of supernatants. Stars denote statistical significance: * $p<0.05$, ** $p<0.01$, *** $p<0.001$, ns $p>0.05$ according to an unpaired t-test.

Data information: *n*=2-3 biological replicates (See Appendix Table S4 for replicate information of each condition).

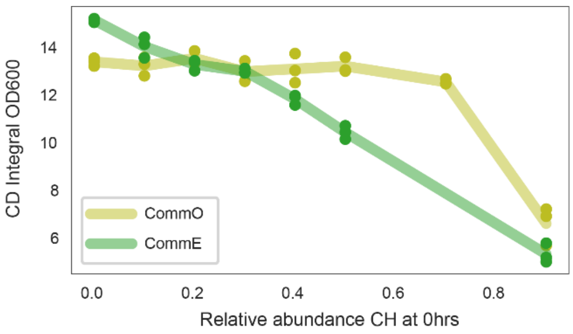

Appendix Figure S6. *C. difficile* growth in CommO and CommE sterilized supernatants.

Lineplot of integral *C. difficile* OD600 over 20 hours incubation in CommO and CommE supernatants. Datapoints represent biological replicates and line represents mean of biological replicates.

Data information: *n*=3 biological replicates.

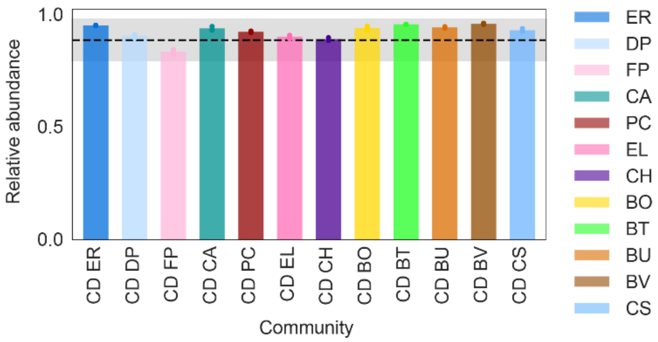

Appendix Figure S7: 16S Sequencing relative abundance of test two-member communities.

Barplot of 16S relative abundance of non-*C. difficile* species in each two-member community. Test communities were assembled in 10:90 CD:non-*C. difficile* species ratio based on OD600 measurements and collected immediately for sequencing. Dash line indicates target relative abundance (0.9) of non-*C. difficile* species. Shaded region represents 10% error of target value.

Data information: *n*=5 biological replicates.

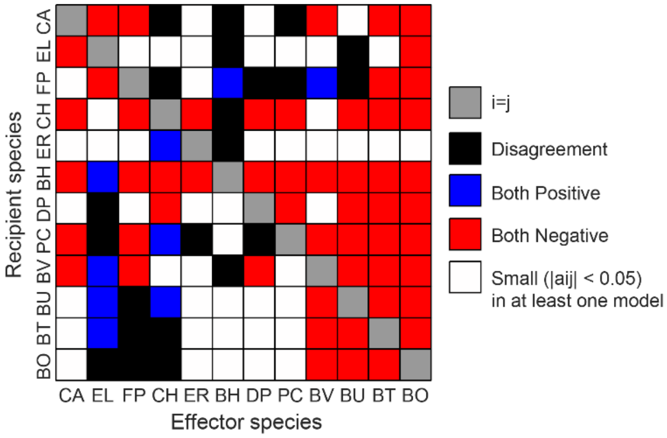

Appendix Figure S8: Heatmap of agreement/disagreement between specific interactions between Full Model and gLV model from Venturelli et al, *Molecular Systems Biology*, 2018.

Legend describes what each color represents.

Appendix Table S1: Community datasets

Dataset	Number of communities	Figure	Richness of resident community	Multiple initial densities?	Time of invasion	In Prelim. Model training data	In Full Model training data
Exp1	14	1D	NA	N	NA	Y	Y
Exp2	13	1E	1	Y	0 hours	Y	Y
Exp3	94	2AE, 3A	2-13	N	0 hours	N	Y
Exp4	110	2E	3-13	N	0 hours	N	N
Exp5	15	3A, 4AB	3, 4	Y	0 hours	N	Y
Exp6	6	3BCD	3, 4, 13	Y	0 hours	N	Y, except for MS002/MS010/ MS011 data
Exp7	15	4ABD	3, 4	Y	6 hours	N	N
Exp8	3	5B	1, 2	Y	0 hours	N	Y

Appendix Table S2: Identity of 3-4 member communities in Dataset 5 and Dataset 7

**Community**	**Species**
CommA	*Desulfovibrio piger, Blautia hydrogenotrophica, Clostridium hiranonis*
CommB	*Desulfovibrio piger, Blautia hydrogenotrophica, Faecalibacterium prausnitzii*
CommC	*Desulfovibrio piger, Blautia hydrogenotrophica, Collinsella aerofaciens, Clostridium hiranonis*
CommD	*Collinsella aerofaciens, Prevotella copri, Eggerthella lenta*
CommE	*Desulfovibrio piger, Eggerthella lenta, Clostridium hiranonis*
CommF	*Desulfovibrio piger, Blautia hydrogenotrophica, Bacteroides uniformis*
CommG	*Eubacterium rectale, Blautia hydrogenotrophica, Collinsella aerofaciens*
CommH	*Desulfovibrio piger, Bacteroides ovatus, Bacteroides vulgatus*
CommI	*Desulfovibrio piger, Bacteroides thetaiotaomicron, Bacteroides uniformis*
CommJ	*Eubacterium rectale, Faecalibacterium prausnitzii, Clostridium scindens*
CommK	*Desulfovibrio piger, Blautia hydrogenotrophica, Bacteroides uniformis, Bacteroides vulgatus*
CommL	*Desulfovibrio piger, Faecalibacterium prausnitzii, Clostridium hiranonis*
CommM	*Desulfovibrio piger, Clostridium hiranonis, Clostridium scindens*
CommN	*Blautia hydrogenotrophica, Prevotella copri, Clostridium hiranonis*
CommO	*Blautia hydrogenotrophica, Collinsella aerofaciens, Clostridium hiranonis*
CommP	*Desulfovibrio piger, Blautia hydrogenotrophica, Eggerthella lenta, Bacteroides uniformis*
CommQ	*Eubacterium rectale, Blautia hydrogenotrophica, Collinsella aerofaciens, Prevotella copri*
CommR	*Blautia hydrogenotrophica, Collinsella aerofaciens, Prevotella copri, Clostridium hiranonis*

Appendix Table S3: Strain information

Abbreviation	Species	Strain	Source	Type Strain?	Preculture Time (hr)
BH	*Blautia hydrogenotrophica*	*Blautia hydrogenotrophica* S5a33 [DSM 10507]	DSMZ	Yes	41
BO	*Bacteroides ovatus*	*Bacteroides ovatus* NCTC 11153 [ATCC 8483]	ATCC	Yes	16
BT	*Bacteroides thetaiotaomicron*	*Bacteroides thetaiotaomicron* VPI 5482 [ATCC 29148]	ATCC	Yes	16
BU	*Bacteroides uniformis*	*Bacteroides uniformis* VPI 0061 [DSM 6597]	DSMZ	Yes	16
BV	*Bacteroides vulgatus*	*Bacteroides vulgatus* NCTC 11154 [ATCC 8482]	ATCC	Yes	16
CA	*Collinsella aerofaciens*	*Collinsella aerofaciens* VPI 1003 [DSM 3979]	DSMZ	Yes	41
CD	*Clostridioides difficile*	*Clostridioides difficile* R20291 [DSM 27147]	DSMZ	No	41
CD MS002	*Clostridioides difficile*	*Clostridioides difficile* Clinical isolate	Safdar lab	No	41
CD MS010	*Clostridioides difficile*	*Clostridioides difficile* Clinical isolate	Safdar lab	No	41
CD MS011	*Clostridioides difficile*	*Clostridioides difficile* Clinical isolate	Safdar lab	No	41
CH	*Clostridium hiranonis*	*Clostridium hiranonis* T0-931 [DSM 13275]	DSMZ	Yes	16
CS	*Clostridium scindens*	*Clostridium scindens* VPI 13733 [DSMZ 5676]	DSMZ	Yes	41
DP	*Desulfovibrio piger*	*Desulfovibrio piger* VPI C3-23 [ATCC 29098]	ATCC	Yes	41
EL	*Eggerthella lenta*	*Eggerthella lenta* 1899 B [DSM 2243]	DSMZ	Yes	16
ER	*Eubacterium rectale*	*Eubacterium rectale* VPI 0990 [ATCC 33656]	ATCC	Yes	16
FP	*Faecalibacterium prausnitzii*	*Faecalibacterium prausnitzii* A2-165 [DSM 17677]	DSMZ	No	41
PC	*Prevotella copri*	*Prevotella copri* CB7 [DSM 18205]	DSMZ	Yes	41

Appendix Table S4: Replicate information

Figure	Replicates
1D	• Passage 1: *n*=3 for all conditions.<br>• Passage 2-3: *n*=2 for CD, n=1 for all other conditions
1E	*n*=1 for CD-CA, *n*=2 for CD-EL, *n*=3 for all other conditions
3B	*n*=3 for all fractions in CommI, CommP, CommR, and Full community.<br>• CommH: *n*=3,3,3,3,3,3,2 for initial fractions 0.0, 0.05, 0.1, 0.2, 0.3, 0.4, 0.6<br>• CommQ *n*=3,3,1,3,3 for initial fractions 0.0, 0.3, 0.4, 0.5, 0.6
4A,B,D	*n*=3 for all conditions for CommA, CommJ, CommK, CommL, and CommM.<br>For conditions LD 0 hour, HD 0 hour, LD 6 hour, and HD 6 hour:<br>• CommB: *n*=2,2,3,3<br>• CommC: *n*=3,1,NA,2<br>• CommD: *n*=3,2,NA,NA<br>• CommE: *n*=3,2,3,1<br>• CommF: *n*=2,3,3,3<br>• CommG: *n*=1,1,3,3<br>• CommH: *n*=1,3,3,3<br>• CommI: *n*=3,2,3,3<br>• CommN: *n*=3,3,NA,NA<br>• CommO: *n*=1,3,2,3
5B	*n*=3 for all conditions in CommO.<br>• CommE: *n*=3,3,3,3,3,3,2,3 for initial fractions 0.0,0.1,0.2,0.3,0.4,0.5,0.7,0.9<br>• CD-CH: *n*=2,1,1,2,1,1,1 for initial fractions 0.0, 0.25, 0.43, 0.56, 0.67, 0.88, 0.96
5E	*n*=5,3,5,3,4,4,5,5,3,3,3,3,3,3,3,3,5,6,63 for None, Acetyl-Ornithine, D-glucose, Glutamine, Proline, Pyruvate, Serine, Threonine, -Acetyl-Ornithine, -D-glucose, -Glutamine, -Proline, -Pyruvate, -Serine, -Threonine, All 7, media spike, None, Heat, Proteinase K
EV3A	*n*=3 for all timepoints in CommA, CommL, and CommN.<br>For timepoints 6hr, 12hr, 48hr:<br>• CommB: HD *n*=3,3,2, LD *n*=3,3,2<br>• CommC: HD *n*=3,3,1, LD *n*=3,3,3<br>• CommD: HD *n*=3,NA,2, LD *n*=3,NA,3<br>• CommE: HD *n*=3,NA,2, LD *n*=3,NA,3<br>• CommF: HD *n*=3,3,3, LD *n*=2,3,2<br>• CommG: HD *n*=3,2,1, LD *n*=3,2,1<br>• CommH: HD *n*=3,3,3, LD *n*=3,3,1<br>• CommI: HD *n*=3,3,2, LD *n*=3,2,3<br>• CommJ: HD *n*=2,3,3, LD *n*=3,2,3<br>• CommK: HD *n*=1,3,3, LD *n*=2,3,3

	• CommM: HD *n*=2,3,3, LD *n*=3,2,3<br>• CommO: HD *n*=3,3,3, LD *n*=3,2,1
EV4E	*n*=3,3,1,3,3 for initial fractions 0.0, 0.3, 0.4, 0.5, and 0.6 respectively.
EV5A	*n*=2 for CD, *n*=3 for all other conditions
S1	*n*=2 for CD-EL, *n*=3 for all other conditions
S4A	*n*=3 for all fractions in CommI, CommP, CommR, and Full community.<br>• CommH: *n*=3,3,3,3,3,3,2 for initial fractions 0.0, 0.05, 0.1, 0.2, 0.3, 0.4, 0.6<br>• CommQ *n*=3,3,1,3,3 for initial fractions 0.0, 0.3, 0.4, 0.5, 0.6
S5	From left to right,<br>*n*=3,3,2,3,3,3,3,3

Table indicates replicate information for figures where conditions have variable number of biological replicates and are feasible to report (<75 conditions). Information for figures where all conditions had equal number of biological replicates are provided in the corresponding figure legend. Information for figures with >75 separate conditions with variable number of biological replicates are provided as a range in the corresponding figure legend. NA = no data for this condition.
