# Supplementary material for: Negative interactions determine Clostridioides difficile growth in synthetic human gut communities
Source: Mol Syst Biol. 2021 Oct 25;17(10):e10355. doi: 10.15252/msb.202110355 (PMC8543057; doi:10.15252/msb.202110355)
Supplement: Supplementary file 2 — Expanded View Figures PDF [file MSB-17-e10355-s001.pdf]

Expanded View Figures

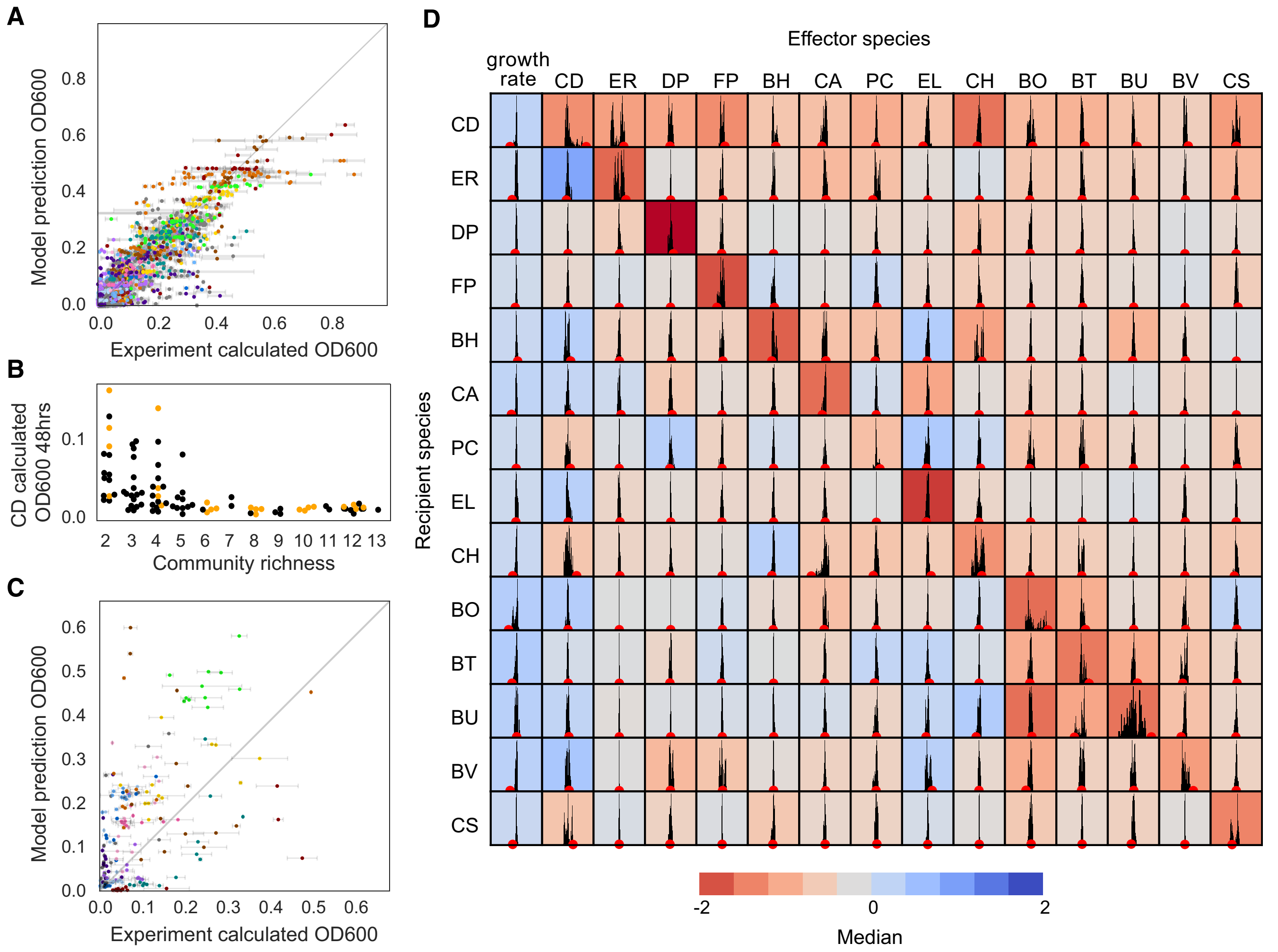

Figure EV1. Analysis of parameter uncertainty and predictive capability of generalized Lotka–Volterra models.

A Scatterplot of goodness of fit of experimental absolute abundance (calculated OD600) versus simulated species absolute abundance using the Full Model for the communities in the training data set (Pearson $r = 0.89$, $P = 0.0$). Error bars represent one SD from the mean of biological replicates. Gray line indicates y = x, or 100% prediction accuracy. Calculated OD600 is the product of 16S relative abundance and community OD600.

B Swarmplot highlighting 24 communities chosen as the held-out set (data also shown in Fig 2A). Orange datapoints represent held-out communities from training set. Black datapoints indicate communities from Fig 2A in training data set. Calculated OD600 is the product of 16S relative abundance and community OD600.

C Scatterplot of experimental absolute abundance (calculated OD600) versus predicted species absolute abundance (OD600) using the Preliminary Model for the 24 held-out communities (Pearson $r = 0.52$, $P = 1*10^{-14}$). Error bars represent one SD from the mean of biological replicates. Gray line indicates y = x, or 100% prediction accuracy. Calculated OD600 is the product of 16S relative abundance and community OD600.

D Histograms of parameter values determined using Metropolis–Hastings Monte Carlo (MCMC) analysis. Red dots indicate the parameter value in the Full Model (Materials and Methods). The black histograms indicate the MCMC distribution. The x-axis is scaled to median of MCMC distribution +/– 0.25. The color of each subplot denotes the median value of the MCMC distribution.

Data information: In A-C, n = 1–3 biological replicates.

 © 2021 The Authors

20 of 19 _Molecular Systems Biology_ **17**: e10355 | 2021 © 2021 The Authors

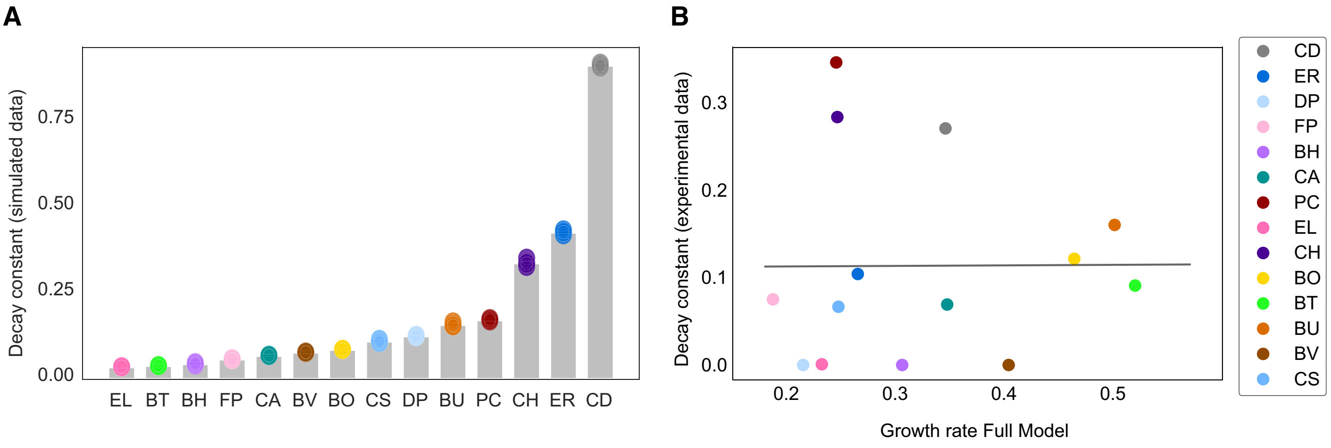

Figure EV2. Analysis of simulated and measured decay constants quantifying the relationship between species richness and abundance.

A Barplot of decay constants b from exponential decay fit to simulated data in 2e. Colored datapoints are best fit parameters from five models, where each model was trained on a subsample of the data consisting of 4/5 of the experimental datapoints. Gray bar indicates best fit parameter from model trained on all data.

B Scatterplot of relationship between species growth rate in the Full Model and decay constant fit to experimental data shown in Fig 2F. Gray line indicates linear regression ($y = 0.006x+0.111$, Pearson $r = 0.01$, $P = 0.98$).

© 2021 The Authors

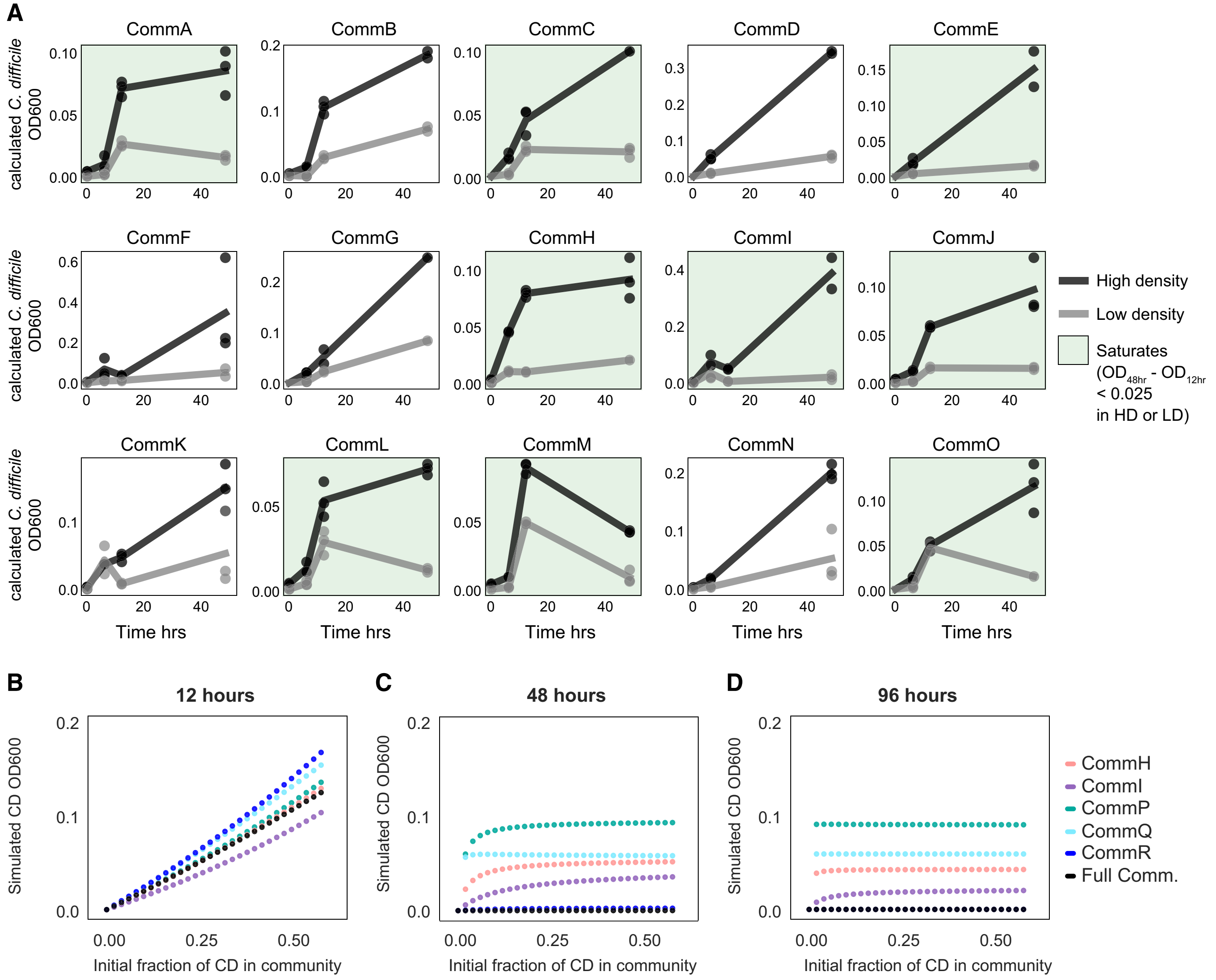

Figure EV3. Dependence of *C. difficile* abundance on propagule pressure over time.

A Lineplots of *C. difficile* (CD) abundance over time in CommA-CommO communities. Final timepoint is same as shown in Fig 3A. In low-density conditions, *C. difficile* inoculated at 10% of total community OD600 at 0 h. In high-density conditions, *C. difficile* inoculated at 65% of total community OD600 at 0 h. Data points indicate biological replicates and lines indicate mean value of biological replicates. Communities where *C. difficile* abundance saturates by 48 h in either high-density or low-density conditions are highlighted in green (saturation defined as difference in *C. difficile* OD600 between 48 and 12 h is less than 0.025).

B–D Lineplots of predicted absolute abundance (OD600) of *C. difficile* at 12, 24, and 96 h as a function of the initial fraction of *C. difficile* as simulated by the Full Model.

Data information: In A, $n = 1$–3 biological replicates (See Appendix Table S4 for replicate information of each condition).

 © 2021 The Authors

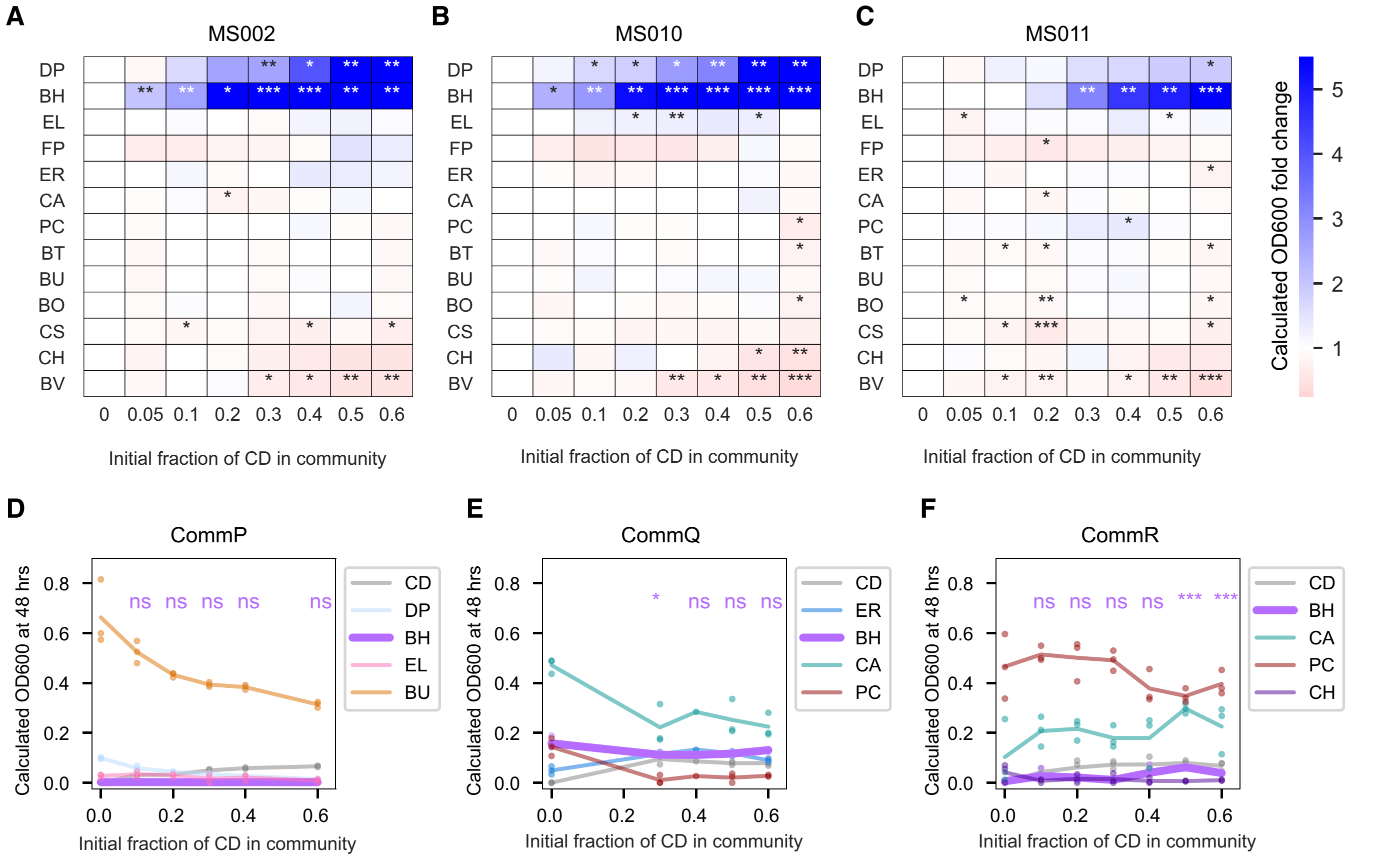

Figure EV4. Impact of *C. difficile* on resident species abundances.

A–C Heatmap of the fold change of species absolute abundance (mean value of biological replicates) in full community with 5–60% initial *C. difficile* (CD) compared to the 0% initial *C. difficile* condition. Stars represent statistical significance: *$P < 0.05$, **$P < 0.01$, ***$P < 0.001$ according to an unpaired *t*-test. A: *C. difficile* strain MS002, B: *C. difficile* strain MS010, C: *C. difficile* strain MS011.

D–F Lineplots of species absolute abundance (calculated OD600) at 48 h as a function of initial *C. difficile* fraction. Datapoints indicate biological replicates and lines indicate the mean. Calculated OD600 is the product of 16S relative abundance and community OD600. Stars indicate a statistically significant difference in the absolute abundance of *B. hydrogenotrophica* compared to the absolute abundance of *B. hydrogenotrophica* in 0% initial *C. difficile* condition: *$P < 0.05$, **$P < 0.01$, ***$P < 0.001$, ns = no significant difference according to an unpaired *t*-test. D: CommP, E: CommQ, F: CommR.

Data information: In A-C, D, F $n = 3$ biological replicates. In E, $n = 1$ or $n = 3$ biological replicates (see Appendix Table S4 for replicate information of each condition).

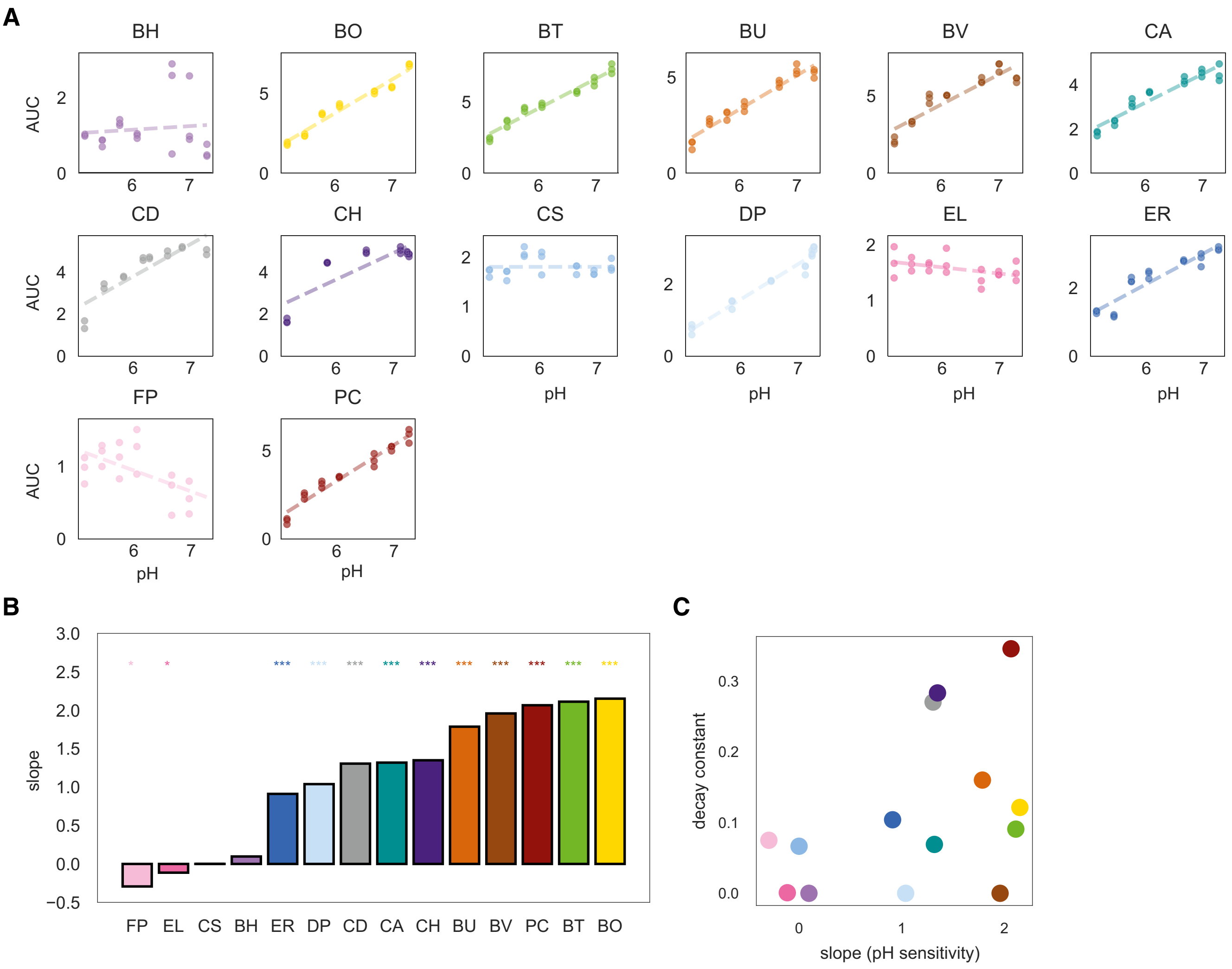

Figure EV5. Resident gut species pH sensitivity in monoculture.

A Lineplots of monospecies growth as a function of the initial environmental pH of adjusted fresh media. Growth is quantified as area under the curve (AUC) from 0 to 20 h. Datapoints indicate biological replicates and dashed lines indicate linear regression fits.

B Barplot of slopes of linear regression fit to data in A. Stars denote statistical significance: $^*P < 0.05$, $^{**}P < 0.01$, $^{***}P < 0.001$ according to an unpaired *t*-test.

C Scatterplot of decay constants from Fig 2F as a function of pH sensitivity slopes in B. Linear regression y = 0.06x+0.05, Pearson $r = 0.44$, $P = 0.11$).

Data information: In A, $n = 2$–3 biological replicates (See Appendix Table S4 for replicate information of each condition).

 © 2021 The Authors
