# Supplementary material for: Negative interactions determine Clostridioides difficile growth in synthetic human gut communities
Source: Mol Syst Biol. 2021 Oct 25;17(10):e10355. doi: 10.15252/msb.202110355 (PMC8543057; doi:10.15252/msb.202110355)
Supplement: Supplementary file 7 [file MSB-17-e10355-s004.pdf]

# Negative interactions determine C. difficile growth in synthetic human gut communities

Susan Hromada, Yili Qian, Tyler Jacobson, Ryan Clark, Lauren Watson, Nasia Safdar, Daniel Amador-Noguez, and Ophelia Venturelli
**DOI: 10.15252/msb.202110355**

*Corresponding author(s): Ophelia Venturelli (venturelli@wisc.edu)*



## Review Timeline:

| | |
|---|---|
| Submission Date: | 19th Mar 21 |
| Editorial Decision: | 26th Apr 21 |
| Revision Received: | 9th Aug 21 |
| Editorial Decision: | 27th Sep 21 |
| Revision Received: | 29th Sep 21 |
| Accepted: | 30th Sep 21 |

*Editor: Maria Polychronidou*


## Transaction Report:

(Note: With the exception of the correction of typographical or spelling errors that could be a source of ambiguity, letters and reports are not edited. Depending on transfer agreements, referee reports obtained elsewhere may or may not be included in this compilation. Referee reports are anonymous unless the Referee chooses to sign their reports.)

**1st Editorial Decision** **26th Apr 2021**

Thank you again for submitting your work to Molecular Systems Biology. We have now heard back from the three referees who agreed to evaluate your study. Overall, the reviewers acknowledge that the topic of the study is relevant. They raise however a series of concerns, which we would ask you to address in a revision.

I think that the recommendations of the reviewers are rather clear and therefore I do not see the need to repeat the points listed below. Several of the reviewers' comments refer to the need to better support the main conclusions and exclude alternative explanations for the reported findings. All issues raised by the reviewers would need to be satisfactorily addressed. Please contact me in case you would like to discuss in further detail any of the issues raised.

On a more editorial level, we would ask you to address the following points:

REFEREE REPORTS

--------------------------------------------------------

Reviewer #1:

This is an interesting study that combines ecological modeling with simple in vitro experiments to study colonization resistance to C difficile. The authors show that diversity from the ecological side, and pH from the mechanistic side are important in their assay. In addition, they find evidence that adding back glucose to cultures can restore C. diff growth in some cases. I am broadly

supportive of publication subject to a few questions/concerns being addressed:

1. As I understand it, the authors use 16s to estimate frequencies of their frequencies which are then multiplied by OD to get absolute abundance. 16s is well known to be subject to significant biases that mean that the frequencies measured do not reflect the actual frequencies of a strain of bacteria in a sample. Were these biases estimated with a test community of known frequencies? Sorry if I missed this, but this issue is worth discussing prominently I think. Also, OD is well known to often fail to reflect cell numbers, particularly at higher densities. Again, did you do any tests to make sure this is not affecting your measurements and estimates, and conclusions?
2. I thought figure 2e and the analyses that are behind it are great. However, I wasn't sure about the conclusion that C. diff has a unique profile therein. PC, CH, ER all also look like they have a strong negative dependence in the full simulated dataset. It comes on at higher diversities admittedly, but it looks like it is there. I wasn't sure why the authors then concluded that this dependence wasn't seen outside of C. diff.
3. The strong effect of pH in the assay is perhaps unfortunate, as it is the most likely determinant of any coculturing in the lab that there are strong ph shifts that affect which strains grow. Major ph shifts caused by the microbes are probably much less likely in the host as reflected by the normally consistent ph in the healthy gut. The authors argue that there results are likely to reflective of processes in the gut, from the correlation between c diff infection and high ph in patients. However, we do not know if the ph shift is a cause or a consequence in such data. I worry then that the strong effects of ph that drive a lot of the results do not reflect variation that is seen in the gut proper, and I think this should be at least acknowledged.
4. I agree that the effects of C. hiranonis could be resource competition but I did not see data to confirm it is not weak inhibition by an antimicrobial, such as the tryptophan based antibiotics discussed in the C. diff literature. And I do not see how a phylogenetic analysis can inform on this, given that there is often more interference competition between closely related species too. However, I am hopeful that experiments that add back concentrate of the growth media to the spent media can better inform on this.
5. I am also worried that the effects of glucose supplementation do not show for sure that glucose limitation is why C. diff grow poorly in the communities where this is studied. What I think can be concluded is that there is some factor causing growth limitation that glucose is able to alleviate, but this does not mean that low glucose is causal in the growth limitation. For example, could it be limitation of a second carbon source, or the effect of another compound that glucose is able to compensate for, or could it be that glucose is a signal that derepresses uptake systems for a secondary nutrient? I may have missed some piece of the argument here, but I need more convincing of the conclusions in this section, or I would suggest they are qualified by these concerns.

Reviewer #2:

In this study, Hromada et al. perform growth assays of single gut microbes and defined synthetic communities to predict how the community context influences the abundance of C. difficile in vitro. The authors find that increasing numbers of species decrease the abundance of C. difficile in test tubes on the one hand, whereas increasing inoculum size of C. difficile results in higher final C. difficile ratios on the other side. The authors conclude that the molecular mechanisms for the observed suppression of C. difficile by other gut bacteria is acidification of the culture media caused by fermentation reactions of fast growing bacteria and competition for glucose.

I found the addressed question interesting, and I believe that quantification of gut microbial growth is essential to better understand microbiota assembly and function. Although the authors have performed an impressive number of growth assays to feed the generalized Lotka-Volterra model, the findings seem incremental to their previous study and the generated molecular insights into bacterial community interactions with C. difficile remain very limited and unspecific. The authors have previously published a study in MSB, in which they described the community interactions with the same experimental and computational approach using the same species (without C. difficile and C. scindens). Also, in this previous work the authors found that decreased pH through bacterial growth affected the community composition.
Several findings of the current manuscript seem trivial from a bacteriological point of view and expected from simple growth kinetics under controlled in vitro batch culture conditions. For example, if a slow-growing species (i.e., C. difficile) and a fast-growing species (i.e., Bacteriodes ssp.) are mixed together, the fast one will naturally outcompete the slower one, if substrates are (partially) shared. Also, the fact that the ratio of two competing species can be altered by changing the inoculum size of either strain is expected, as their biomass generation exponentially depends on the initial number of cells (2^n). Hence, these two basic bacteriological principles seem to underly the main claims of the manuscript stating that increasing community richness has a negative impact on C. difficile community abundance.
Overall, the generated systematic growth data of gut bacteria and their synthetic communities will be of value for the scientific community. However, the molecular insights generated seem to be rather limited for publication in Molecular Systems Biology.

Major Comments.
1) The authors claim that their results demonstrate that species richness is a key determinant of C. difficile growth across a wide range of ecological contexts. The fact that several other strains of the assembled 13-membered community are outcompeted in the in vitro growth assays raises the question about how specific these findings are for C. difficile and to which degree these observations are due to the simplified experimental setup. Also, the range of 'ecological contexts' tested seems rather limited

under the given in vitro growth conditions.

2) The finding that growth media acidification of fast (in vitro) growing Bacteroides species negatively effects other community strains seems very general lacking C. difficile specificity and as such, has been investigated by the authors and others before. The degree of media acidification is highly dependent on the growth media used (e.g., available fermentative substrates and buffering capacity) and the showed data do not allow to assess how sensitive the results are on the specific experimental setup.

3) The authors suggest glucose competition between C. hieranonis and C. difficile, based on the fact that glucose was consumed in culture supernatant of C. hieranonis, C. difficile, and CommE and CommO after 20 hours of cultivation. Although interesting, glucose is a preferred carbon source of many (gut) bacteria and the authors should compare glucose utilization across additional conditions (stains and communities) to demonstrate that competition for glucose is specific to communities comprising C. hieranonis. Further, the used ABB media is complex containing large amounts of peptone and yeast extract, with glucose being mass-wise a minor ingredient. Therefore, substrate competition should be looked at in a broader context, as the authors did in their previous study using exometabolomics measurements and quantification of 97 major metabolites in culture media. Lastly, the in vivo relevance of glucose competition seems rather limited as the abundance of simple sugars, in particular glucose, in the large intestine is low.

Minor Comments.

4) The authors talk about bacterial growth and growth inhibition, often referring to maximal OD. The authors should be more precise throughout the manuscript and clearly distinguish between growth rates and maximal OD600/biomass. From a bacteriological point of view and with respect to community interactions, changes in either of the two can have fundamentally different effects. Whereas resource competition effects the biomass of individual species and the whole community, inhibition of replication might only affect growth rates, not changing the final biomass.

5) L118 - L124: The authors state that equal initial ratios result in higher rates of co-existence. Under the applied batch cultivation conditions leading to initial exponential growth this observation seems natural. However, it raises the question of the stable co-cultivation experiments shown in Fig. 1e. As the ratio between C. difficile and the paired strains change in the first batch cultures of most pairs, it remains unclear why the final OD of most strains remain constant in all three serial batch cultures. Several of them seem to be clear misfits between experimental and simulated data (e.g., FP, CH, SC, PC in co-culture with C. difficile). The authors should explain this observation and also provide larger figures to better compare experimental data and fitted lines.

6) L136: The authors claim that C. hieranonis reduced growth of C. difficile in the first passage to 78%. It is unclear whether this reduction is real or a measurement error, as in later passages the OD600 of C. difficile seems to be comparable to the one of the C. difficile monoculture (Fig 1d).

7) It is unclear why single species and paired cultures in Fig. 1 were cultured for 26 hours, whereas other communities were cultured for 48 hours. This raises the question how the data of the single and paired cultures were extrapolated for integration in community models. L253 - L261: The authors perform simulation for cultures between 48 and 96 hours. It is unclear, which experimental data feed into these simulations. Further, what does 'steady state' mean in a batch culture that typically reaches maximal OD in the first 24 hours of cultivation followed mainly by survival, maintenance, sporulation of strains, but less replication and growth.

8) L156-157: The authors talk about ecological niches. In the applied in vitro setup (test tubes) it seems artificial to talk about different ecological niches given homogeneity of liquid culture media. Different substrate specificity of different strains would seem a more accurate description.

9) The authors describe the effect of the 13-membered community on C. difficile community abundance. However, Fig. 2e shows that upon assembly of all 13 species, several species get lost (i.e., CH, ER, PC...). This should be taken into account and the community should be described as an X-membered community (with X describing the actual number of community members that are not outcompeted). Given the slow growth rates of these other species that go extinct in the community context, the results for the also slow growing C. difficile does not seem very surprising and should be discussed.

10) L190-196: The authors claim that C. difficile displays a stronger dependence on species richness than any other species in the system. The basis for this statement remains unclear and would need quantitative measures. Other species (e.g., PC and BU) undergo more dramatic changes upon increased community complexity than C. difficile, when also taking maximal abundance of these species into account.

11) Fig. 3. It is unclear, how Fig. 3b relates to Fig. 3a. This should be made clearer. Also, the observed saturation of the C. difficile ratio at increasing inoculum size is interesting. The authors should explore this further, as it could provide unexpected insights into C. difficile inhibition by community members.

12) L286: Fig. 3d should be referenced instead of Fig. 3c.

Reviewer #3:

Understanding Clostridioides difficile ecology in the gut microbiome is a current scientific challenge with important consequences to biomedicine and microbiome interventions. When this opportunistic pathogen takes over the gut microbiome of susceptible hosts, the resultant infection can be very resistant to antibiotic treatments. Fecal transplants, in which the compromised gut microbiome is largely replaced by the one from a healthy individual, have become the standard, effective practice to treat C. difficile infections, yet the mechanisms of action that prevent C. difficile to invade the new community are still largely unexplored.

The authors address this question through a combination of invasion experiments in vitro, which they support and complement with the predictions of an ecological model for C. difficile interactions with 13 representative species of the human gut microbiome. In particular, they ask the question of how C. difficile invasion outcome depends on the richness of the resident synthetic community. While this question is important to a broad audience, I believe that the manuscript needs a significant revision to better communicate the main take-aways of each experiment in a clearer way.

MAJOR CONCERNS AND SUGGESTIONS

1) I found the paper to be surprisingly difficult to read given how close my group's research is to the topic. Part of the difficulty was I think that there were many details presented in the main text that require a lot of provisos / interpretation that obscured somewhat the primary take-aways. Hopefully if some of the points below are clarified it will help to streamline the presentation for readers such as us (and hopefully not cause problems for other readers...).

2) Throughout the paper I was often left wondering which observations / differences in conditions were due to differences in the steady state community as compared to transient dynamics as the community approaches steady state. For example, even in the pair-wise co-cultures the authors observed some (modest) differences in the fractions after co-culture depending upon the starting fractions. Do the authors have any evidence that this is due to bistability or is it simply due to the co-cultures starting from further away from the final, steady-state fractions? If it is the latter then I am hesitant to make too big of a deal of the observation. This may be particularly difficult to disentangle given that the fractions were measured after just 24 hr (one cycle, at line 119). In any case, there will always be a dependence on the initial fraction after just one cycle... More generally, when we talk about coexistence we are referring to coexistence at steady state rather than after just one cycle, but I acknowledge that perhaps that is not the only way to do it.

Also, in the context of communities I would have appreciated knowing whether there is multi-stability in the model (or again if the dependence on initial conditions is simply due to transients). I understand that this is difficult to establish with confidence in the experimental communities, but it would at least be good to be clear about what is happening in the model.

Given these issues, it is a strong assumption that the abundances after one or two cycles are representative of the 'assembled community', which is understood as the long-term, stable community composition. It is very possible that after 48hr we are seeing community compositions that are still transient, significantly different from the equilibrium composition, especially for richer communities. This should be acknowledged in the text.

3) I also felt that the main result of the paper-which is captured in the title-was not addressed in a very straightforward way. To me, the question to be addressed is: in order to successfully invade, is it best for CD to compete against a small number of species or against a large number of them? Ideally, the community being invaded would have already reached equilibrium / steady-state over multiple growth dilution cycles, but I understand that this could be debated (although perhaps it would have also made the presentation / interpretation of results simpler). Of course, the experiments have already been done and in the end are I think sufficient to make the claim.

MINOR SUGGESTIONS

I strongly recommend an effort to convey the methods more clearly. In some cases, this might mean to include more details in the figure captions and results section. I found the paper difficult to read in this aspect, and I often had to go back and forth multiple times between the results section and the methods to understand how a given experiment was performed.

Specifically, how the absolute abundance of each species is calculated is not clear, and should be at least clearly stated that it is explained in the Method Data Analysis section. Preferably, it should be mentioned either in the text of the figure legend that the Absolute abundance was calculated by multiplying the relative abundance of an organism (according to the 16S-sequencing) by the OD600 of the sample. Then, a discussion like suggested later should be presented, as this way of estimating the total abundances of each species relies on some very serious assumptions.

Line 115: How many cycles were running? It seems like 3 but I would clearly state in the text

Line 119 & Figure 1d&e: Is it always CD that is going extinct? It seems the FP is extinct in the co-culture but also as a monoculture. But it would be nice to know where the threshold for extinction goes- maybe add this to the figures? In many of the plots the experimental data does not agree with the model (CD and PC for example), so it may be good to mention here that the Full Model includes fits to the multi-species communities.

Fig S2: How would different starting abundances of CD impact its abundance at 48hr simulation. Would it change the time till steady state reached?

Fig 3b: it would be helpful to have an indication of the initial richness of each community. It is a bit hard to get the richness from 3C, and Comm Q does not appear there.

Line 203-204. I am not sure that I agree with this interpretation, since all species do worse in more rich communities.

Line 251: are these results shown anywhere?

Lines 253-261 (Fig S5): - this simulation does not seem to agree with the data presented on Fig 3b. Can the authors provide an explanation for this? Also, doesn't this figure suggest the experimental data should be taken from longer times than 48hr? Though most of the plots seem independent of the initial inoculum at this time, some do.

Somewhere in the main text, a discussion on the estimated reliability on the measurements of CD abundance (OD), including factors that might affect the results. For example: are cells from all species considered to contribute equally to OD? This is only one additional source of uncertainty added to the many that 16S sequencing already has, but I believe that the discussion is worthwhile.

How are the error bars computed in Fig 4A?

Figure 5: It would be nice to discuss why adjusting both pH and Glucose in Community E give significantly more inhibition than adjusting just glucose. Given that adjusting pH only does not have an impact, this result is surprising.

**Reviewer #1:**

This is an interesting study that combines ecological modeling with simple in vitro experiments to study colonization resistance to C difficile. The authors show that diversity from the ecological side, and pH from the mechanistic side are important in their assay. In addition, they find evidence that adding back glucose to cultures can restore C. diff growth in some cases. I am broadly supportive of publication subject to a few questions/concerns being addressed:

1A. As I understand it, the authors use 16s to estimate frequencies of their frequencies which are then multiplied by OD to get absolute abundance. 16s is well known to be subject to significant biases that mean that the frequencies measured do not reflect the actual frequencies of a strain of bacteria in a sample. Were these biases estimated with a test community of known frequencies? Sorry if I missed this, but this issue is worth discussing prominently I think. Also, OD is well known to often fail to reflect cell numbers, particularly at higher densities. Again, did you do any tests to make sure this is not affecting your measurements and estimates, and conclusions?

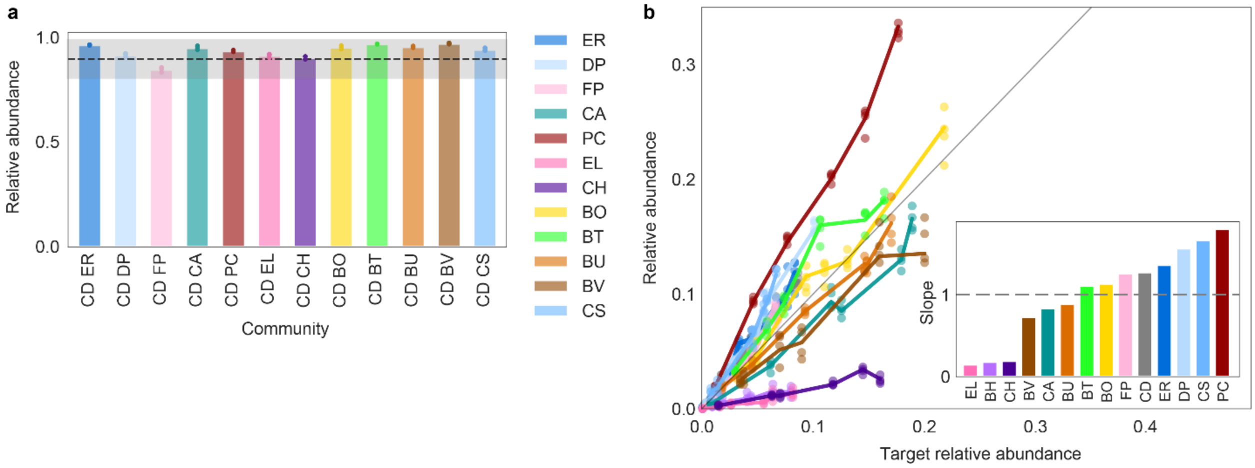


**Figure R1. 16S relative abundance of two-member and fourteen-member mixed cultures.** (a) Barplot of 16S relative abundance of non-*C. difficile* species in each two-member mixed community. Mixed cultures were assembled in 10:90 *C.difficile*:non-*C. difficile* species ratio based on OD600 measurements and collected immediately for sequencing. Dashed line indicates target relative abundance (0.9) of non-*C. difficile* species. Shaded region represents 10% error of target value. (b) Lineplot of 16S relative abundance of each species of the fourteen-member mixed culture versus the target relative fraction from OD600-based assembly across six mixed cultures. Each of the six mixed cultures contained all fourteen species but varied in the relative fractions. Datapoints represent four technical replicates. Line represents average of four technical replicates. Gray line is y=x. Inset: slope of linear regression for each species. Dashed line of slope = 1 represents perfect match of measured to target relative abundance.

The reviewer is correct that absolute abundance is calculated by multiplying the relative abundance from 16S sequencing by OD600. We have added a sentence in the Results section indicating how the absolute abundance is calculated (lines 102-104) and clarified this in the figures and figure legends using “Calculated OD600” to refer to the product of the relative abundance fraction and OD600 measurement.

We investigated 16S bias in twelve representative 2-member mixed cultures containing CD (**Fig. R1a**). We created communities with 90% species 1 and 10% species 2 (based on OD600 measurements) and then measured the relative abundance by 16S sequencing. The 16S relative

abundances for species 1 ranged from 85% to 97%, within 10% of the true value of 90%, giving us confidence in our ability to accurately quantify the relative abundance of 2-member communities containing CD. Note that our gLV model was trained on primarily lower species richness communities including pairwise communities with CD and thus it is likely that minimal bias was introduced using these data (**Fig. 1e**).

We also investigated 16S bias in 14-member mixed cultures (**Fig. R1b**). We created six different mixed cultures with various compositions and then measured the relative abundance by 16S sequencing immediately after mixing (no growth). We observed a linear relationship between expected OD600 and calculated OD600 for all species, indicating that we can observe variation in species abundance over a wide range and changes in expected OD600 were proportional to calculated OD600 (no saturation). Although most species were near the x=y line (low bias), CH, BH, and EL were underrepresented based on expected OD600. Interestingly, there was no such underrepresentation of these species in the CD-CH, CD-BH, or CD-EL pairs (**Fig. R1a**). This suggests 16S bias may change depending on the community context. As such, we do not apply any bias-correction for species with substantial bias based on Fig. R1b since the bias factor could vary across different community contexts.

There are two points that make us confident in our biological conclusions about microbial interactions in the manuscript despite inevitable biases in 16S sequencing that impacts every microbiome sequencing study based on 16S sequencing:

1) We confirm results obtained with 16S sequencing with conditioned media experiments. The inhibition of CD by CommI, CommH, CommK observed via 16S sequencing (**Fig. 4d**) was confirmed with conditioned media experiments in **Fig. 4e**. The inhibition of CD by CH in CD-CH, CommE, and CommO observed via 16S sequencing (**Fig. 5b**) was confirmed in conditioned media experiments (**Appendix Fig. S6**). Previous work using this 16S method found that 75% of interactions determined using 16S sequencing were in qualitative agreement with conditioned media experiments (Fig. S21 in PMID 29930200). It is possible that some of conditions that showed qualitative disagreement could be due to 16S bias. However, that is an overestimate, since there are notable differences between direct species interactions in community vs indirect interaction in conditioned media experiments (e.g. changed metabolite utilization in the presence of a second species) which contribute to the discrepancy.
2) We found a linear relationship between the true value and calculated 16S relative abundance for all species, even including the underrepresented set (**Fig. R1b**). This means that even in cases where the absolute abundance is biased, the fold change in species absolute abundance between conditions is accurate. In the manuscript, our conclusions are based on changes in species abundance between conditions and thus our conclusions are not based on a single condition. For example, we look at the trend of decreasing CD with increasing richness, trend of increasing CD with increasing propagule pressure, and trend decreasing CD with increasing CH. These trends would be robust to under- or over-representation due to the linear relationships in **Fig. R1b**.

To reduce OD600 error that occurs at high densities, we dilute our cultures into the linear range of our instrument for all OD600 measurements and then multiply by the dilution factor. We assume that cells from all species contribute equally to OD600. The alternative to this assumption is to measure the CFU per mL per OD (CFU $mL^{-1}$ $OD^{-1}$) for each species by CFU counting and apply a correction factor to normalize species with that feature a higher or lower CFU $mL^{-1}$ $OD^{-1}$. Unfortunately, CFU counting has its own biases due to selection pressure of solid media, cell adhesion which undercounts cell clumps as single colonies, and significant variation across

growth stages. Therefore, we use the simple assumption that cells from all species contribute equally to OD600. Any bias from this assumption should apply equally across all of the experiments, and the fold change between conditions would remain unaffected. Therefore, as discussed in point 2 above, our conclusions remain unaffected.

We have included a discussion of these assumptions and biases in our methods in the Discussion and new **Appendix Figure S7**.

1B. I thought figure 2e and the analyses that are behind it are great. However, I wasn't sure about the conclusion that C. diff has a unique profile therein. PC, CH, ER all also look like they have a strong negative dependence in the full simulated dataset. It comes on at higher diversities admittedly, but it looks like it is there. I wasn't sure why the authors then concluded that this dependence wasn't seen outside of C. diff.

We thank the author for pointing out this lack of clarity. We have now fit an exponential decay model to the data and simulations to quantify the dependence on richness for each species (**Fig. 2e**) and have displayed the decay constant *b* for each species (**Fig. 2f** data**, Fig. EV2** simulation). In addition, we carried out new community experiments to increase the number of measurements of all non-CD species in our dataset using randomly designed communities (**Fig. 2e**). Using this quantification, we can classify species as strongly dependent on richness ($b > 0.2$ in both data and simulation: CD, CH) and species with no dependence on richness ($b < 0.05$ in both data and simulation: BH, EL). This illustrates that *C. difficile* is not unique in its dependence on richness. However, the dependence on richness is also not universal across species in our community. Therefore, we consider this finding to be non-trivial and potentially of value in designing bacterial therapeutics. We have modified the text to replace descriptions of *C. difficile* having a unique relationship with richness with descriptions of *C. difficile* being in a unique class of species that exhibit a strong negative relationship with richness.

1C. The strong effect of pH in the assay is perhaps unfortunate, as it is the most likely determinant of any coculturing in the lab that there are strong ph shifts that affect which strains grow. Major ph shifts caused by the microbes are probably much less likely in the host as reflected by the normally consistent ph in the healthy gut. The authors argue that there results are likely to reflective of processes in the gut, from the correlation between c diff infection and high ph in patients. However, we do not know if the ph shift is a cause or a consequence in such data. I worry then that the strong effects of ph that drive a lot of the results do not reflect variation that is seen in the gut proper, and I think this should be at least acknowledged.

We argue that our pH results are potentially relevant in the human gut because the changes in pH that we observe in our *in vitro* measurements are within the variation seen in the colon, which has been shown to be highly variable, fluctuating between pH 5 and pH 8 (PMID 25411065). These changes are partially driven by production of acidic fermentation products by colonic bacteria, which is the same mechanism behind the acidification in our *in vitro* set up. Lastly, the buffering capacity of our media (4.8 mM bicarbonate) is similar to the buffering capacity in the colon (2-20mM bicarbonate, PMID 31989335).

The reviewer is correct that we do not know if the pH shift seen in the human cohort study is a cause or consequence of CDI. We thank the reviewer for this observation and have added this qualification to the Discussion.

1D. I agree that the effects of C. hiranonis could be resource competition but I did not see data to confirm it is not weak inhibition by an antimicrobial, such as the tryptophan based antibiotics

discussed in the C. diff literature. And I do not see how a phylogenetic analysis can inform on this, given that there is often more interference competition between closely related species too. However, I am hopeful that experiments that add back concentrate of the growth media to the spent media can better inform on this.

We thank the reviewer for pointing out that closely related species experience interference competition. We agree that phylogenetic analysis alone cannot inform on this, and have removed the phylogenetic analysis from our reasoning in the manuscript.

As the reviewer suggested, we performed an experiment supplementing concentrated media to *C. hiranonis* spent media. We find that adding media concentrate increases *C. difficile* growth to only 36% of its fresh media growth, indicating there is an additional inhibitory mechanism (**Fig. 5e**). We tested for protein-dependent inhibition via heat treatment and Proteinase K treatment of the supernatant and found no relief of inhibition, suggesting this unknown inhibitory mechanism that is not mediated by a protein. We have added these findings to the Results section. We have also added text to the Discussion section speculating that the additional mechanism by *C. hiranonis* could be from production of tryptophan-based antibiotics.

1E. I am also worried that the effects of glucose supplementation do not show for sure that glucose limitation is why C. diff grow poorly in the communities where this is studied. What I think can be concluded is that there is some factor causing growth limitation that glucose is able to alleviate, but this does not mean that low glucose is causal in the growth limitation. For example, could it be limitation of a second carbon source, or the effect of another compound that glucose is able to compensate for, or could it be that glucose is a signal that derepresses uptake systems for a secondary nutrient? I may have missed some piece of the argument here, but I need more convincing of the conclusions in this section, or I would suggest they are qualified by these concerns.

We agree with the reviewer. We cannot definitively conclude whether glucose is responsible for the growth limitation using our current methods. We modified our text to qualify these concerns in the results text (lines 428-429).

**Reviewer #2:**

2A In this study, Hromada et al. perform growth assays of single gut microbes and defined synthetic communities to predict how the community context influences the abundance of *C. difficile* in vitro. The authors find that increasing numbers of species decrease the abundance of C. difficile in test tubes on the one hand, whereas increasing inoculum size of C. difficile results in higher final C. difficile ratios on the other side. The authors conclude that the molecular mechanisms for the observed suppression of C. difficile by other gut bacteria is acidification of the culture media caused by fermentation reactions of fast growing bacteria and competition for glucose.

The reviewer is correct that we show that the abundance of *C. difficile* decreases with species richness (**Fig. 2e**) and increasing initial *C. difficile* density increases the abundance of *C. difficile* (**Fig. 3a**,b). However, while we show that modification of environmental pH is a major mechanism of inhibition by several communities (e.g. CommI, CommH, CommK and CommE), our results indicate that many of the tested communities interact with *C. difficile* via other mechanisms beyond pH. For example:

- *C. difficile* was inhibited by *C. hiranonis* via a pH-independent mechanism (**Fig. 5**).

- *C. difficile* growth was inhibited by CommO (**Fig. 4d**) but the environmental pH was similar to the pH of fresh media. In addition, pH adjusting the conditioned media of CommO did not enhance the growth of *C. difficile*, indicating that pH was not a major factor determining *C. difficile* growth in CommO conditioned media (**Fig. 4f**).
- The abundance of *C. difficile* varied substantially in communities where the environmental pH was similar to the value of fresh media (**Fig. 4d**), indicating that other mechanisms beyond pH influenced the growth of *C. difficile*. For example, *C. difficile* grew to an OD600 of 0.32 in CommJ whereas *C. difficile*'s abundance was 0.09 in CommG but their pH was similar, indicating the inhibitory effect of CommG was not driven by environmental pH modification.

Therefore, the interactions between sub-communities and *C. difficile* are complex and can involve other mechanisms beyond pH modification. We updated our manuscript to highlight that pH modification is not the only mechanism of interaction between the synthetic communities and *C. difficile*.

I found the addressed question interesting, and I believe that quantification of gut microbial growth is essential to better understand microbiota assembly and function. Although the authors have performed an impressive number of growth assays to feed the generalized Lotka-Volterra model, the findings seem incremental to their previous study and the generated molecular insights into bacterial community interactions with C. difficile remain very limited and unspecific. The authors have previously published a study in MSB, in which they described the community interactions with the same experimental and computational approach using the same species (without C. difficile and C. scindens). Also, in this previous work the authors found that decreased pH through bacterial growth affected the community composition.

We thank the reviewer for asking for clarification about the main novel results of our paper. Understanding the ecological and molecular interactions influencing pathogen growth can guide the design of microbiome interventions that inhibit the growth of the pathogen. Current approaches for selecting species for defined bacterial therapeutics are not guided by ecological and molecular principles. Therefore, a deeper understanding of these mechanisms of inhibition between commensal gut species and *C. difficile* could guide the selection of optimal combinations of species that inhibit *C. difficile* by exploiting known mechanisms of interaction. For instance, if resource competition was a major mechanism of inhibition, human gut bacterial isolate collections could be screened for species that efficiently utilize key resources required for *C. difficile* growth. Next, isolates with the desired phenotypes could be combined into a bacterial therapeutic that is designed to inhibit *C. difficile* via resource competition. To work towards this future goal, we were motivated to investigate the ecological and molecular interactions between a synthetic gut community and *C. difficile*.

While our paper used similar methods based on the generalized Lotka-Volterra model to Venturelli OS et al. *Mol. Syst. Biol.* 2018, the biological questions in these two papers are very different. In Venturelli OS et al. *Mol. Syst. Biol.* 2018, we sought to understand whether higher-order community assembly could be predicted using a gLV model that was parameterized based on time-series measurements of lower-order (monospecies and pairwise) communities. In this study, we leveraged a similar method to investigate ecological principles shaping *C. difficile* growth in synthetic communities. However, our goal was to understand the ecological principles influencing *C. difficile* growth to guide the design of new therapeutic approaches to inhibit *C. difficile* in the future. Below we summarize the major novel findings of our paper:

- Using our model, we demonstrate that *C. difficile* uniquely has the highest number and largest magnitude negative incoming interspecies interactions (**Fig. 2b,d**). This strong inhibition based on the gLV model is consistent with the strong negative relationship between community richness and *C. difficile* abundance.
- We quantified the relationship between community richness and the abundance of each species in our data and in all possible communities by simulating our model. Our results highlight *C. difficile, C. hiranonis* and *P. copri* are three unique species in the community with highest magnitude sensitivity to community richness (**Fig. 2e,f**). By contrast, we identified a set of species with low sensitivity to community richness (e.g. *B. hydrogenotrophica*, *B. vulgatus, etc)* and intermediate sensitivity to community richness (e.g. *E. rectale, B. ovatus, B. uniformis*, etc). The sensitivity to richness has not be characterized before and may be an important property in natural environments. Future work will investigate the effects of this property on community stability and response to perturbations. We show that the strong dependence of *C. hiranonis* and *C. difficile* on species richness is consistent with the large number of incoming negative interactions in the gLV model.
- Guided by our gLV model, we show that a close relative *C. hiranonis* in this community strongly inhibits *C. difficile* (**Fig. 5b**). We investigated the molecular basis of this interaction. After ruling out toxicity due to changes in environmental pH as a mechanism of interaction, we hypothesized that these species share ecological niches and may compete for limited resources. To test this, we carried out exo-metabolomic profiling of *C. hiranonis* and *C. difficile* and found that seven nutrients were depleted in each of their conditioned medias, suggesting the possibility that they could be competing for these limiting nutrients. We next designed an experiment to determine the contribution of these metabolites to the growth of *C. difficile* in *C. hiranonis* conditioned media. To this end, we measured the concentrations of the seven nutrients in *C. hiranonis* conditioned media and supplemented the conditioned media of *C. hiranonis* with each of these nutrients individually, all combinations of six nutrients or all seven nutrients. Our results indicate that only glucose supplemented individually resulted in a statistically significant increase in the growth of *C. difficile*. In addition, mixtures of the six nutrients resulted in similar growth enhancements, suggesting metabolic redundancies in these nutrients for growth. However, none of these conditions completely restored the growth of *C. difficile.* Further, the addition of high concentrations of other nutrients (concentrated media supplementation) did not fully rescue the growth of *C. difficile*. Therefore, the inhibitory interaction of *C. hiranonis* on *C. difficile* is complex and consists of a resource competition component and an unknown non-resource competition. Future experiments can investigate the molecular basis of the resource competition-independent component of the interaction between *C. hiranonis* and *C. difficile*. These results highlight the complexity of microbial interactions which can consist of an interplay of different mechanisms that combine to generate a net effect.
- We explored the role of initial inoculum density on *C. difficile* growth in synthetic communities. To our knowledge, the effect of dosage of *C. difficile* on invasion success has not been studied *in vitro* or *in vivo*. It remains an open question if increased dosage of *C. difficile* leads to higher likelihood of infection in humans Here we present a simple *in vitro* study as a first step to address this question. We find that over a 48 hour period, the abundance of *C. difficile* increases with initial inoculum density (**Fig. 3b**). This suggests that dosage of *C. difficile* could be an important factor for infection and is worth further investigation. We note in this

experiment the maximal *C. difficile* abundance as a function of propagule pressure is still dictated by community context (**Fig. 3b**), as is the sensitivity (defined by the abundance of *C. difficile* corresponding to the half-maximal abundance in the assembled community). This suggests that while lowering the dosage/exposure of *C. difficile* could be important for preventing infection, the composition of resident gut community also plays a significant role. We think the response curves properties of sensitivity and maximal *C. difficile* abundance (**Fig. 3b**) may be useful for the design and optimization of defined bacterial therapeutics since the initial inoculum density of *C. difficile* is not known and hard to predict in the environment.

Several findings of the current manuscript seem trivial from a bacteriological point of view and expected from simple growth kinetics under controlled in vitro batch culture conditions. For example, if a slow-growing species (i.e., C. difficile) and a fast-growing species (i.e., Bacteriodes ssp.) are mixed together, the fast one will naturally outcompete the slower one, if substrates are (partially) shared.

Predicting the composition dynamics of microbial communities and microbiomes is challenging due to the complex web of interactions among constituent community members and currently an unsolved problem (PMIDs 29930200, 34098512, 34059668). Indeed, “simple bacteriological principles” of resource competition between species that utilize a shared pool of resources are usually unable to predict the dynamics of microbial communities. For example, in our study, we show that the growth rate of *C. difficile* is 75% of the growth rate of fast growing *Bacteroides* yet *C. difficile* was able to coexist with the fast-growing species over multiple passages (e.g. pairwise communities CD, BV; CD, BU; CD, BO) (**Fig. 1d,e**). In addition, 24% of the inferred inter-species interaction parameters of our gLV model were positive, suggesting potential mechanisms of detoxification of the environment (e.g. pH buffering) or release of nutrients that enhances the growth of constituent community members (**Fig. 2b**). If competition over limiting nutrients was the only mechanism shaping the assembly of the community, then our inferred gLV interaction network consist of only negative interactions, which is not the case. Therefore, our results indicate that more complex interactions beyond resource competition are shaping the assembly of the synthetic gut community.

Also, the fact that the ratio of two competing species can be altered by changing the inoculum size of either strain is expected, as their biomass generation exponentially depends on the initial number of cells (2^n).

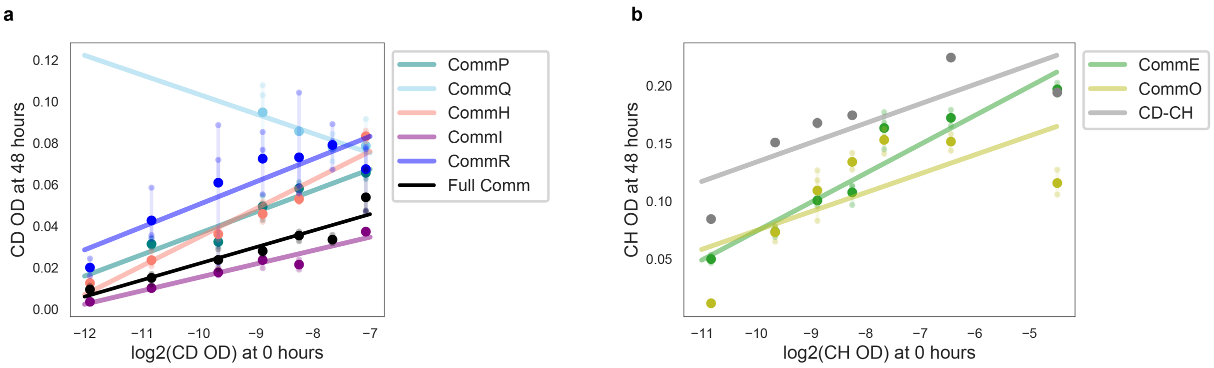

**Figure R2. Relationship between initial species abundance and final abundance in the assembled community at 48 hr.** Lineplots of calculated OD600 of (a) *C. difficile* and (b) *C. hiranonis* at 48 hours as a function of the log of their initial abundance. Large datapoints indicate average of 3 biological replicates, small datapoints indicate biological replicates and are connected to average datapoint with transparent lines. Lines indicate linear regression of average datapoints.

By varying the initial density of *C. difficile*, we show that higher initial density of *C. difficile* can result in higher abundance of *C. difficile* in the assembled community (**Fig. 3a**, inset). However, the abundance of *C. difficile* in the assembled community varies over a wide range (~0.05-0.4) when introduced at similar high initial densities into different sub-communities (**Fig. 3a**). Consistent with this, we show that different sub-communities exhibit different response curves to initial densities (i.e. different EC50s and maximum abundance at 48 hours in the assembled community) across a broad range of initial densities of *C. difficile* (**Fig. 3b**). Therefore, these data suggest that each community has unique interactions with *C. difficile* that determine its response to variations in initial cell densities. To further explore this possibility, we examined the linearity of the relationship between the log2 transform of the initial density and the species abundance in the assembled community at 48 hours for both *C. difficile* and *C. hiranonis* (**Fig. R2**). If there was an exponential relationship between the initial density of the species and the abundance in the assembled community, we would expect a linear relationship between the log2 transform of initial cell density and abundance in the assembled community. While a linear relationship was observed for certain communities (e.g. CommI and the Full Comm) (**Fig. R2a**), we observed a deviation from linearity for other communities (e.g. CommR in **Fig. R2a** and CommE, CommO and CD-CH in **Fig. R2b**). As such, not all species exhibit an exponential relationship with initial cell density depending on the community context. In addition, **Fig. R2a,b** show that the linear regression slopes of the lines can be substantially different across communities, which represents the degree of change in species abundance as a function of variation in the initial abundance. The slope is determined by the inter-species interaction network. Therefore, our data indicates that we need to consider inter-species interactions when predicting the abundance of species such as *C. difficile* in microbial communities. As such, simple bacteriological principles alone are insufficient to understand the growth of *C. difficile* in synthetic human gut communities.

Hence, these two basic bacteriological principles seem to underly the main claims of the manuscript stating that increasing community richness has a negative impact on C. difficile community abundance.

Our results show that the variation in the relationship between species abundance and species richness cannot be explained by simple bacteriological principles. If this were the case, then we would expect that fast-growing species (e.g. *Bacteroides*) would be less sensitive to community richness than slow-growing species (e.g. *E. lenta, D. piger, B. hydrogenotrophica*) (**Fig. 1d**). Fast-growing species would be able to effectively compete for limiting nutrients across a wider range of communities that vary in richness whereas slow-growing species would not be able to persist in higher richness communities since they are unable to compete for nutrients. However, we found that the abundance of fast-growing species such as *B. uniformis* does change substantially as a function of species richness whereas the abundance of slow-growing species such as *B. hydrogenotrophica, D. piger* and *E. lenta* did not vary significantly with species richness (**Fig. 2e**). We have now included an Appendix Figure showing that there is no correlation between the growth rate of species and the fitted decay constant quantifying the magnitude of the sensitivity of changes in abundance of each species as a function of species richness (**Fig. EV2b**). We

hypothesize that the variation in species abundance as a function of species richness is due to the dynamic interactions within the community and thus cannot be explained by simple bacteriological principles.

Overall, the generated systematic growth data of gut bacteria and their synthetic communities will be of value for the scientific community. However, the molecular insights generated seem to be rather limited for publication in Molecular Systems Biology.

We thank the reviewer for pointing out that our work will be of value to the scientific community. We have listed the novel contributions of our paper above and believe that our paper will be of broad interest to the microbial ecology and systems biology fields. During the revision, we carried out several new experiments that strengthen our paper's contributions to understanding ecological interactions influencing *C. difficile*. For instance, we demonstrate that *C. difficile* is within a unique class of species in the gut community which exhibits a strong negative dependence on the species richness. In addition, guided by our model, we identify a strong negative interaction between *C. hiranonis* and *C. difficile* and interrogate the molecular basis of this interaction using exo-metabolomics.

Major Comments.

2B: The authors claim that their results demonstrate that species richness is a key determinant of C. difficile growth across a wide range of ecological contexts. The fact that several other strains of the assembled 13-membered community are outcompeted in the in vitro growth assays raises the question about how specific these findings are for C. difficile and to which degree these observations are due to the simplified experimental setup. Also, the range of 'ecological contexts' tested seems rather limited under the given in vitro growth conditions.

We use the term 'ecological contexts' to indicate the many sub-communities tested. We test *C. difficile* growth in >120 communities featuring a range of species richness levels, a range of phylogenetic distances between species which span 4 phyla, and a range of mutualistic, competitive, and parasitic interactions. We have edited the text to change 'ecological contexts' to 'community contexts' to better communicate our intent.

The reviewer is correct that multiple strains are outcompeted in the 13-membered community and that this observation is not unique to *C. difficile*. While the inhibition of growth in high richness communities is not unique to *C. difficile*, this relationship is not universal to all species. We have now quantified the growth vs richness relationship in **Fig. 2ef** by fitting an exponential decay model. In addition, we carried out a new experiment to increase the number of measurements of all species excluding *C. difficile* using randomly designed communities. This quantification more clearly indicates that the strong inverse relationship with richness is not universal to all species. In both the simulations and data, *C. difficile* and *C. hiranonis* display a strong inverse relationship between growth and richness (decay constant $b>0.2$) while BH and EL display no relationship ($b<0.05$). Therefore, the inverse relationship between growth and richness is not simply a result of our setup. The relationship depends on species identity. We have modified the relevant text in the Results section.

2C: The finding that growth media acidification of fast (in vitro) growing Bacteroides species negatively effects other community strains seems very general lacking C. difficile specificity and as such, has been investigated by the authors and others before. The degree of media acidification is highly dependent on the growth media used (e.g., available fermentative substrates

and buffering capacity) and the showed data do not allow to assess how sensitive the results are on the specific experimental setup.

While inhibition by Bacteroides through media acidification has been demonstrated before, these results are relevant for our study which seeks to understand the variety of ways our resident species interact with *C. difficile*, one of which is pH. While this general mechanism is known, here we show how prevalent this mechanism is in a subset of synthetic gut communities and determine which interactions can be explained by pH. To test whether inhibition by pH depends on our growth media, we carried out an experiment that demonstrates that CommI acidifies the media and inhibits *C. difficile* in a pH-dependent manner in multiple medias (**Appendix Fig. S5**). These data demonstrate that pH inhibition was not specific to our one media.

2D: The authors suggest glucose competition between C. hieranonis and C. difficile, based on the fact that glucose was consumed in culture supernatant of C. hieranonis, C. difficile, and CommE and CommO after 20 hours of cultivation. Although interesting, glucose is a preferred carbon source of many (gut) bacteria and the authors should compare glucose utilization across additional conditions (stains and communities) to demonstrate that competition for glucose is specific to communities comprising C. hieranonis. Further, the used ABB media is complex containing large amounts of peptone and yeast extract, with glucose being mass-wise a minor ingredient. Therefore, substrate competition should be looked at in a broader context, as the authors did in their previous study using exometabolomics measurements and quantification of 97 major metabolites in culture media. Lastly, the in vivo relevance of glucose competition seems rather limited as the abundance of simple sugars, in particular glucose, in the large intestine is low.

As suggested by the reviewer, we investigated substrate competition in a broader context. We performed exometabolomics measurements of *C. hiranonis* and *C. difficile* monocultures and identified 7 compounds that were utilized by both *C. hiranonis* and *C. difficile* **(Fig. 5c)**: Acetyl-Ornithine, Glucose, Glutamine, Proline, Pyruvate, Serine and Threonine. We hypothesized that *C. hiranonis* and *C. difficile* compete over these metabolites.

Crucially, not only did we identify that these metabolites are consumed in both species, we also performed add-back experiments where we replenished these consumed metabolites *in C. hiranonis* supernatant. When we replenished all seven metabolites in the supernatant, we observed an increase in *C. difficile* growth compared to unmodified supernatant (45% of fresh media growth compared to 23%). See **Fig. 5d**.

- When we replenished just glucose*, C. difficile* grew significantly better (41% of fresh media growth) than in unmodified supernatant (23% of fresh media growth)
- There was no significant difference for any of the other six individual metabolite adjustments.
- Interestingly, there was no significant difference between *C. difficile* growth in the seven-compound supernatant and the six-compound supernatant missing D-glucose. Therefore, although the non-glucose metabolites don't significantly impact *C. difficile* growth when added individually, they do as a set.
- *C. difficile* grows equally in all of the six-compound supernatants, suggesting metabolic redundancy among this set of compounds.

In total, this data shows that replenishing the seven metabolites can partially relieve the observed *C. hiranonis*-*C. difficile* inhibition. This suggests that *C. difficile* is inhibited in *C. hiranonis* supernatant due to low concentration of some subset of these metabolites which were consumed by *C. hiranonis*. This is supported by our data that shows these metabolites are also consumed

by *C. difficile* in monoculture. However, as we note in the text, we cannot rule out that this effect could alternatively arise due to these metabolites compensating for the effects of different compound(s) that inhibits *C. difficile*.

As far as the in vivo relevance of glucose competition, Stephen AM *et al* (PMID 6873605) demonstrate that 20% of total glucose is unabsorbed in a glucose tolerance test in healthy adults (their Fig. 3B). In agreement, Anderwald C *et al* (PMID 21147888) find that 2-20% of carbohydrate is unabsorbed by the small intestine and makes it to colon. We believe that this reported amount of glucose in the colon makes glucose competition a relevant mechanism in vivo. We reference these studies in the Discussion for the reviewer and reader to consider.

Minor Comments

2E: The authors talk about bacterial growth and growth inhibition, often referring to maximal OD. The authors should be more precise throughout the manuscript and clearly distinguish between growth rates and maximal OD600/biomass. From a bacteriological point of view and with respect to community interactions, changes in either of the two can have fundamentally different effects. Whereas resource competition effects the biomass of individual species and the whole community, inhibition of replication might only affect growth rates, not changing the final biomass.

We have edited the manuscript to clarify between growth rates and maximal OD600. We have indicated “carrying capacity” or “abundance” when we are referring to maximal OD600 and indicated “growth rate” when referring to growth rate. Additionally, we added a line to explain that the area under the curve (AUC) of OD600 measurements over time encompasses both growth rate and maximal OD600 effects (line 346). Thus, when discussing AUC measurements, we use “growth” generally.

2F: L118 - L124: The authors state that equal initial ratios result in higher rates of co-existence. Under the applied batch cultivation conditions leading to initial exponential growth this observation seems natural. However, it raises the question of the stable co-cultivation experiments shown in Fig. 1e. As the ratio between C. difficile and the paired strains change in the first batch cultures of most pairs, it remains unclear why the final OD of most strains remain constant in all three serial batch cultures. Several of them seem to be clear misfits between experimental and simulated data (e.g., FP, CH, SC, PC in co-culture with C. difficile). The authors should explain this observation and also provide larger figures to better compare experimental data and fitted line

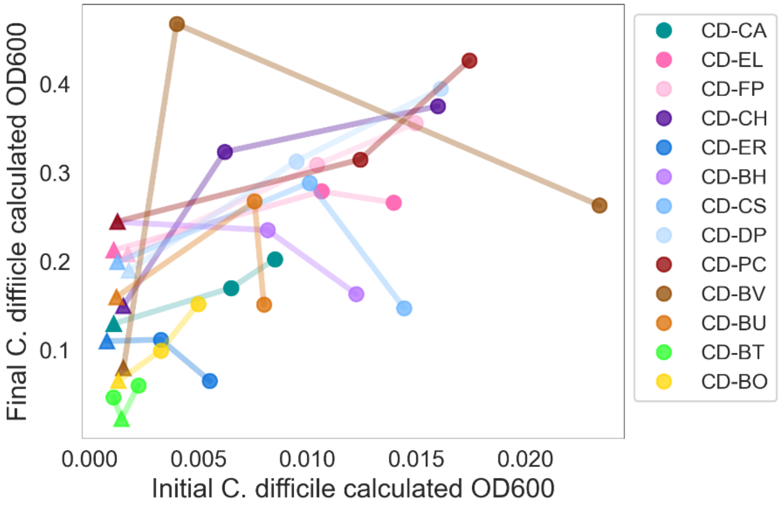


**Figure R3: *C. difficile* initial and final abundance in the three growth cycles of Fig. 1e**. Scatterplot of initial *C. difficile* calculated OD600 versus final *C. difficile* calculated OD600 in *C. diffiicle* pairs over three growth phases. Triangles indicate first growth phase, while circles indicate second and third growth phases.

The author is correct that the starting ratio between C. difficile and the paired strains changes between each growth phase in **Fig. 1e**. In some strains, the CD ratio is higher in later growth phases (e.g. FP, CS, PC), but in others, the CD ratio is lower in later growth phases (e.g. ER, BT, BO). However, regardless of if the ratio increases or decreases, the initial CD OD600 is always higher in growth phases 2 and 3 than growth phase 1 (except for growth phase 2 of CD-BT) (**Fig. R3**). Therefore, it is not surprising that CD final OD is higher in growth phases 2 and 3 than in growth phase 1 for the majority of the pairs (**Fig. R3**).

We provide 2 illustrative examples:

**CD-FP**

- The initial ratio of CD in CD-FP increases: 50%, 75% and 86% in the first, second, and third phases respectively
- The initial OD600 of CD in CD-FP increases: 0.0011, 0.010, 0.015 in the first, second, and third phases respectively
- The final OD600 of CD in CD-FP increases: 0.21, 0.31 and 0.36 in the first, second, and third phases respectively

**CD-BO**

- The initial ratio of CD in CD-BO decreases: 50%, 12%, and 17% in the first, second, and third phases respectively
- The initial OD600 of CD in CD-BO increases: 0.0011, 0.0033, and 0.0050 in the first, second, and third phases respectively.
- The final OD600 of CD in CD-BO increases: 0.07, 0.10, and 0.15 in the first, second, and third phases respectively.

In the CD-FP example, there is an increase in all three metrics across the three cycles: increase in initial ratio of CD, increase in initial OD600 of CD, and increase in final OD600 of CD. In the CD-BO example, while the initial ratio of CD decreases, there is still an overall increase in initial

OD600 and an increase in final OD600 of CD. This agrees with our findings in **Fig. 3a,b** that increasing initial CD abundance (OD600) increases its final abundance in the community.

We thank the reviewer for the suggestion to increase the size of the figures and have adjusted **Fig1d,e**. While the model fits well to the majority of our data (**Fig. EV1a**, goodness of fit r=0.89), it does not accurately describe all communities. We give three potential reasons for this lower goodness of fit:

1) The model is trained on multispecies data in addition to the pairs and monospecies data that we show fits for in **Fig. 1de**. Therefore, the parameter estimation procedure minimizes the total error between the model and the data (while penalizing the magnitude of the parameters) and thus balances goodness of fit to different datasets.
2) The gLV model has a single static parameter for the interspecies interaction between two species and a single static parameter for species growth rate, but in these growth cycle dilution experiments, the growth rate and interactions could be changing over time.
3) Variation between experiments due to biological or technical origins. For example, there is variation in growth between the two monospecies experiments M1 and M2 displayed in **Fig1d** (M1: three growth cycles, two measurements per cycle. M2: one growth cycle, 10 measurements per cycle). The two monospecies experiments vary in the number of timepoints taken and therefore the number of times the community was disturbed to take an aliquot for measurement, which could affect the growth of the organisms. The model fit balances fitting to both M1 and M2 monospecies experiments.

2G: L136: The authors claim that C. hieranonis reduced growth of C. difficile in the first passage to 78%. It is unclear whether this reduction is real or a measurement error, as in later passages the OD600 of C. difficile seems to be comparable to the one of the C. difficile monoculture (Fig. 1d).

*C. hiranonis* reduced the carrying capacity of *C. difficile* in the first passage in all 3 biological replicates, to an average of 78% of *C. difficile*'s monospecies carrying capacity (**Fig. 1e**). *C. hiranonis* also reduced the carrying capacity of *C. difficile* in the first passage in all 3 biological replicates when the initial ratio was 10% *C. difficile*: 90% *C. hiranonis* (**Appendix Fig. S1**) to an average of 58% of *C. difficile's* monospecies carrying capacity. We have no reason to believe these are measurement errors as the data and sequencing went through our quality control pipeline and the biological replicates are consistent. The fact that the carrying capacity of *C. difficile* in later growth cycles is unchanged from monospecies is likely due to there being no *C. hiranonis* present in these later growth cycles. In the experimental data, *C. hiranonis* did not grow in the second and third growth cycles in either **Fig. 1e** or **Appendix Fig. S1**. Without *C. hiranonis* present, it is not surprising that no inhibition is observed. We now mention this in results section of our manuscript.

2H: It is unclear why single species and paired cultures in Fig. 1 were cultured for 26 hours, whereas other communities were cultured for 48 hours. This raises the question how the data of the single and paired cultures were extrapolated for integration in community models. L253 - L261: The authors perform simulation for cultures between 48 and 96 hours. It is unclear, which experimental data feed into these simulations. Further, what does 'steady state' mean in a batch culture that typically reaches maximal OD in the first 24 hours of cultivation followed mainly by survival, maintenance, sporulation of strains, but less replication and growth.

We cultured single species and paired cultures for 26 hours as we observed in preliminary experiments that the cultures reached stationary phase by this time. We wanted to understand

the long-term behavior of these cultures. Since the cultures enter a state of maintenance, survival, and death during stationary phase, to understand the long-term behavior, passaging into fresh media was required. Therefore, we performed multiple batch culture cycles to learn about the long-term behavior of the monospecies and pairs.

We cultured multispecies communities for 48 hours in a single batch culture cycle. We wanted to study a large set of multispecies communities to best understand the features of communities that inhibit *C. difficile* growth. The rich dynamics in the first batch culture cycle, when the community is further from steady-state, is more informative for determining inter-species interactions than measurements closer to steady-state where the community is not changing as much as a function of time. Additionally, measuring single batch culture cycles is easier experimentally than multiple dilution cycles. Therefore, we focused our measurements primarily on the first batch culture cycle to provide a large and informative dataset to decipher inter-species interactions.

Regarding the specific 48 hour time point, we wanted to capture the communities close to early stationary phase because we wanted to study the carrying capacity of each organism in the communities. If we sampled in exponential phase, organisms may not have reached their carrying capacity yet, and if we sampled in late stationary phase, organisms may have reached a death phase. Because we sample a wide variety of communities, no one timepoint would be early stationary for all communities. We performed simulations with our Preliminary model and found that many communities were not at stationary phase by 26 hours, but the majority of the communities reached stationary phase by 48 hours. Therefore 48 hours was selected as the time point for our single growth phase experiments.

The gLV Full Model used for simulation was trained on the 26 hour passaged experimental data (**Fig. 1de, Appendix Fig. S1**) as well as 48 hour single growth phase multispecies experimental data (**Fig. 2a**e, **Fig. 3b**, **Fig. 4a**, **Fig. 5b**). See Table 1 in Methods. The resulting Full Model consists of best fit parameters for growth rate as well as interspecies interactions. The communities were simulated using these growth rate and interspecies interaction parameters. These parameters can be used to simulate the community composition at any time point and do not have to match the time points used to train the model.

We agree with the reviewer that the term ‘steady state’ is confusing in this setting. We were referring to the steady state in a simulation of the gLV model, where the steady state is defined as the timepoint where the community composition stops changing. However, this definition of steady state is less relevant biologically, since these steady state timepoints fall in late stationary phase, a time of maintenance, survival, and death. An alternative view of steady state is the long-term behavior of communities over multiple dilution cycles. We have revised the text to refer to “short timescales”, which we define in the text as a single batch cycle and “long timescales”, which we define in the text as multiple batch culture cycles.

2I: L156-157: The authors talk about ecological niches. In the applied in vitro setup (test tubes) it seems artificial to talk about different ecological niches given homogeneity of liquid culture media. Different substrate specificity of different strains would seem a more accurate description.

Ecological niche broadly refers to the multidimensional space of resources and environmental conditions that together define where a microbe can grow (definition based on Hutchinson *Cold Spring Harb Symp Quant Biol* 1957). Spatial heterogeneity is not required for establishing distinct niches. While our media is homogeneous, the rich media is multifaceted as it contains a variety of resources (sugars, amino acids, hydrogen, carbon dioxide, etc) that can support the growth of multiple strains with different ecological niches. In addition, microbes can release

nutrients into the environment that can be utilized by other constituent community members and thus create new ecological niches by engineering their environment. Because of the multifaceted nature of the media and the creation of new ecological niches by the community, we find referring to ecological niches helpful. For example, a hypothetical Community X with a sugar fermenter, an amino acid fermenter, and an acetogen that fixes carbon dioxide will have more ecological niches filled than Community Y with sugar fermenters alone. Theory holds that an invader may grow better in Community Y.

2J: The authors describe the effect of the 13-membered community on C. difficile community abundance. However, Fig. 2e shows that upon assembly of all 13 species, several species get lost (i.e., CH, ER, PC...). This should be taken into account and the community should be described as an X-membered community (with X describing the actual number of community members that are not outcompeted). Given the slow growth rates of these other species that go extinct in the community context, the results for the also slow growing C. difficile does not seem very surprising and should be discussed.

The number of species that are present changes over time. A species that is extinct at our measured timepoint of 48 hours could have been present and active from 0 to 47 hours and exhibiting an effect on *C. difficile* during this time. Thus, we choose to describe communities as X-membered, where X is the number of community members present at 0 hours.

We analyze if defining community richness as the species not extinct at 48 hours, as you suggest, would result in a different relationship between *C. difficile* growth and richness (**Appendix Fig. S3a**). We find the relationship holds whether initial richness (richness at 0 hours, **Fig. 2a**) or the species not extinct at 48 hours (richness at 48 hours, **Appendix Fig. S3a**) is used.

The species growth rates in our system range from 0.19 to 0.52 and *C. difficile* has a growth rate of 0.35, making it the 6$^{th}$ fastest grower out of 14 and not considered a slow-grower. Growth rate alone cannot predict species that go extinct in the full community. For example, *C. difficile* and *C. aerofaciens* have nearly identical growth rates (0.345 and 0.347), but drastically different abundance in the full community (**Appendix Fig. S3b**). This is because the growth rate of a species is modified by positive and negative interactions from other species in the community.

2K: L190-196: The authors claim that C. difficile displays a stronger dependence on species richness than any other species in the system. The basis for this statement remains unclear and would need quantitative measures. Other species (e.g., PC and BU) undergo more dramatic changes upon increased community complexity than C. difficile, when also taking maximal abundance of these species into account.

We thank the reviewer for the excellent suggestion to quantify these relationships. We have fit an exponential decay model to the experimental data and simulations (**Fig. 2e**) and used the decay constant to quantify the dependence of growth on richness for each species (**Fig. 2f**, **Fig. EV2a**). In the experimental data we find that *C. difficile*, CH, and PC display a strong inverse relationship between growth and richness (decay constant b>0.2) while BH, BV, DP, and EL display no relationship (b<0.05). The fit to the simulated data has *C. difficile*, CH, and ER having the strong inverse relationship (b>0.2), and *C. difficile* has a significantly higher decay constant than any other species (b=0.91). The exponential fits to our data and model show good agreement overall.

Taking the consensus of the experimental data and simulated data, we conclude that *C. difficile* has a strong inverse relationship between growth and richness and that this relationship is not universal across all species. We have modified the text to replace descriptions of *C. difficile* having

a unique relationship with richness with descriptions of *C. difficile* being in a unique class of species that exhibit a strong negative relationship with richness.

2L: Fig. 3. It is unclear, how Fig. 3b relates to Fig. 3a. This should be made clearer. Also, the observed saturation of the C. difficile ratio at increasing inoculum size is interesting. The authors should explore this further, as it could provide unexpected insights into C. difficile inhibition by community members.

We modified the text in the Results section to clarify the relationship between **Fig. 3a** and **Fig. 3b**. The experiment in **Fig. 3b** is similar to the experiment in **Fig. 3a** except that we increased the number of initial densities in order to better resolve the relationship between propagule pressure and *C. difficile* abundance. By doing this experiment, we were able to learn that the relationship between propagule pressure and *C. difficile* for each community is an increasing function that saturates. Additionally, we were able to determine that different communities feature different sensitivities (defined as the EC50 of the response curves in **Fig. 3b**). These findings were not possible with the limited data in **Fig 3a**.

We agree that the observed saturation is interesting! The differential saturating abundance observed in **Fig. 3b** reflects the different in the network of interactions present in different communities. These interspecies interactions alter *C. difficile* ability to grow in each community. We further investigate the inhibition by community members in CommH and CommI in **Fig. 4** and the interaction between *C. difficile* and CommR member *C. hiranonis* in **Fig. 5**.

2M: L286: Fig. 3d should be referenced instead of Fig. 3c.

This has been corrected.

**Reviewer #3:**

Understanding Clostridioides difficile ecology in the gut microbiome is a current scientific challenge with important consequences to biomedicine and microbiome interventions. When this opportunistic pathogen takes over the gut microbiome of susceptible hosts, the resultant infection can be very resistant to antibiotic treatments. Fecal transplants, in which the compromised gut microbiome is largely replaced by the one from a healthy individual, have become the standard, effective practice to treat C. difficile infections, yet the mechanisms of action that prevent C. difficile to invade the new community are still largely unexplored.

The authors address this question through a combination of invasion experiments in vitro, which they support and complement with the predictions of an ecological model for C. difficile interactions with 13 representative species of the human gut microbiome. In particular, they ask the question of how C. difficile invasion outcome depends on the richness of the resident synthetic community. While this question is important to a broad audience, I believe that the manuscript needs a significant revision to better communicate the main take-aways of each experiment in a clearer way.

MAJOR CONCERNS AND SUGGESTIONS

3A: I found the paper to be surprisingly difficult to read given how close my group's research is to the topic. Part of the difficulty was I think that there were many details presented in the main text that require a lot of provisos / interpretation that obscured somewhat the primary take-aways.

Hopefully if some of the points below are clarified it will help to streamline the presentation for readers such as us (and hopefully not cause problems for other readers...).

We thank the reviewer for the comment. We removed a large proviso to our discussion of **Fig. 2e**. Formerly we discussed how our experimental dataset was biased in that all communities contained *C. difficile* and discussed how this affected our results. We performed an additional experiment to measure communities without *C. difficile*. Now that we have these communities, the community set is no longer biased and this proviso in not needed. We believe this simplifies our discussion. In addition, we added clarifications to other parts of the manuscript based on suggestions by the reviewer below as well as clarifications suggested by the other reviewers. We believe this has improved the clarity of the manuscript.

3B: Throughout the paper I was often left wondering which observations / differences in conditions were due to differences in the steady state community as compared to transient dynamics as the community approaches steady state. For example, even in the pair-wise co-cultures the authors observed some (modest) differences in the fractions after co-culture depending upon the starting fractions. Do the authors have any evidence that this is due to bistability or is it simply due to the co-cultures starting from further away from the final, steady-state fractions? If it is the latter then I am hesitant to make too big of a deal of the observation. This may be particularly difficult to disentangle given that the fractions were measured after just 24 hr (one cycle, at line 119). In any case, there will always be a dependence on the initial fraction after just one cycle... More generally, when we talk about coexistence we are referring to coexistence at steady state rather than after just one cycle, but I acknowledge that perhaps that is not the only way to do it.

Also, in the context of communities I would have appreciated knowing whether there is multi-stability in the model (or again if the dependence on initial conditions is simply due to transients). I understand that this is difficult to establish with confidence in the experimental communities, but it would at least be good to be clear about what is happening in the model.

We thank the reviewer for asking about potential bistability in our community. As the reviewer mentions, it is difficult to establish with confidence in our experimental communities if there is multistability, and we did not design our experiments to answer this question. The time-series measurements of pairwise communities in **Fig. 1** (*C. difficile* and gut species inoculated at a 1:1 ratio) and **Appendix Fig. S1** (*C. difficile* and gut species were inoculated at a 1:9 ratio) showed that variation in the initial species proportion within this range did not result in significantly different community dynamics. Therefore, these data do not support the possibility of strong history-dependence/bistability within the range of tested initial species proportions. This experiment was not designed to test for bistability in the pairwise communities. Therefore, we would need to sample a wider range of initial species proportions (other species in addition to *C. difficile*) to determine if any communities have the potential for bistability/history-dependence. In our later experiment where we inoculated 3-4 member communities with high- or low-density of C. difficile, the high- and low-density conditions featured different abundance of C. difficile at 48 hours (**Fig. 3b, 4a**). We have added a new expanded view figure that indicates that the majority of these communities had reached saturation by this timepoint (**Fig. EV3a**). This suggests that inoculating with high- or low-density of C. difficile yielded distinct long-term abundances in these communities. Therefore, this would not be explained by transient effects due to starting communities farther from steady-state composition. This experimental data suggests that there could be long-term history dependence in our system.

In contrast, stability analysis of our model found that all subcommunities are monostable. We performed analysis of the stability of all non-negative equilibria for every possible sub-community of the 14-member community using our Full Model (16,383 total sub-communities). To this end, we determined all possible $2^N$ equilibria for each sub-community and evaluated the eigenvalues of the Jacobian matrix at each of the system's equilibria. Using this approach, we did not find any examples of bistability or multi-stability in our system for any sub-community. We previously showed that gLV models of pairwise communities can exhibit history-dependent transient behaviors in response to variation in initial species proportions for a prolonged period without requiring bistability if the inferred parameters are near a bistable parameter regime (PMID 29930200). Therefore, even in the absence of bistability/multi-stability, our model could be capable of predicting history-dependent behavior prior to reaching a steady-state due to variations in initial species proportions. We see this in the model simulations in **Fig EV3bcd**. We have modified our manuscript to describe the results of **Fig EV3a** as well as the stability analysis of our model.

Since we performed only three passages for characterizing the dynamics of pairwise communities in **Fig. 1e** and **Appendix Fig. S1**, we do not know if the communities have converged to a stable steady-state. In addition, the measurement at the end of each batch culture cycle may be a transient of the system since the communities are being perturbed by dilution at the beginning of each passage. Therefore, a community composition that is observed to persist at the end of each batch culture cycle in response to passaging may not represent the equilibrium of the system (steady-state of a gLV model). Given these limitations, we have removed the discussion of coexistence in pairwise communities due to variations in initial species proportions. Additionally, based on the reviewer's comment, we have modified our quantification of coexistence to use the composition after the third growth cycle instead of the first cycle, which may better represent the long-term behavior of the system.

Given these issues, it is a strong assumption that the abundances after one or two cycles are representative of the 'assembled community', which is understood as the long-term, stable community composition. It is very possible that after 48hr we are seeing community compositions that are still transient, significantly different from the equilibrium composition, especially for richer communities. This should be acknowledged in the text.

We agree with the reviewer. Since we did not perform passaging of multi-species communities (>2 species), we cannot conclude whether the variability in community composition at 48 hours due to variation in the initial abundance of *C. difficile* in the multi-species communities is due to potential multi-stability or inoculating the community further from the system's steady-state as the reviewer describes. We have now mentioned the caveat that the composition of communities for a single batch culture cycle for multi-species communities may deviate from the long-term composition of communities after multiple dilution cycles.

We note that the focus of our paper is understanding the interactions shaping the growth of *C. difficile* in this synthetic community and investigating the molecular basis of these interactions. The first batch culture cycle provided rich species dynamics for model parameter estimation since the system is further from steady-state. Communities that are closer to steady-state would likely exhibit less temporal variation and thus would be less informative for model parameterization. Therefore, we focused our efforts on characterizing many communities using a single batch culture cycle. Because measuring a single batch culture cycle is experimentally easier than

multiple dilution cycles, this allowed us to study the factors influencing *C. difficile* growth across a large number and therefore broad range of community contexts.

3C: I also felt that the main result of the paper-which is captured in the title-was not addressed in a very straightforward way. To me, the question to be addressed is: in order to successfully invade, is it best for CD to compete against a small number of species or against a large number of them? Ideally, the community being invaded would have already reached equilibrium / steady-state over multiple growth dilution cycles, but I understand that this could be debated (although perhaps it would have also made the presentation / interpretation of results simpler). Of course, the experiments have already been done and in the end are I think sufficient to make the claim.

We thank the reviewer for the comment. We agree that the previous title of the paper may not best represent the approach we used in this paper, which used computational modeling and high-throughput measurements of synthetic human gut communities to understand the ecological and molecular interactions shaping *C. difficile* growth. To better reflect our approach, we have changed our title to focus on a key result that connects our mechanistic studies. Our new title is "Negative interactions determine *C. difficile* growth in synthetic human gut communities."

The reviewer is correct that experimental designs to best represent invasion of non-resident species in the gut microbiome can be debated. In the healthy state, the gut microbiome is unlikely to operating in a steady-state since there are frequent perturbations to the system (e.g. nutrient influx through diet, transit through the gut, etc). Therefore, it is not clear whether passaging the communities over multiple batch culture dilution cycles represents a physiologically relevant "operating point" of the gut microbiome. In most of our experiments, *C. difficile* was introduced at the beginning (0 hour invasion). This experimental design could potentially represent invasion into the gut microbiome immediately following a perturbation that significantly disrupts the community (e.g. antibiotic treatment). In other experiments, we found that the diversity of the community tends to decrease over multiple passages (PMID 29930200). Therefore, introducing *C. difficile* into resident communities that have been passaged may reduce the range of richness levels that we could have explored in this study. We have modified our text to mention the physiological relevance of the timing of *C. difficile* introduction into the communities.

In this study, we did not test introduction of *C. difficile* into communities at steady state, but we did test introduction of *C. difficile* into communities six hours after the resident community was inoculated (**Fig. 4a,b,c**). We found that the abundance of *C. difficile* was similar when invaded at 0 or 6 hours in many communities (cluster of data points near the x=y line in **Fig. 4b**), indicating that the timing of invasion did not significantly impact the abundance of *C. difficile* in these communities. However, *C. difficile* abundance was different in a subset of communities when invaded at 0 versus 6 hours. Therefore, invasion timing can be important in determining *C. difficile* growth in some sub-communities.

Future work that explores the generalizability of the principles we found in our 0 and 6 hour invasions (i.e. *C. difficile* dependence on richness, dependence on propagule pressure, inhibition by acidifying communities, resource competition with *C. hiranonis*) by testing these principles in other situations such as communities after multiple dilution cycles, communities of larger sizes containing additional gut microbes, and communities *in vivo* will greatly add to our knowledge of principles of *C. difficile* invasion of gut communities.

MINOR SUGGESTIONS

3D: I strongly recommend an effort to convey the methods more clearly. In some cases, this might mean to include more details in the figure captions and results section. I found the paper difficult to read in this aspect, and I often had to go back and forth multiple times between the results section and the methods to understand how a given experiment was performed.

Specifically, how the absolute abundance of each species is calculated is not clear, and should be at least clearly stated that it is explained in the Method Data Analysis section. Preferably, it should be mentioned either in the text of the figure legend that the Absolute abundance was calculated by multiplying the relative abundance of an organism (according to the 16S-sequencing) by the OD600 of the sample. Then, a discussion like suggested later should be presented, as this way of estimating the total abundances of each species relies on some very serious assumptions.

We thank the reviewer for the comment. To improve readability, we have now numbered our community datasets as Exp1 through Exp8 throughout the text. Additionally, we now provide a summary description of each dataset in Appendix Table S1. We hope this will help aid readers throughout the paper.

We have now added a sentence in the Results section indicating how the absolute abundance is calculated (lines 102-104) and clarified this in the figures and figure legends using "Calculated OD600" to refer to the product of the relative abundance fraction and OD600 measurement.

We address your suggestion to add a discussion of assumptions at your comment below.

3E: Line 115: How many cycles were running? It seems like 3 but I would clearly state in the text

We have modified the text to indicate that 3 growth cycles were measured.

3F: Line 119 & Figure 1d&e: Is it always CD that is going extinct? It seems the FP is extinct in the co-culture but also as a monoculture. But it would be nice to know where the threshold for extinction goes- maybe add this to the figures? In many of the plots the experimental data does not agree with the model (CD and PC for example), so it may be good to mention here that the Full Model includes fits to the multi-species communities.

The reviewer is correct that it is not always *C. difficile* going extinct. FP, CH, CS, DP, and PC go extinct in coculture with *C. difficile*. We have added the threshold for extinction to **Fig. 1d,e**. We have also increased the figure size to make the data easier to view.

We added that the Full Model training data includes multi-species communities in the Figure legend.

3G: Fig. S2: How would different starting abundances of CD impact its abundance at 48hr simulation. Would it change the time till steady state reached?

We agree that the effect of starting abundances is an important aspect to consider. While **Appendix Fig. S2** shows the simulated 48 hour abundance of CD with just a single starting abundance, we address how different starting abundances of CD impact its abundance at 48 hours for 6 communities through experimental measurements in **Fig. 3b** and model simulations in **Fig. EV3bcd**. In these analyses, we tested a range of CD starting abundances from 5% of the initial community to 60% of the initial community. In both experiment and simulation, we found that increasing the starting abundance of CD does lead to an increase in 48 hour abundance of

CD, but the maximum abundance of CD depended on the community context. The sensitivity (EC50 of the response curve) of the 48 hour abundance to the starting abundance of CD also depended on the community context (**Fig. 3c**).

Our time-series data in **Fig EV3A** indicated that the abundance of *C. difficile* approached saturation by 48 hours in most of the communities. This suggests that inoculating with differing starting abundances of CD resulted in distinct long-term abundances in these communities. In contrast, our model predicts that propagule pressure is a transient effect and that communities initialized with different starting abundances all converge to a single steady state. We tested in our model if different starting abundances change the time it takes a community to reach steady-state. We simulated all possible subcommunities and calculated the time to reach steady state for starting CD abundances of 5% to 90% (**Fig. R4**). In the majority of sub-communities, changing the CD starting abundance does not alter the time to steady state (74% of the communities). Low-richness communities (2-5 members) were more likely to have an altered time to steady-state than high-richness communities.

We discuss the effect of propagule pressure in **Fig 3** and the effect of propagule pressure over time in the results text for **Fig EV3** and the Discussion section.

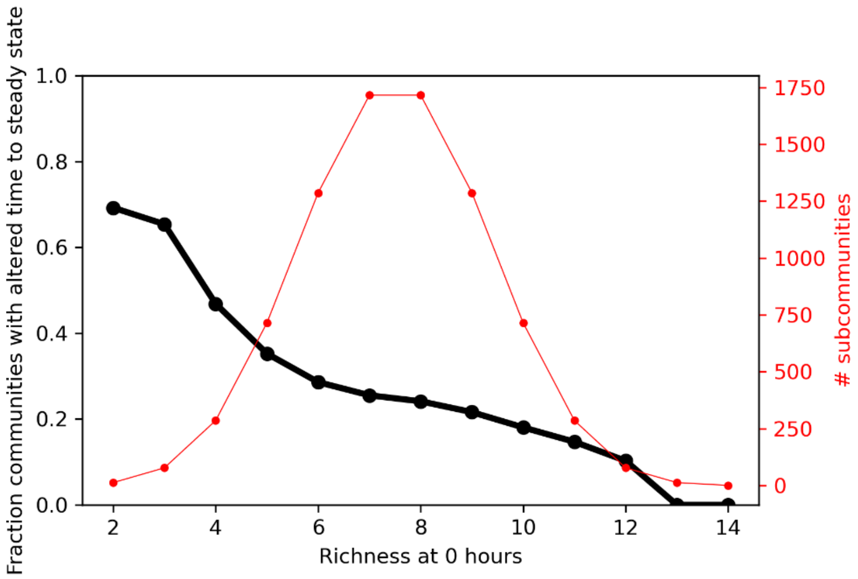


**Fig. R4**: **Altering initial *C. difficile* abundance changes the time to steady state in the minority of subcommunities.** Line plot of the fraction of communities with an altered time to steady state when *C. difficile* starting abundance is varied. Each community was simulated using the Full Model at 10 different *C. difficile* starting abundances (5, 10, 20, 30, 40, 50, 60, 70, 80, and 90% of initial community). The time to reach steady state was calculated in each simulation as the first timepoint *t* where the distance between the species vector at time *t* and the species vector at t=500 hours (sufficiently long to be at steady state for all communities) was less than 2% of the community abundance at t=500 hours. Communities were defined to have an altered time to steady state if the coefficient of variation of the time to steady state across the 10 starting *C. difficile* abundances was greater than 0.1.

3H: Fig. 3b: it would be helpful to have an indication of the initial richness of each community. It is a bit hard to get the richness from 3C, and Comm Q does not appear there.

We thank the reviewer for the suggestion. The initial richness is now indicated in the figure legend for **Fig. 3b**.

3I: Line 203-204. I am not sure that I agree with this interpretation, since all species do worse in more rich communities.

We assume the reviewer is referring to our hypothesis about the difference between PC and CH's relationship to richness in the experimental dataset versus the simulated dataset in **Fig. 2e**. Both PC and CH had lower abundances in the experimental dataset. In the original text, we hypothesize this difference was due to *C. difficile*, which inhibits both PC and CH, being present in all communities in the experimental dataset, but only a fraction of the communities in the simulated dataset. We note that this hypothesis was in response to PC and CH showing the largest difference in trends between experimental dataset and simulated dataset, not showing a unique relationship with richness.

In our revision, to provide a deeper understanding of the trends in **Fig. 2e**, we carried out new experiments to measure 110 additional communities that do not contain *C. difficile* and added these data to the existing communities in **Fig. 2e** (original communities are circles, new communities are triangles). The experimental data set now contains approximately equal numbers of communities with and without *C. difficile*. This removes any potential *C. difficile* bias between the experimental dataset and simulated dataset. We have therefore removed our discussion of the potential bias due to higher numbers of communities containing *C. difficile*.

We also note that it is not true that "all species do worse in more rich communities". We now quantify the relationship between abundance and richness for each species by the decay constant from an exponential decay model fit to the data. We find that not all species do worse in higher richness communities: species BH, EL, DP and BV show no dependence on richness in the experimental datasets (decay constant $b < 0.05$, **Fig. 2f**). We have revised our manuscript based on the new data and quantification of the strength of the relationship between abundance and species richness.

3J: Line 251: are these results shown anywhere?

The EC50 results are shown in **Fig. 3c**. We have corrected a typo that previously referenced Community N instead of Community R.

3K: Lines 253-261 (Fig. S5): - this simulation does not seem to agree with the data presented on Fig. 3b. Can the authors provide an explanation for this? Also, doesn't this figure suggest the experimental data should be taken from longer times than 48hr? Though most of the plots seem independent of the initial inoculum at this time, some do.

**Fig. S5** has been renumbered to **Fig. EV3**. For Communities H, I and P, the model (**Fig. EV3c**) and data (**Fig. 3b**) qualitatively agree, both showing a saturating trend where the abundance of *C. difficile* increases as initial fraction of *C. difficile* in the community increases until it saturates at a maximum threshold. The limited datapoints we have for Community Q (**Fig. 3b**) also qualitatively agree with the model (**Fig. EV3c**) as they both show no change in *C. difficile* abundance when density varies between 0.3 and 0.6.

The model incorrectly predicts the abundance of *C. difficile* in Community R and the full community at 48 hours. The model predicts *C. difficile* will be extinct (**Fig. EV3c**) however, in our data we see presence of *C. difficile* in the community at this timepoint (**Fig. 3b**). While the model

explains the majority of our data (**Fig. EV1a**, goodness of fit r=0.89), it does not accurately describe trends in all communities. While the model training data includes resolved growth curves of each monospecies, the majority of multispecies communities included have only a single timepoint of data (with the exception of Fig. EV3a which shows time-series measurements of multi-species communities). Therefore, the model could be improved in the future by adding additional time-series measurements. Additionally, the gLV model may not accurately represent community dynamics as a function of initial species density since there are many contributions of history-dependence that are not accounted for in the gLV model (e.g. changes in inter-species interactions due to variations in initial species abundance). The steady-state behavior of the gLV model has limited flexibility due to the mathematical structure of the model. For example, bistability which could potentially explain history-dependent behaviors due to variations in initial abundance is infrequent in gLV models fit to experimental data (PMID 29930200). There are many advantages of gLV models including their simple mathematical structure and ability to infer parameters from measurements of community composition. However, we should not expect the gLV model to explain all complex microbial community behaviors due to the assumptions made by the model (e.g. fixed inter-species interaction coefficients). Nevertheless, the gLV model can provide key insights into inter-species interactions and community assembly (PMID 29930200, 34059668)

We performed the experiment at 48 hours for consistency with our other experiments. The effect of propagule pressure over time is an interesting question, which we now discuss in the Discussion section. We note that in the model simulations, the effect of propagule pressure is transient and the abundance of *C. difficile* is independent of propagule pressure at later timepoints (ranges from ~24 to ~96 hours, exact time depends on community) (**Fig EV3CD**). However, our time-series data in **Fig EV3A** indicated that the abundance of *C. difficile* approached saturation by 48 hours in most of the communities. This suggests that inoculating with high- or low-density of *C. difficile* yielded distinct long-term abundances in these communities. Thus, our data suggests that the observed effects of propagule pressure results in history-dependent behavior that persists long-term. Therefore the transient effect predicted by the gLV model may not be an accurate depiction of our system, potentially due to reasons discussed in the paragraph above. Future work will determine the long-term effects of propagule pressure on *C. difficile* abundance using passaging or continuous culture and develop models that can accurately predict these history-dependent behaviors.

3L: Somewhere in the main text, a discussion on the estimated reliability on the measurements of CD abundance (OD), including factors that might affect the results. For example: are cells from all species considered to contribute equally to OD? This is only one additional source of uncertainty added to the many that 16S sequencing already has, but I believe that the discussion is worthwhile.

We estimated the reliability of our 16S method to measure CD abundance using mixed cultures. We created communities with 90% species 1 and 10% species 2 (based on OD600 measurements) and then measured the relative abundance by 16S sequencing. The 16S relative abundances for species 1 ranged from 85% to 97%, within 10% of the true value of 90%, giving us confidence in our ability to accurately quantify the relative abundance of 2-member communities containing CD. Note that our gLV model was trained on primarily lower species richness communities including pairwise communities with CD and thus it is likely that minimal bias was introduced using these data (**Fig. 1e**).

We also investigated 16S bias in 14-member mixed cultures (**Fig. R1b**). We created six different mixed cultures with various compositions and then measured the relative abundance by 16S

sequencing immediately after mixing (no growth). We observed a linear relationship between expected OD600 and calculated OD600 for CD and all other species, indicating that we can observe variation in species abundance over a wide range and changes in expected OD600 were proportional to calculated OD600 (no saturation). CD had a slope of 1.28, which means that CD abundance was overrepresented by a moderate factor of 1.28 in the 14-member community. CH, BH, and EL were underrepresented based on expected OD600. Interestingly, there was no such underrepresentation of these species in the CD-CH, CD-BH, or CD-EL pairs (**Fig. R1a**). This suggests 16S bias may change depending on the community context. As such, we do not apply any bias-correction for species with substantial bias based on Fig. R1b since the bias factor could vary across different community contexts.

There are two points that make us confident in our biological conclusions in the manuscript despite inevitable biases in 16S sequencing that impacts every microbiome sequencing study based on 16S sequencing:

1) We confirm results obtained with 16S sequencing with conditioned media experiments. The inhibition of CD by CommI, CommH, CommK observed via 16S sequencing (**Fig. 4d**) was confirmed with conditioned media experiments in **Fig. 4e**. The inhibition of CD by CH in CD-CH, CommE, and CommO observed via 16S sequencing (**Fig. 5b**) was confirmed in conditioned media experiments (**Appendix Fig. S6**). Previous work using this 16S method found that 75% of interactions determined using 16S sequencing were in qualitative agreement with conditioned media experiments (Fig. S21 in PMID 29930200). It is possible that some of conditions that showed qualitative disagreement could be due to 16S bias. However, that is an overestimate, since there are notable differences between direct species interactions in community vs indirect interaction in conditioned media experiments (e.g. changed metabolite utilization in the presence of a second species) which contribute to the discrepancy.
2) We found a linear relationship between the true value and calculated 16S relative abundance for all species, even including the underrepresented set (**Fig. R1b**). This means that even in cases where the absolute abundance is biased, the fold change in species absolute abundance between conditions is accurate. In the manuscript, our conclusions are based on changes in species abundance between conditions and thus our conclusions are not based on a single condition. For example, we look at the trend of decreasing CD with increasing richness, trend of increasing CD with increasing propagule pressure, and trend decreasing CD with increasing CH. These trends would be robust to under- or over-representation due to the linear relationships in **Fig. R1b**.

To comment on your specific example: we do assume that cells from all species contribute equally to OD600. The alternative to this assumption is to measure the CFU per mL per OD (CFU $mL^{-1}$ $OD^{-1}$) for each monospecies by CFU counting and apply a correction factor to normalize species with that feature a higher or lower CFU $mL^{-1}$ $OD^{-1}$. Unfortunately, CFU counting has its own biases due to selection pressure of solid media, cell adhesion which undercounts cell clumps as single colonies, and significant variation depending on the growth stage of the culture at time of plating. Therefore, we use the simple assumption that cells from all species contribute equally to OD600. Any bias from this assumption should apply equally across all of the experiments, and the fold change between conditions would remain unaffected. Therefore, as discussed in point 2 above, our conclusions remain unaffected.

We appreciate the suggestion to include a discussion of the assumptions in our method in the main text. We have included a discussion of these assumptions and biases in our methods in the Discussion and new **Appendix Figure S7**.

3M: How are the error bars computed in Fig. 4A?

The error bars in **Fig. 4a** are the standard deviation of the biological replicates (n=2 to n=3). This is indicated in the figure legend.

3N: Figure 5: It would be nice to discuss why adjusting both pH and Glucose in Community E give significantly more inhibition than adjusting just glucose. Given that adjusting pH only does not have an impact, this result is surprising.

We have edited **Fig. 5c** to include new analysis from exo-metabolomics experiments we have performed as suggested by other reviewers. As such, this figure is no longer discussed in the manuscript. However, we will explain here.

*C. difficile* monospecies grows better in more basic media (**Fig. 4c**). Since CommE and CommO supernatants are more basic than the starting media, when we pH adjust the supernatant to the more acidic pH, *C. difficile* is inhibited due to the more acidic environment. This expected increase in inhibition compared to control is observed when we lower the pH in pH-adjusted CommO, pH- and glucose-adjusted CommO, and pH- and glucose-adjusted CommE. Surprisingly, we don't observe any significant increase in the strength of inhibition in pH-adjusted CommE. It is possible that there is a threshold of resources required before *C. difficile* grows well enough to be impacted by pH. Without glucose addition, the growth of *C. difficile* in CommE may be low enough that pH doesn't have a significant effect.

Thank you for sending us your revised manuscript. We have now heard back from two of the three reviewers who were asked to evaluate your revised study. In the interest of time we have decided to proceed with making a decision based on the two available reports. As you will see below, the reviewers are satisfied with the performed revisions and are supportive of publication.

Before we formally accept the study for publication we would ask you to address some remaining editorial issues listed below.

REFEREE REPORTS
--------------------------------------------------------------------------------

Reviewer #1:

The authors have carefully taken my comments on board and revised the paper as I would have hoped in response. I am happy to support publication.

Reviewer #3:

I believe that the authors have adequately addressed the questions and concerns raised by me and the other referees.

In particular, while the methodology is similar to that used in their previous study, the inclusion of C. difficile-the most famous pathogen in studies of the human gut microbiome-makes this study of interest to a wide range of researchers.

Also, we and others often find that fast-growers are outcompeted by slow-growers, so this is an outcome that cannot be taken for granted based on standard microbiological principles (indeed, I would argue that the prevalence of this expectation is mistaken).

Finally, I would respond to the authors that even in batch culture with dilution you can define a stable steady state if the community state / composition is the same at the end of each cycle (even if it is changing over the course of the day).

The authors have made all requested editorial changes.

**ACCEPTED** **30th Sep 2021**

Thank you again for sending us your revised manuscript. We are now satisfied with the modifications made and I am pleased to inform you that your paper has been accepted for publication.

EMBO PRESS

YOU MUST COMPLETE ALL CELLS WITH A PINK BACKGROUND ➜

PLEASE NOTE THAT THIS CHECKLIST WILL BE PUBLISHED ALONGSIDE YOUR PAPER

Corresponding Author Name: Ophelia Venturelli

Journal Submitted to: Molecular Systems Biology
Manuscript Number: MSB-2021-10355

## Reporting Checklist For Life Sciences Articles (Rev. June 2017)

This checklist is used to ensure good reporting standards and to improve the reproducibility of published results. These guidelines are consistent with the Principles and Guidelines for Reporting Preclinical Research issued by the NIH in 2014. Please follow the journal's authorship guidelines in preparing your manuscript.

### A- Figures
### 1. Data
### The data shown in figures should satisfy the following conditions:

- the data were obtained and processed according to the field's best practice and are presented to reflect the results of the experiments in an accurate and unbiased manner.
- figure panels include only data points, measurements or observations that can be compared to each other in a scientifically meaningful way.
- graphs include clearly labeled error bars for independent experiments and sample sizes. Unless justified, error bars should not be shown for technical replicates.
- if n< 5, the individual data points from each experiment should be plotted and any statistical test employed should be justified
- Source Data should be included to report the data underlying graphs. Please follow the guidelines set out in the author ship guidelines on Data Presentation.

### 2. Captions
### Each figure caption should contain the following information, for each panel where they are relevant:

- a specification of the experimental system investigated (eg cell line, species name).
- the assay(s) and method(s) used to carry out the reported observations and measurements
- an explicit mention of the biological and chemical entity(ies) that are being measured.
- an explicit mention of the biological and chemical entity(ies) that are altered/varied/perturbed in a controlled manner.

- the exact sample size (n) for each experimental group/condition, given as a number, not a range;
- a description of the sample collection allowing the reader to understand whether the samples represent technical or biological replicates (including how many animals, litters, cultures, etc.).
- a statement of how many times the experiment shown was independently replicated in the laboratory.
- definitions of statistical methods and measures:
  - common tests, such as t-test (please specify whether paired vs. unpaired), simple χ2 tests, Wilcoxon and Mann-Whitney tests, can be unambiguously identified by name only, but more complex techniques should be described in the methods section;
  - are tests one-sided or two-sided?
  - are there adjustments for multiple comparisons?
  - exact statistical test results, e.g., P values = x but not P values < x;
  - definition of 'center values' as median or average;
  - definition of error bars as s.d. or s.e.m.

Any descriptions too long for the figure legend should be included in the methods section and/or with the source data.

**In the pink boxes below, please ensure that the answers to the following questions are reported in the manuscript itself. Every question should be answered. If the question is not relevant to your research, please write NA (non applicable). We encourage you to include a specific subsection in the methods section for statistics, reagents, animal models and human subjects.**

## USEFUL LINKS FOR COMPLETING THIS FORM

http://www.antibodypedia.com
http://1degreebio.org
http://www.equator-network.org/reporting-guidelines/improving-bioscience-research-repor
http://grants.nih.gov/grants/olaw/olaw.htm
http://www.mrc.ac.uk/Ourresearch/Ethicsresearchguidance/Useofanimals/index.htm
http://ClinicalTrials.gov
http://www.consort-statement.org
http://www.consort-statement.org/checklists/view/32-consort/66-title
http://www.equator-network.org/reporting-guidelines/reporting-recommendations-for-tumo
http://datadryad.org
http://figshare.com
http://www.ncbi.nlm.nih.gov/gap
http://www.ebi.ac.uk/ega
http://biomodels.net/
http://biomodels.net/miriam/
http://jjj.biochem.sun.ac.za
https://osp.od.nih.gov/biosafety-biosecurity-and-emerging-biotechnology/
http://www.selectagents.gov/

### B- Statistics and general methods

Please fill out these boxes ➜ (Do not worry if you cannot see all your text once you press return)

| | |
|---|---|
| 1.a. How was the sample size chosen to ensure adequate power to detect a pre-specified effect size? | Sample sizes were chosen based on limitations of experimental throughput as increased number of biological replicates would have reduced the number of possible different communities that could be observed. We measured n=3 biological replicates of each community but due to contamination exclusion, a small number of conditions had a final sample size of n=1 or n=2 (See Appendix Table S4 for detailed information). When we analyze trends across communities, the statistical test are performed on groups of communities, so the number of different communities determined our statistical power in these comparisons. |
| 1.b. For animal studies, include a statement about sample size estimate even if no statistical methods were used. | NA |
| 2. Describe inclusion/exclusion criteria if samples or animals were excluded from the analysis. Were the criteria pre-established? | Individual samples were excluded from the analysis if they were determined to be contaminated by species not intended to be in the sample. Contamination was determined to be any species >1% of the sample. This cutoff was chosen based on the ratio of typical background to typical read depth for our samples. |
| 3. Were any steps taken to minimize the effects of subjective bias when allocating animals/samples to treatment (e.g. randomization procedure)? If yes, please describe. | No. |
| For animal studies, include a statement about randomization even if no randomization was used. | NA |
| 4.a. Were any steps taken to minimize the effects of subjective bias during group allocation or/and when assessing results (e.g. blinding of the investigator)? If yes please describe. | For the community design experiments, the HPLC analysis was carried out in a blinded manner. The samples were labeled only with a numerical identifier and given to a separate researcher for measurement. Otherwise, while the experimental allocations were known to the experimental investigator because the same individual designed the experiments, the large number of samples were labeled using a code and thus were practically unknown prior to analysis of the final aggregated data. |
| 4.b. For animal studies, include a statement about blinding even if no blinding was done | NA |
| 5. For every figure, are statistical tests justified as appropriate? | As necessary, we identify our statistical tests as unpaired t-tests in the figure legends. |

| | |
|---|---|
| Do the data meet the assumptions of the tests (e.g., normal distribution)? Describe any methods used to assess it. | Our experiments involve large numbers of cells, which according ot the central limit theorem will be approximately Gaussian (normal). Our measurement error (technical error) is also Gaussian because it is caused by various sources of noise and does not contain systematic bias. |
| Is there an estimate of variation within each group of data? | Yes, we plot individual replicates to assess variance. In cases where plotting individual replicates is not feasible (stacked bar plots and busy scatterplots), standard deviation is indicated using error bars. |
| Is the variance similar between the groups that are being statistically compared? | Yes, because compared groups are same sample types and are measured using same method. |

## C- Reagents

| | |
|---|---|
| 6. To show that antibodies were profiled for use in the system under study (assay and species), provide a citation, catalog number and/or clone number, supplementary information or reference to an antibody validation profile. e.g., Antibodypedia (see link list at top right), 1DegreeBio (see link list at top right). | NA |
| 7. Identify the source of cell lines and report if they were recently authenticated (e.g., by STR profiling) and tested for mycoplasma contamination. | NA |

* for all hyperlinks, please see the table at the top right of the document

## D- Animal Models

| | |
|---|---|
| 8. Report species, strain, gender, age of animals and genetic modification status where applicable. Please detail housing and husbandry conditions and the source of animals. | NA |
| 9. For experiments involving live vertebrates, include a statement of compliance with ethical regulations and identify the committee(s) approving the experiments. | NA |
| 10. We recommend consulting the ARRIVE guidelines (see link list at top right) (PLoS Biol. 8(6), e1000412, 2010) to ensure that other relevant aspects of animal studies are adequately reported. See author guidelines, under 'Reporting Guidelines'. See also: NIH (see link list at top right) and MRC (see link list at top right) recommendations. Please confirm compliance. | NA |

## E- Human Subjects

| | |
|---|---|
| 11. Identify the committee(s) approving the study protocol. | NA |
| 12. Include a statement confirming that informed consent was obtained from all subjects and that the experiments conformed to the principles set out in the WMA Declaration of Helsinki and the Department of Health and Human Services Belmont Report. | NA |
| 13. For publication of patient photos, include a statement confirming that consent to publish was obtained. | NA |
| 14. Report any restrictions on the availability (and/or on the use) of human data or samples. | NA |
| 15. Report the clinical trial registration number (at ClinicalTrials.gov or equivalent), where applicable. | NA |
| 16. For phase II and III randomized controlled trials, please refer to the CONSORT flow diagram (see link list at top right) and submit the CONSORT checklist (see link list at top right) with your submission. See author guidelines, under 'Reporting Guidelines'. Please confirm you have submitted this list. | NA |
| 17. For tumor marker prognostic studies, we recommend that you follow the REMARK reporting guidelines (see link list at top right). See author guidelines, under 'Reporting Guidelines'. Please confirm you have followed these guidelines. | NA |

## F- Data Accessibility

| | |
|---|---|
| 18: Provide a "Data Availability" section at the end of the Materials & Methods, listing the accession codes for data generated in this study and deposited in a public database (e.g. RNA-Seq data: Gene Expression Omnibus GSE39462, Proteomics data: PRIDE PXD000208 etc.) Please refer to our author guidelines for 'Data Deposition'.<br><br>Data deposition in a public repository is mandatory for:<br>a. Protein, DNA and RNA sequences<br>b. Macromolecular structures<br>c. Crystallographic data for small molecules<br>d. Functional genomics data<br>e. Proteomics and molecular interactions | We provide a "Data Availability" section in the text. |
| 19. Deposition is strongly recommended for any datasets that are central and integral to the study; please consider the journal's data policy. If no structured public repository exists for a given data type, we encourage the provision of datasets in the manuscript as a Supplementary Document (see author guidelines under 'Expanded View' or in unstructured repositories such as Dryad (see link list at top right) or Figshare (see link list at top right). | We provide datasets as Expanded View Dataset3 and Expanded View Dataset 4. |
| 20. Access to human clinical and genomic datasets should be provided with as few restrictions as possible while respecting ethical obligations to the patients and relevant medical and legal issues. If practically possible and compatible with the individual consent agreement used in the study, such data should be deposited in one of the major public access-controlled repositories such as dbGAP (see link list at top right) or EGA (see link list at top right). | NA |
| 21. Computational models that are central and integral to a study should be shared without restrictions and provided in a machine-readable form. The relevant accession numbers or links should be provided. When possible, standardized format (SBML, CellML) should be used instead of scripts (e.g. MATLAB). Authors are strongly encouraged to follow the MIRIAM guidelines (see link list at top right) and deposit their model in a public database such as Biomodels (see link list at top right) or JWS Online (see link list at top right). If computer source code is provided with the paper, it should be deposited in a public repository or included in supplementary information. | We provide access to computational models in a Github repository. |

## G- Dual use research of concern

| | |
|---|---|
| 22. Could your study fall under dual use research restrictions? Please check biosecurity documents (see link list at top right) and list of select agents and toxins (APHIS/CDC) (see link list at top right). According to our biosecurity guidelines, provide a statement only if it could. | NA |
